# Electrophysiological Signatures of Sarcopenia: A Systematic Review of sEMG Features, Fatigue Indices and AI-Based Classifiers

**DOI:** 10.3390/s26165121

**Published:** 2026-08-13

**Authors:** Karen-Victoria Villanueva-De-Luna, Laura-Ivoone Garay-Jimenez, Joel Lomelí-González, Javier M. Antelis, Omar Mendoza-Montoya, Blanca-Alicia Rico-Jiménez, Blanca Tovar-Corona

**Affiliations:** 1 Instituto Politécnico Nacional, Unidad Profesional Interdisciplinaria en Ingeniería y Tecnologías Avanzadas (UPIITA), Mexico City 07340, Mexico; kvillanuevad1900@alumno.ipn.mx (K.-V.V.-D.-L.); lgaray@ipn.mx (L.-I.G.-J.); bricoj@ipn.mx (B.-A.R.-J.); 2 Instituto Politécnico Nacional, Escuela Superior de Medicina (ESM), Mexico City 07738, Mexico; jlomelig@ipn.mx; 3 Tecnologico de Monterrey, Escuela de Ingeniería y Ciencias, Monterrey 64849, Mexico; mauricio.antelis@tec.mx (J.M.A.); omendoza83@tec.mx (O.M.-M.)

**Keywords:** surface electromyography, sarcopenia, machine learning, neuromuscular function, aged, muscle strength, hand grip, meta-analysis

## Abstract

Age-related sarcopenia involves structural and functional neuromuscular changes. Electrophysiological measures from surface electromyography (sEMG) capture activation dynamics, spectral fatigue indices and motor unit properties that may constitute objective signatures of sarcopenia. The objective of this work is to systematically review sEMG features, fatigability metrics and AI-based classification/regression approaches reported for sarcopenia assessment between 2019 and 2026. PRISMA guidelines were followed, and IEEE Xplore, PubMed and Scopus were searched for open access human studies. Extracted information comprised sample characteristics, muscles and tasks, signal acquisition and preprocessing, extracted time/frequency/time–frequency and motor unit features, fatigue metrics, machine learning pipelines, validation schemes and dataset accessibility. Studies were classified into activation, fatigue, ML, and neural control groups; risk of bias was assessed. A total of 12 studies fulfilled the inclusion criteria. Recurrent electrophysiological signatures included reduced distal activation with compensatory proximal recruitment and higher antagonist co-activation; diminished MF/IMDF fatigue slopes indicative of Type II fiber loss and altered motor unit recruitment; motor unit analyses revealed decreased discharge rates and larger MUAP amplitudes. AI-based models combining multidomain features (time, spectral, CWT/EMD, and motor unit metrics) yielded reasonable screening performance (AUC/accuracy 0.73–0.89) when using robust feature selection and explainability tools. Heterogeneity in acquisition, normalization, small cohorts and sparse data sharing limited comparability and external validity. The findings indicate that sEMG-derived electrophysiological signatures are promising for sarcopenia detection and monitoring. To translate signatures into reliable clinical tools, standardized protocols, larger shared datasets, multimodal features, including motor unit metrics, and rigorous external validation of AI models are required.

## 1. Introduction

Skeletal muscle is functionally organized into motor units, each representing the basic component responsible for generating muscle activity. A simple motor unit (SMU) consists of a motoneuron and the muscle fibres it innervates. These units act as bioelectrical sources within a volume conductor that includes other muscle fibres, both active and inactive. Active fibres generate an extracellular potential field with a short duration of approximately 3 to 15 ms and an amplitude ranging from 20 to 2000 μV, depending on the size of the motor unit [1]. In a healthy subject, resting muscle does not exhibit electrical activity detectable by surface electromyography (sEMG). However, moderate voluntary contraction activates one or a few motor units, with low discharge frequencies (approximately 1–2 impulses per second). As muscle force increases, two phenomena are observed: the recruitment of previously inactive motor units and an increase in the firing rate of already activated units. This process reflects the sequential activation of type I muscle fibres during the initial stages and type II fibres during more intense contractions. With increasing muscle contraction, additional motor units are activated, leading to the so-called interference pattern, in which the bioelectrical signals of multiple motor units overlap, making it difficult to distinguish individual potentials. This pattern reflects the spike density and average amplitude of the motor unit potentials involved. During maximal effort, the muscle generates an interference signal with an sEMG amplitude of approximately 2–4 mV, indicating the simultaneous activation of numerous motor units. This process of muscle activation is particularly relevant in the context of ageing. Recent studies have shown that ageing is associated with a decline in both the quantity and quality of muscle fibres, affecting muscle strength and function. Sarcopenia, a musculoskeletal disorder characterized by the progressive loss of muscle mass and strength, is one of the leading causes of frailty in older adults. At the histological level, it is associated with reduced muscle fibre size, replacement by adipose tissue, and alterations in motor unit firing rates [2,3].

Unlike techniques that focus solely on muscle mass, such as dual-energy X-ray absorptiometry (DXA) or bioelectrical impedance analysis, sEMG provides a functional assessment of muscle activity. Evidence suggests that muscle strength and functionality may be impaired before significant loss of muscle mass becomes evident. Surface electromyography provides a non-invasive assessment of neuromuscular dynamic functionality, enabling the characterization of changes in muscle quality, motor unit recruitment, and contraction efficiency [2].

However, despite the increasing use of sEMG to assess neuromuscular function in older adults, there is still no clear consensus on the most relevant signal features, acquisition protocols, and analysis methods for assessing sarcopenia. Therefore, the objective of this systematic review is to identify and compare the methodologies currently used for sEMG-based sarcopenia assessment in older adults, including signal processing techniques, the main characteristics of feature extraction, machine learning approaches, evaluation metrics, and reported outcomes.

## 2. Materials and Methods

### 2.1. Search Strategy

The IEEE Xplore, PubMed, and Scopus databases were searched using the PICO framework and criteria for conducting a systematic review in accordance with the Preferred Reporting Items for Systematic Reviews and Meta-Analyses (PRISMA) guidelines. To identify MeSH terms, the following statement was used: “Sensory and motor changes in elderly people with and without sarcopenia, measured with electromyography”, from which the terms [“Aged”, “Sarcopenia”, “Electromyography”, “Health Status”] and additional terms [“Humans”] were identified.

The primary terms were selected, as they directly represent the population, condition, measurement technique, and general health context of interest. The additional term “Humans” was incorporated to ensure the exclusion of animal studies. To improve search sensitivity and account for variations in terminology, “Elderly”, “Ageing”, and “EMG” were also considered.

Only open-access articles published between January 2019 and March 2026 were included, using the following search string: [“Healthy” AND “Humans” AND (“Elderly” OR “Aged” OR “Aging”) AND (“Electromyography” OR “EMG”) AND “Sarcopenia”]. In all cases, searches were conducted based on titles and abstracts. Open access was selected for transparency and accessibility for academics and clinicians. However, a search without the restriction of open access was carried out, and an analysis for sensitivity, found in File S1, demonstrates that the main conclusions remain and that the information extracted from open access publications does not exclude any crucial publication. A table for each of the queries in the three databases for all the filters can also be found in File S1.

Additionally, to further support the discussion of the findings, the same search strategy was repeated, filtering only for systematic reviews and meta-analyses within the same time range. This second stage aimed to contextualize the findings and to more clearly contrast existing evidence synthesized in the secondary literature with the focus of the present review.

### 2.2. Study Selection

The selection of articles was conducted using the search query in March 2026 by K.V.V.D.L and verified by B.T.C. Any disagreements were resolved by consensus among all authors. Then, in July 2026, L.I.G.J., as an independent reviewer, repeated the search using the same query and documented the entire process. After the independent application of the PRISMA selection criteria, author consensus was reached for both results. The automatically obtained results were exported in CSV format. Initially, duplicate records were removed. Subsequently, titles and article types were screened. The following inclusion criteria were applied: (a) published within the last seven years; (b) studies involving human subjects; (c) studies focused on the elderly; (d) open access studies; and (e) studies employing electromyography for assessment.

Articles were excluded based on the following criteria: (a) animal studies; (b) studies involving minors; (c) studies including older adults with comorbidities non-related to motor performance; and (d) publications identified as books, conference proceedings, comments, editorials, case reports, and systematic reviews. Criteria (c) was applied to focus on sarcopenia as the primary sensorimotor condition under investigation, although this may limit the generalizability of the findings to populations with multimorbidity. Two reviewers independently evaluated the papers (K.V.V.D.L. and L.I.G.J.) according to these criteria. Kappa and AC1 methods were computed, and low concordance was obtained; therefore, discrepancies in study selection were discussed among the authors to reach consensus. This analysis is in File S2 [4,5,6,7].

### 2.3. Data Extraction and Synthesis of Results

Data from the included studies were extracted into Excel. Records were manually exported from each database in CSV format based on the selected results and were subsequently consolidated into a single dataset. The following information was collected: study identifiers (author(s), year of publication, DOI), study characteristics (study population, total number of recruited participants, and number of analyzed participants), assessment methods, and main findings. To ensure consistency, the extracted data were reviewed and curated during group discussions among the authors. The names of the files, the filters used, the number of articles found, and the consulting time are in File S1 for the primary and secondary consultations. An additional search was carried out with the query excluding the elderly without sarcopenia and another including the elderly with sarcopenia but excluding the healthy keyword for analyzing the restriction effect.

### 2.4. Quality Assessment

The quality of all included studies was independently assessed by two reviewers (K.V.V.D.L. and L.I.G.J.). Any disagreements were subsequently resolved through consensus during structured discussion sessions among authors. The assessed parameters were: (a) Title (indication of the type of article); (b) Abstract (including background, methodology, results, and discussion); (c) Introduction (clear rationale and study objectives); (d) Methodology (protocol registration, eligibility criteria, data collection process, data items, effect measures, and additional analyses); (e) Results (study characteristics, individual study results, and additional analyses); (f) Discussion (summary of evidence, strengths and limitations, conclusions, and implications); and (g) Funding. Due to the qualitative nature of the review, no effect measures were defined.

### 2.5. Analysis Process

Due to the methodological heterogeneity of the included studies, comparisons could not be performed solely on the basis of the muscle evaluated or the technique employed. The present review adopts a function-oriented framework, grouping studies according to the primary question addressed by the sEMG analysis. Additionally, the inclusion criteria were specifically focused on older adults, ensuring that the comparison reflects age-related neuromuscular changes associated with sarcopenia. Specifically, four guiding research questions were defined: (a) How does muscle activation contribute to functional performance in the context of sarcopenia? (b) How are fatigue and fatigability currently assessed in sarcopenia and the elderly using sEMG? (c) How is sEMG combined with machine learning for sarcopenia identification or classification? And (d) How does neural control relate to functional coordination in the progression of sarcopenia?

The evidence from the 12 included studies was analyzed based on these questions. Some studies address more than one of these questions, while others focus more specifically on a single aspect. Thus, the grouping reflects alignment with the main research question rather than mutually exclusive study classifications. When a study addressed multiple research questions, it was classified according to the question to which it provided the most substantial contribution, based on the predominant focus of its findings.

Song, K. et al. (2024) and Kumar, K. S. (2024), reported medians and interquartile ranges [8,9]. These were converted to means and standard deviations by Wan et al. (2014) [10], with optimization from Luo et al. (2018) [11]. When minimum and maximum values were provided, estimations followed the methods of Wan et al. (2014) and Hozo et al. (2005) [10,12]. Data were converted only if the distribution was approximately symmetric, first assessed by Wan et al. [10] and then verified by Shi et al. (2020) [13]. The mean was estimated as Mean ≈ (Q1 + M + Q3)/3, assuming symmetry. Standard deviation estimation used SD ≈ (Q3 – Q1)/1.35, assuming a Gaussian distribution.

Additionally, each group analysis includes a joint qualitative synthesis of the contributions, based on the comparison of reported outcomes, sEMG-derived variables, and the physiological interpretations proposed by the authors. Then, a cross-group comparative analysis was conducted in the discussion to integrate findings.

Moreover, the findings were contrasted with existing systematic reviews addressing related aspects of neuromuscular function and aging. This comparison was conducted to identify similarities and differences among reported findings to situate the present review within the broader body of evidence and to highlight its specific contribution.

Finally, the methodological quality of the included studies was evaluated using the GRADE [14] Risk of Bias framework, following two evaluations and consensus based on the RoB results, selected according to the type of study. The GRADE method considered the domains of random sequence generation, allocation concealment, blinding procedures, incomplete outcome data, selective reporting, and other potential sources of bias. Each study was classified as presenting a low, unclear, or high risk of bias based on the methodological information reported in the corresponding articles. The complete analysis is shown in File S3 [15,16,17,18,19,20,21,22,23,24,25,26,27]; according to the study type, a principal RoB was selected, and a complementary test is included when studies are hybrid. Additionally, the overall judgment and its rationale are included.

### 2.6. Clinical Reference Test Hand Grip Associated with Sensorimotor Response

Random and fixed effects meta-analyses of mean differences were performed for three independent elderly cohorts: Elderly-Female, Elderly-Male, and Elderly. Analyses used DerSimonian–Laird (DL) and restricted maximum likelihood (REML) estimators for the between-study variance ($\tau^{2}$) of the clinical reference as a complement to support this. Forest plots were created for each group and estimator. Because some rows in the original input represented subgroups and combined estimates from the same studies, we avoided a pooled “Overall” analysis across all rows; instead, we report results only for the three independent groups. A sensitivity analysis excluding Hu (2021) was implemented for the Elderly group [28]. Results, per-study tables, and summary tables are available in the outputs folder.

The data input is the eight rows representing three independent study groups. They were defined as subgroup entries in the script MetaAnalysis_groups_final.m available on GitHub. The script groups are Elderly Female (T1), Elderly Male (T2), and Elderly (T3). Each group was analyzed independently. For each group, the script computes: k (number of independent rows/studies used for that group), the pooled mean difference (θ), and 95% confidence interval (CI) using DL and REML, $\tau^{2}$ between-study variance by DL and REML and Q-statistic and $I^{2}$ associated with heterogeneity. Finally, forest plots were generated for each group and estimator and saved as PNG and PDF files to ./outputs.

The script recalculates DL and REML pooled estimates (θ and 95% CI), $\tau^{2}$, Q, and $I^{2}$ for the T3 dataset with and without Hu (2021) [28], and the sensitivity results were saved to verification_table.xlsx or a separate sensitivity file.

## 3. Results

Initial identification: The PRISMA diagram reports the total records identified by each reviewer before duplicate removal. Reviewer 1 (Rv1) obtained 20 candidate papers, and Reviewer 2 (Rv2) obtained 35. These totals are the sum of records retrieved from each database per reviewer using the proposed query.

**Figure 1:**
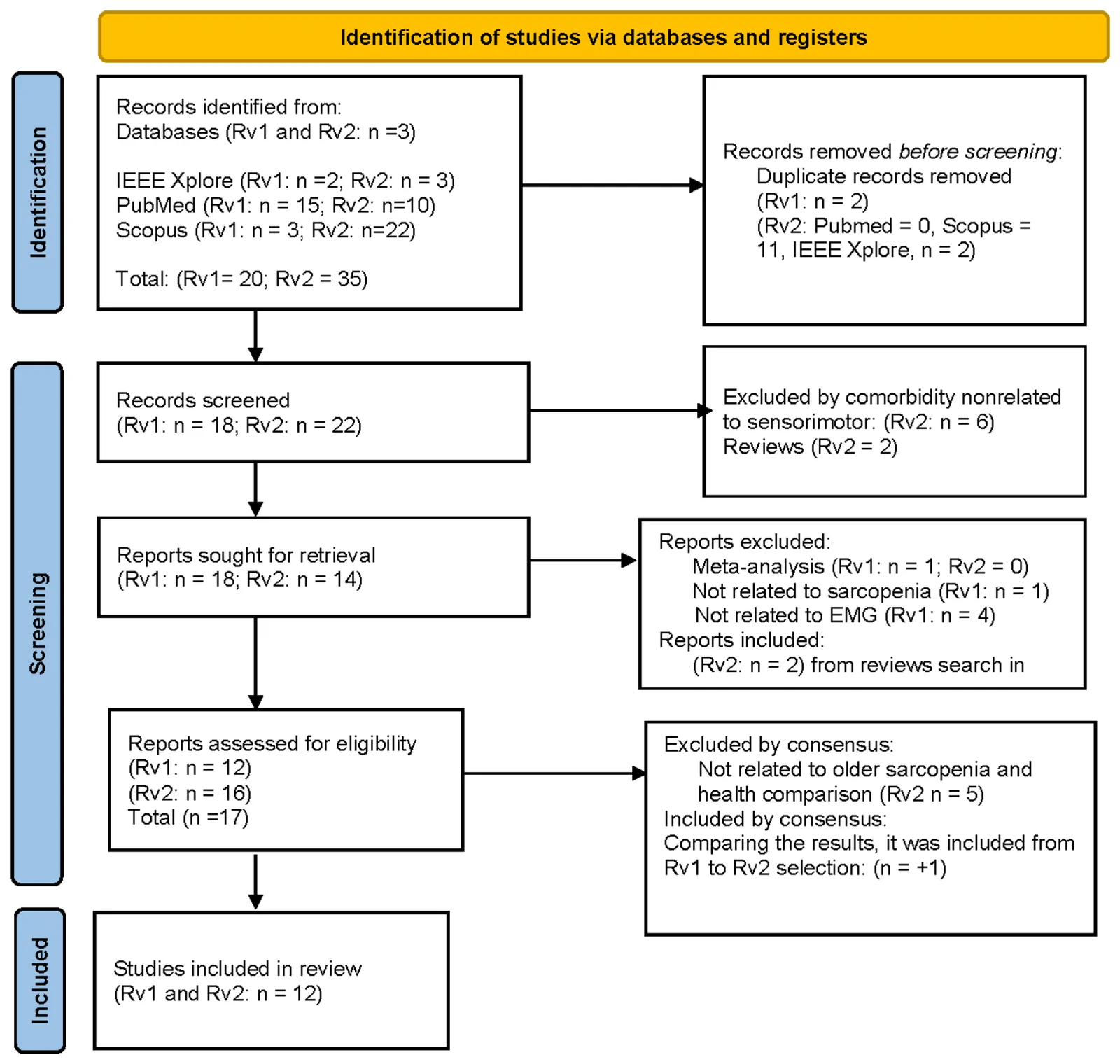
Flow diagram of the applied PRISMA process. Rv1 = reviewer 1; Rv2 = Reviewer 2. Ref. [29] Licensed under CC BY 4.0. To view a copy of this license, visit https://creativecommons.org/licenses/by/4.0/ accessed on 7 July 2026.

Duplicate removal: Two records were removed as duplicates in Rv1 before screening (Rv1 duplicates = 2). After duplicate removal, Rv1 proceeded to screening with 18 records. This duplicate removal step explains why the PRISMA identification box initially shows 20, while the screening box shows 18 for Rv1. Figure 1 shows a summary of the results.

Screening and reports assessed: For Rv2, the count after removing duplicates and initial exclusions resulted in 22 records screened, and later, 16 assessed for eligibility; for Rv1, the corresponding numbers were 18 screened and 12 assessed for eligibility. The combined number of reports assessed for eligibility (Rv1 + Rv2) is 17 distinct records (the sets overlap, and some records were identified by both reviewers).

Final included studies: Following independent screening and the consensus process (and after removing records excluded for comorbidity, unrelated EMG, meta-analysis, etc.), both reviewers converged on the same final set of included studies (n = 12). File S2 reports the reviewer-specific intermediate record sets (17 and 22) obtained automatically and used during the eligibility assessment; the PRISMA diagram depicts the overall identification and screening flow, where the initial totals include records prior to duplicate removal.

### 3.1. Comparison of Studies

According to the primary analytical function of the sEMG signal and the dominant physiological question addressed by each study, a set of analytical labels was established to guide the classification process, including activation, activation combined with coordination, posture-related activation, fatigue, activation combined with fatigue, ML-based performance (biomarker, classification, or diagnosis), neural control based on motor units, and coordination/symmetry. To facilitate interpretation of the comparative structure, Table 1, Table 2 and Table 3 summarize the objectives, methods, and main results of each article, along with the assigned analytical label.

Based on the segmented analysis approach, a comparative structure comprising four groups was established: Group A focused on functional and postural muscle activation; Group B centered on fatigue and fatigability; Group C was dedicated to machine learning (ML) applications using sEMG; and Group D was related to neural control, motor units, and coordination. The assignment of studies to each group was based on their primary analytical focus, as defined by the guiding research questions described in Section 2.5. Table 4 shows the relationship between the labels and their corresponding comparison group.

All tables summarize concepts implemented in the selected studies, such as sEMG signal amplification gain (G), sampling frequency (fs), and the implementation of filters, such as the bandpass filter (BP), among others.

#### 3.1.1. Group A

The studies included in Group A used electromyography to analyze muscle activation during functional or postural tasks. Although this group is not entirely homogeneous, it shares a clear thematic core: sarcopenia and aging alter the way in which the neuromuscular system organizes activation during functionally meaningful activities. Within this group, Marshall et al. focus on the relative activation of the quadriceps during activities of daily living and resistance exercises [32]; Cai et al., in addition to quantifying response amplitude, incorporate antagonist co-activation and sEMG variability [35]; and Sepúlveda-Loyola et al. combine postural balance variables, such as the Brief-BESTest and TUG, with muscle activity of the lower limbs, trunk, and respiratory muscles [30]. Although the participants in this study have a comorbidity, the main analysis is about sarcopenic versus non-sarcopenic conditions and not about COPD.

The main commonality among these studies is that sEMG is not interpreted as an isolated value, but rather as the expression of a neuromuscular strategy. Marshall et al. demonstrate that older adults require greater relative quadriceps activation to complete functional tasks, suggesting increased effort to maintain performance [32]. Cai et al. extend this finding by showing that, in sarcopenia, not only does the magnitude of activation change, but also its distribution, with greater reliance on proximal musculature, reduced distal involvement, and increased co-activation [35]. Sepúlveda-Loyola et al., in turn, show that when tasks demand postural stability, sarcopenia is associated with reduced activation of muscles relevant for balance and poorer overall postural control [30].

Taken together, these findings indicate that Group A does not simply describe “more” or “less” sEMG activity, but rather a functional reorganization of muscle activation. Table 5 shows the main characteristics of the data reported in the articles in Group A. These are studied muscles, study population, and database availability, while Table 6 shows the main characteristics of the feature extraction methods, evaluation metrics, and results.

All three studies agree that aging—and particularly, in sarcopenia—is associated with detectable changes in muscle activation and reduced functional performance, although they analyze these effects in different contexts. Sepúlveda-Loyola et al. evaluated patients with chronic obstructive pulmonary disease (COPD), with and without sarcopenia, during balance tasks and demonstrated differences in postural control, functional tests, and muscle activation of the lower limbs, trunk, and respiratory muscles [30]. Marshall et al. compared quadriceps muscle activity in healthy young and older adults, focusing exclusively on activities of daily living and resistance exercise [32]. Cai et al. contrasted older adults with and without sarcopenia during the 6-Minute Walk Test (6MWT) and the Five Times Sit-to-Stand Test (5STS), incorporating a more detailed phase-based analysis, as well as co-activation and sEMG variability [35].

Overall, Sepúlveda-Loyola et al. [30] and Cai H et al. [35] describe compensatory strategies associated with sarcopenia, whereas Marshall et al. [32] provide a reference point for understanding how relative muscle effort changes with aging, even in the absence of sarcopenia.

In terms of the features extracted from EMG as a metric of muscle activation, the three studies converge on a consistent neuromuscular signature associated with sarcopenia when assessed with surface EMG during functional and postural tasks. Two complementary normalization approaches are used: Sepúlveda--Loyola emphasizes RMS changes relative to a control posture (%Δ*μ*VRMS) to detect postural activation deficits [30]. Marshall uses absolute amplitudes and normalization to peak MVC (%EMG_MVC) to quantify relative effort across activities [32], and Cai combines %MVC amplitudes with co-activation and variability metrics during gait and sit-to-stand tests [35]. Together these metrics reveal: (1) a distal-to-proximal redistribution of activation (reduced TA/GA activation, increased VM/VL/RF and gluteal activation); (2) higher relative activation (%MVC or %ΔRMS) in older/sarcopenic individuals for comparable tasks (reflecting reduced neuromuscular reserve); (3) increased antagonist–agonist co-activation (higher CI), especially around knee musculature; and (4) task-dependent changes in variability (greater proximal variability during gait; reduced trial-to-trial variability—more rigid patterns—during repeated sit-to-stand).

These combined EMG findings map directly onto functional impairments reported in the papers (slower gait, poorer balance, longer 5STS times, and larger COP excursions). Mechanistically, reduced distal output undermines ankle strategy and push-off efficiency, forcing compensatory proximal recruitment and co-activation that raise energetic cost and reduce movement economy. The different metrics provide complementary information: RMS/MAV and iEMG capture instantaneous and cumulative activation magnitude, %MVC quantifies effort relative to capacity, CI indicates stabilization strategy and inefficiency, and MeanSD characterizes stability/adaptability of motor control (Table 7). Contribution to sarcopenia assessment:

(a) Sensitivity to functional deficits: EMG-derived metrics reveal neuromuscular impairments that are not captured by measures of muscle mass alone, enabling the detection of early functional decline (e.g., reduced distal activation and increased relative effort), which is pertinent to fall risk and mobility limitations.
(b) Mechanistic insight: Characteristic patterns—such as distal-to-proximal redistribution of activation and elevated antagonist–agonist co-activation—clarify observed performance deficits and identify specific intervention targets (for example, ankle plantarflexors/dorsiflexors and neuromuscular coordination).
(c) Objective monitoring: Normalized EMG measures (e.g.,%MVC or %ΔRMS) and integrated metrics (iEMG) offer objective, reproducible biomarkers suitable for tracking disease progression or recovery in response to interventions targeting strength, power, coordination, or via biofeedback.
(d) Task specificity: Distinct functional assessments (e.g., gait, 5STS, and balance tests) yield different EMG signatures; a multimodal testing approach enhances diagnostic sensitivity and permits more precisely prioritized rehabilitation planning.
(e) Rehabilitation guidance: Co-activation indices and variability metrics inform clinical decision making by indicating when interventions should prioritize reduction of maladaptive co-activation and restoration of distal strength/power and sensorimotor adaptability, rather than focusing exclusively on proximal strengthening.

#### 3.1.2. Group B

Group B comprises studies focused on neuromuscular fatigue and fatigability. Fatigue is a subjective sensation of reduced energy or increased perceived effort during or after a task. Its definition is multidimensional, encompassing perception and psychological impact, but it does not necessarily imply measurable physiological impairment. In contrast, fatigability refers to the magnitude of quantitative change in physiological or functional performance induced by a standardized task (load) [39].

In this context, Habenicht et al. and Ebenbichler et al. analyze spectral fatigue in lumbar extensors and propose these metrics as potential early biomarkers of sarcopenia-related alterations. Both studies use spectral variables derived from electromyographic signals to examine how muscle response changes during sustained or repetitive tasks [36,38]. In contrast, Chilwan et al. combine measures of muscle activity with intratask changes during a sustained mastication test to reflect functional fatigability [37]. The study by Ebenbichler et al. was included in this review even though the participants suffer from chronic low-back pain because they focus on fatigue as an early biomarker of lumbar muscle function at risk of sarcopenia.

The main contribution of Group B is to demonstrate that the temporal dimension of the electromyographic signal is essential for understanding sarcopenia and aging. A particularly relevant finding is that smaller changes in signal metrics do not necessarily indicate increased functionality. In back muscle studies, a less pronounced spectral slope is interpreted as reduced involvement of fast fatigable fibers and possible neuromuscular remodeling, rather than as a functional advantage. Conversely, in the mastication study, fatigability is expressed as reduced muscular performance and intratask changes, particularly in the submental muscles. Thus, Group B indicates that sarcopenia may manifest both as alterations in muscle metabolism, as inferred from spectral variables, and as impaired functional endurance during sustained tasks.

Table 8 presents the main characteristics of the data reported in the Group B studies, including the muscles studied, study population, and dataset availability. Table 9 summarizes the main feature extraction methods, evaluation metrics, and results.

As shown in Table 8 and Table 9, there are marked differences in muscle systems and tasks. In Habenicht et al. (2020), maximal lumbar strength was comparable between young and older adults; however, the IMDF-sEMG slope decreased more rapidly in younger individuals, suggesting that this variable can detect early functional alterations even in the absence of evident strength loss [36]. In the work of Chilwan et al. (2025), older adults showed reduced masseter muscle performance, whereas the submental region exhibited more limited changes, primarily associated with task-induced fatigue [37]. Meanwhile, Ebenbichler et al. (2020) found that even in the presence of chronic low-back pain, older adults exhibited lower MF-sEMG fatigue than younger individuals [38]. Additionally, sex-related differences were observed, particularly in older women, reinforcing the notion of age-specific neuromuscular and metabolic changes. Taken together, Habenicht et al. (2020) [36] and Ebenbichler et al. (2020) [38] agree that spectral sEMG variables are more sensitive than maximal strength for detecting early muscle aging, whereas Chilwan et al. (2025) [37] show that, in masticatory muscles, aging is more strongly reflected as reduced functional performance and endurance during prolonged tasks.

Although the three studies employed different EMG-derived variables, they collectively demonstrate that age-related changes in neuromuscular activation can be quantified using either amplitude-based or frequency-based EMG metrics. Table 10 and Table 11 summarize the features and metrics used to assess fatigue and fatigability.

Chilwan et al. (2025) [37] focused primarily on amplitude-derived parameters, including mean average amplitude, peak amplitude, EMG power, and maximum voluntary contraction (MVC), during the Six-Minute Mastication Test. These variables were used to quantify reductions in muscle activation capacity throughout the task. Significant decreases in mean amplitude and EMG power over time, together with lower peak amplitudes in older participants, indicate progressive fatigue of the masticatory muscles and reduced functional muscle performance. These findings suggest that amplitude-based EMG measures are particularly suitable for evaluating functional muscle fatigue during prolonged dynamic tasks [37].

In contrast, Habenicht et al. (2020) and Ebenbichler et al. (2020) [36,38] emphasized frequency-domain analysis, specifically Instantaneous Median Frequency (IMDF) and its temporal slope. Both studies demonstrated that IMDF slope is considerably more sensitive than RMS amplitude for detecting age-related differences in neuromuscular fatigue. During sustained cyclic back-extension exercises, younger adults exhibited a steeper decline in median frequency than older adults, indicating greater fatigability. Importantly, the authors interpreted the smaller decline observed in older adults not as superior fatigue resistance but as evidence of altered neuromuscular physiology resulting from aging [36,38].

This interpretation is consistent with the current understanding of sarcopenia. Age-related loss of motor neurons, preferential atrophy of fast-twitch (Type II) muscle fibers, motor unit remodeling, and reduced muscle fiber conduction velocity modify the spectral characteristics of the EMG signal. Consequently, older adults frequently exhibit smaller decreases in median frequency despite impaired muscle quality and reduced functional reserve. Therefore, a reduced IMDF or MF slope should not be interpreted as improved fatigue resistance but rather as a consequence of diminished recruitment of high-threshold motor units. Similarly, amplitude-derived variables may also be affected by sarcopenia. Reduced mean amplitude, peak amplitude, and EMG power reflect impaired motor unit recruitment and decreased capacity to sustain muscular activation during prolonged activity. These alterations are compatible with the reduction in muscle mass and muscle quality that characterize sarcopenia. However, RMS amplitude appears to be less sensitive to aging-related adaptations because it is influenced by several physiological and methodological factors, including amplitude cancellation, motor unit synchronization, and changes in motor unit action potential morphology.

#### 3.1.3. Group C

Group C represents the most translational line of the review, as it integrates studies in which sEMG is no longer used solely as a descriptive tool but, instead, serves as input to machine learning systems for classification, screening, or functional estimation. In this group, the main objective was to enhance the use of technological resources in sEMG analysis, so the group is heterogeneous with respect to the clinical objective. Song et al. developed a regression model to estimate a continuous index of muscle function [8]. Kumar et al. trained binary classifiers to distinguish between control subjects and individuals at risk of sarcopenia during functional tasks [9]. Li et al. employed explicit clinical criteria AWGS 2019 and reported typical screening metrics, including sensitivity, specificity, and AUC [33].

The main similarity across these studies is both technological and functional: all are based on the premise that sEMG contains sufficient information to infer clinically relevant muscle states. However, they should not be compared as if they were evaluating the same construction. Song et al. focus on regression and agreement [8]; Kumar et al. on functional risk classification [9]; and Li et al. on a classification approach closer to a clinical screening context [33].

Group C demonstrates that sEMG can be transformed into automated tools with clinical potential. At the same time, it highlights an important limitation: model performance depends critically on the ground truth used, the type of task, the feature extraction strategy, and the validation approach used by the systems.

Table 12 presents the main characteristics of the data reported in the Group C studies, including the muscles studied, study populations, and dataset availability. Table 13 and Table 14 summarize the main feature extraction methods, evaluation metrics, and results.

As shown in Table 13 and Table 14, the studies differ substantially in their objectives, populations, and experimental designs. Song et al. (2024) proposed a digital biomarker of muscle function based on electrically induced signals recorded from the rectus femoris in 98 healthy adults aged 20 to 60 years [8]. Their primary aim was not to diagnose clinical sarcopenia, but to estimate the muscle strength-to-muscle mass ratio using a wearable system with a regression model based on MCP + CWT + CNN/MLP. In contrast, Kumar et al. (2024) [9] and Li et al. (2024) [33] explicitly addressed classification tasks for sarcopenia risk or diagnosis. Kumar et al. analyzed 93 adults aged 20 to 59 years during walking and squat tasks, recording lower-limb muscles (RF, BF, TA, GA) and combining EMD + mRMR + machine learning classifiers [9] Meanwhile, Li et al. studied community-dwelling older adults during a hand grip task, recording six forearm muscles, adopting a more clinical and population-based approach grounded in AWGS 2019 criteria and using an ensemble classifier composed of SVM + RF + GBM [33].

They also differ in signal type and achieved performance. Song et al. (2024) worked with stimulated muscle contraction signals (SMCSs), i.e., signals elicited via electrical stimulation to reduce the influence of voluntary control, achieving the highest correlation with the reference biomarker (r = 0.89) [8]. Kumar et al. (2024) used voluntary sEMG during functional activities and showed that incorporating EMD improved performance by approximately 4–5%, achieving accuracies of 0.88 for normal walking, 0.89 for fast walking, 0.81 for standard squat, and 0.80 for wide squat with an MLP [9]. In contrast, Li et al. (2024) reported more moderate performance in real-world community diagnosis, with accuracy = 0.73 and sensitivity = 0.79, using their voting classifier [33]. However, they contributed greater interpretability through SHAP analysis, identifying waveform length (WL) and CWT kurtosis as the most influential features.

Overall, Song et al. focus more on quantifying muscle function, Kumar et al. on sarcopenia risk in younger and middle-aged adults [8], and Li et al. on practical screening in older populations [33]. Therefore, the three studies should be considered complementary rather than comparable. All of them found automatic identification a viable strategy for evaluating muscular motor performance.

The reviewed studies demonstrate two complementary paradigms for applying machine learning to sEMG analysis. While Kumar et al. (2024) [9] and Li et al. (2024) [33] approached sarcopenia assessment as a classification problem, distinguishing individuals with and without sarcopenia, Song et al. (2024) [8] proposed a regression-based framework for estimating a continuous digital biomarker of muscle function. Together, these studies illustrate the evolution of artificial intelligence from categorical diagnosis toward quantitative functional assessment, highlighting the increasing role of machine learning in supporting both clinical decision making and longitudinal monitoring of muscle health. One of the most consistent findings across the reviewed literature is that feature engineering remains a critical determinant of model performance. Traditional amplitude-based descriptors, including root mean square (RMS), mean absolute value (MAV), and integrated EMG (iEMG), continue to provide valuable information regarding motor unit recruitment and muscle activation. However, recent studies have increasingly incorporated multidomain feature extraction by combining temporal, spectral, nonlinear, and time-frequency characteristics. Features such as waveform length (WL), median and mean frequencies (MDF and MNF), peak frequency (PKF), sample entropy, and continuous wavelet transform (CWT)-derived descriptors were consistently identified as highly informative predictors of muscle function and sarcopenia (Table 15 and Table 16). These variables capture complementary physiological mechanisms, including motor unit recruitment, muscle fiber conduction velocity, neuromuscular synchronization, and signal complexity, all of which are progressively altered during aging and sarcopenia.

Feature selection strategies also contributed substantially to improving model performance. Kumar et al. demonstrated that combining empirical mode decomposition (EMD) with minimum redundancy maximum relevance (mRMR) feature selection enhanced classification accuracy by reducing redundancy among hundreds of extracted EMG variables while preserving physiologically relevant information.

An important methodological advancement introduced by Song et al. (2024) is the transition from conventional handcrafted feature-based machine learning toward hybrid deep-learning architectures [8]. Rather than relying exclusively on manually engineered descriptors, their framework combines muscle contraction pattern (MCP) features, continuous wavelet transform (CWT) images, and demographic information within a convolutional neural network (CNN) followed by a multilayer perceptron (MLP) regression model. Unlike traditional pipelines, in which feature extraction precedes classification, CNN automatically learns hierarchical representations directly from time-frequency images while preserving physiologically meaningful handcrafted features (Table 17).

Another significant advancement is the incorporation of feature selection and explainability techniques, such as minimum redundancy maximum relevance (mRMR) and SHapley Additive exPlanations (SHAP), which improve both predictive performance and clinical interpretability (Table 18). These approaches enhance confidence in machine learning predictions by identifying the physiological relevance of individual EMG features, thereby facilitating their translation into clinical practice. The metrics to evaluate ML models applied in the reviewed papers are shown in Table 19.

From a physiological perspective, the reviewed machine learning models consistently relied on EMG-derived features that reflect fundamental mechanisms underlying sarcopenia. Age-related motor neuron loss, preferential atrophy of fast-twitch (Type II) muscle fibers, reduced muscle fiber conduction velocity, impaired motor unit recruitment, and decreased neuromuscular synchronization collectively alter both the amplitude and spectral characteristics of the EMG signal. Consequently, the extracted EMG features represent functional manifestations of neuromuscular deterioration rather than isolated mathematical descriptors. The incorporation of these physiologically meaningful biomarkers into machine learning models improves not only predictive performance but also the biological plausibility of the resulting algorithms.

A consistent finding across the literature is the growing importance of multidomain EMG feature extraction, integrating amplitude, frequency, nonlinear, and time-frequency-based descriptors to comprehensively characterize neuromuscular function. Features such as waveform length, median and mean frequencies, peak frequency, sample entropy, and continuous wavelet transform-derived parameters repeatedly emerged as robust biomarkers reflecting motor unit recruitment, muscle fiber conduction velocity, signal complexity, and neuromuscular coordination. These physiological alterations closely correspond to the mechanisms underlying sarcopenia, including motor neuron loss, preferential Type II fiber atrophy, impaired recruitment, and reduced neuromuscular synchronization.

Importantly, the work of Song et al. expands this paradigm by introducing a regression-based framework for estimating a continuous digital biomarker of muscle function rather than performing binary classification alone [8]. This transition from diagnostic classification to quantitative functional assessment represents an important step toward personalized and longitudinal monitoring of muscle health.

Table 20 and Table 21 summarize the characteristics of the ML methods used on each of the papers analyzed in Group C. As it can be observed, the size of the databases, the balance, preprocessing techniques, splitting strategies and evaluation metrics differ for the three of them.

#### 3.1.4. Group D

Group D brings together studies focused on neural control of motor units and intermuscular coordination, that is, research aimed at understanding how aging and sarcopenia modify the functional organization of the neuromuscular system beyond mere loss of muscle mass. Within this group, Hu et al. [28] and Dias et al. [34] analyze variables such as firing rate, motor unit action potential (MUAP) amplitude, recruitment threshold, and the relationship between neural discharge and force production. In contrast, He et al. examine the spatial distribution of sEMG activity across multiple muscles and propose an index to quantify the symmetry of activation patterns as a biomarker [31].

The relevance of this group lies in providing a physiological framework to explain why muscle performance changes with aging or sarcopenia. Collectively, these studies suggest that impairment depends not only on the amount of available muscle but also on how motor units are recruited, modulated, and coordinated. However, the observed changes do not follow a single direction and should not be interpreted in an oversimplified manner.

Table 22 presents the main characteristics of the data reported in the Group D studies, including the muscles studied, populations, and dataset availability. Table 23 and Table 24 summarize the main features: extraction methods, evaluation metrics, and results.

All three studies agree that ageing or sarcopenia are expressed in the sEMG signal as a form of neuromuscular reorganization, although they examine different levels of the motor system. He et al. (2024) investigated multi-muscle coordination of the upper limb in older adults with and without sarcopenia, using six forearm muscles during hand grip tasks at 20% and 50% MVC, and they proposed using the ICDMC index to quantify the symmetry of spatial sEMG activation patterns [31]. In contrast, Dias et al. (2019) and Hu et al. (2021) focused on motor unit properties [28,34]. Thus, He et al. approach a potential community screening tool by analyzing altered intermuscular coordination, whereas Dias et al. and Hu et al. provide deeper insight into neural control mechanisms and motor unit adaptation.

After analyzing the reported data, all results are consistent with a positive difference between the control and sarcopenia conditions on the Grip Strength Test (6/12), but similar task conditions are rare across the different muscles. Almost all the studies considered had small sample sizes, ranging from five per group to hundreds of participants, as reported by Ebenbichler, G. et al. (2020) [38].

Table 25 and Table 26 summarize EMG features reported across three studies (He et al. 2024; Dias et al. 2019; Hu et al. 2021), providing interpretations of each feature in relation to muscle control and sarcopenia [28,31,34]. Time-domain amplitude metrics, such as RMS and MAV/IEMG and wavelength (WL), are associated with overall muscle activation and spectral content, respectively. Notably, He et al. introduced a novel multichannel symmetry index (ICDMC) derived from combined time-domain features. Additionally, Dias et al. and Hu et al. reported motor unit (MU) measures from decomposed EMG signals, including mean firing rate (MFR), MUAP amplitude, and firing rate per unit force, to elucidate the relationship among neural drive, compensatory remodeling, and age-related muscle changes. Although frequency metrics (MDF/MPF) were extracted, they were not consistently discriminative in the multichannel study.

### 3.2. Risk of Bias Assessment

**Figure 2:**
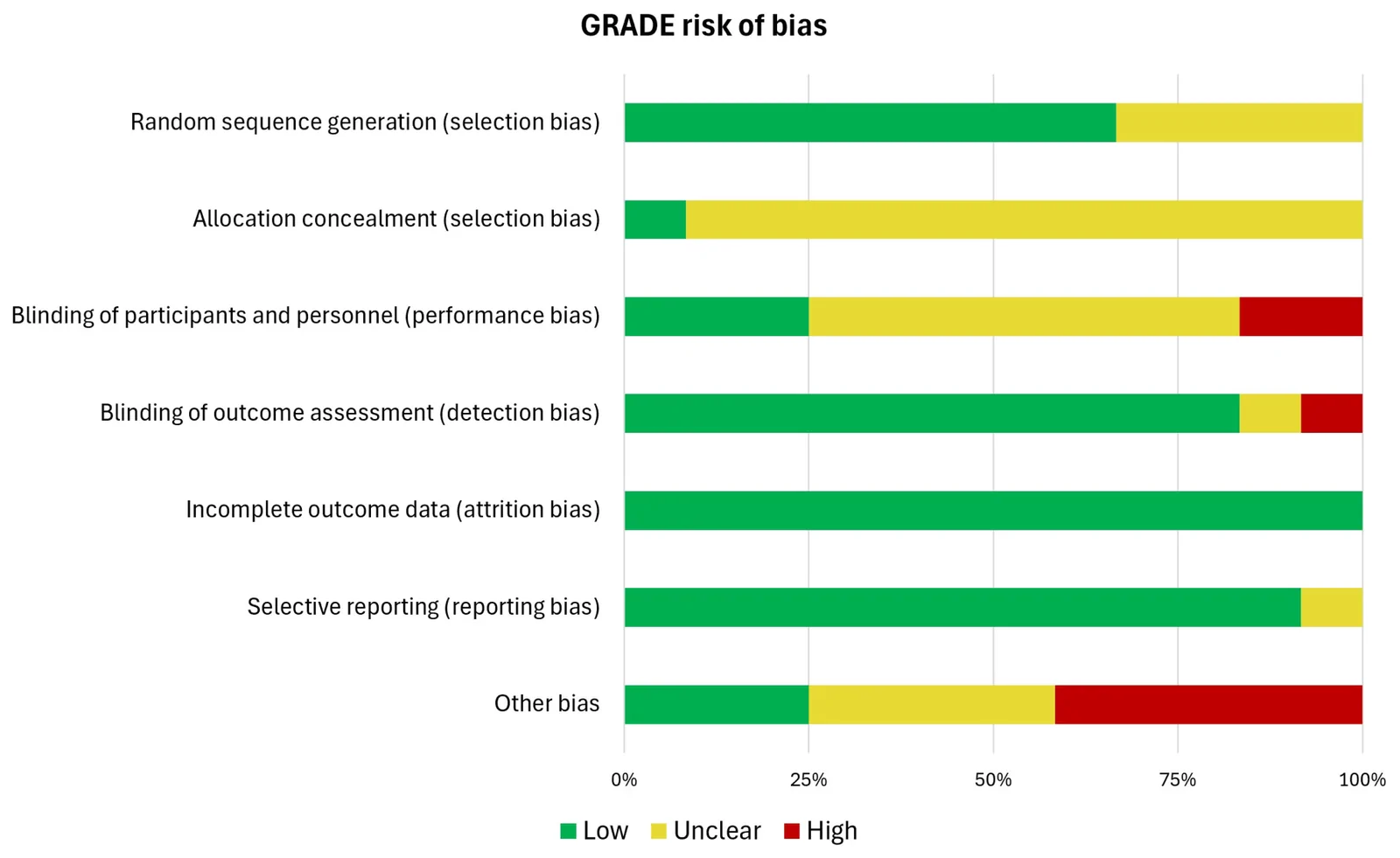
Overall risk of the selected studies.

**Figure 3:**
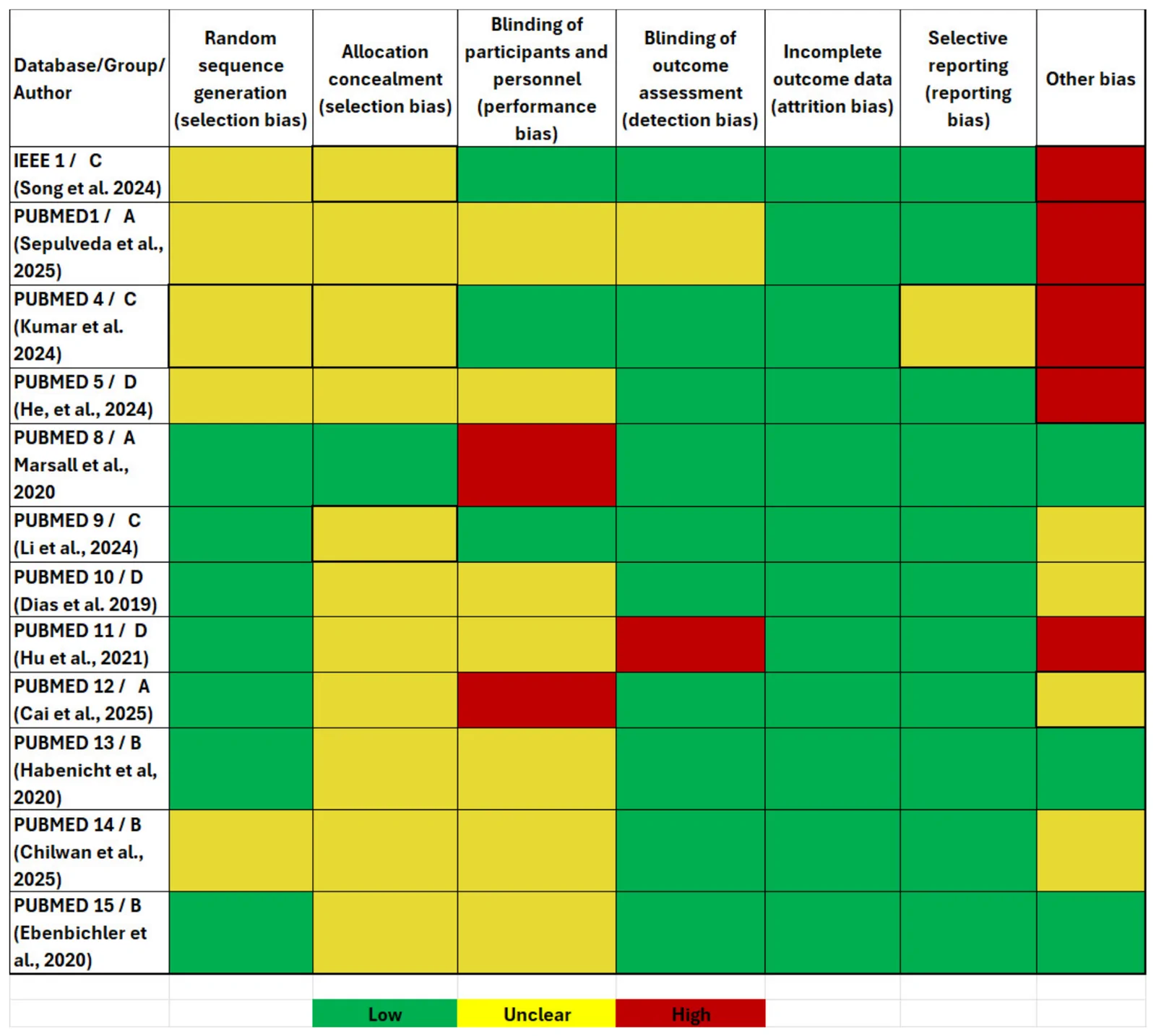
GRADE risk of bias evaluation per article [14]. Letters A, B, C, and D refer to the group of study [8,9,28,30,31,32,33,34,35,36,37,38].

The methodological quality of the included studies was assessed using the GRADE Risk of Bias framework to support the review’s findings. Each study was classified as low, unclear, or high risk of bias based on reported methods. The reviewed studies demonstrated acceptable methodological quality, particularly with respect to incomplete outcome data and selective reporting, both of which were consistently low risk. The blinding of outcome assessment also yielded mostly low-risk results, suggesting that objective sEMG data analysis was used. Potential limitations are outlined in Figure 2 and Figure 3 for the overall evaluation and per article, respectively.

However, several methodological limitations were identified, primarily related to selection and performance bias. Allocation concealment and randomization were often poorly described, resulting in numerous unclear risk assessments. The greatest concern was the blinding of participants and personnel, which was often unclear or high risk due to challenges with full blinding in sEMG protocols.

The “other bias” category also revealed methodological heterogeneity across the reviewed literature. The relatively high proportion of unclear and high-risk assessments is associated with factors such as limited sample sizes, inconsistent sEMG acquisition protocols, variability in hardware configurations, and differences in preprocessing methodologies. These issues remain common challenges within sEMG research and contribute to reduced comparability between studies. Despite generally satisfactory reporting quality in sEMG studies, improving methodological transparency and bias-control strategies is essential to increase reproducibility and reliability.

Following a systematic reconciliation between the original GRADE justifications in File S3 and Table S3.1 and the independent RoB appraisals summarized in Figure 3, we applied targeted adjustments to several domain-level ratings and to the overall RoB of three articles. This reconciliation used tool-specific drivers (PROBAST, AXIS, COSMIN, and QUADAS-2) to identify where Table S3.1 had under- or overestimated the risk, particularly for ML/model development and manual processing studies.

Three studies had notable upward revisions in overall RoB after the cross-check: (Kumar et al. 2024) and (Hu et al., 2021) were reclassified to High overall RoB, and (He et al., 2024) was revised to Some concerns/High, depending on detection-bias impact on primary outcomes [9,28,31]. These changes were driven by unclear or nonstandard ground-truth labelling, evidence of possible feature-selection/validation leakage, and extensive manual outcome processing without reported inter-rater reliability.

Three further studies received domain-level upward adjustments: (Song et al. 2024 [8]), (Li et al., 2024 [33]), and (Cai et al., 2025 [35]) were assigned higher concern levels in specific domains (notably *Other bias*, *Allocation/Applicability*, and *Randomisation*), reflecting issues such as model overfitting risk, lack of nested feature-selection in cross-validation, and limited external generalizability. The most frequent drivers for upward adjustments were: unclear or inadequately described outcome/labelling procedures (risking misclassification bias); absence of external validation sets (raising concerns about overfitting and optimism); non-nested feature-selection or tuning steps (risking data leakage in predictive models); and reliance on manual decomposition or annotation without inter-rater reliability metrics (increasing detection bias).

Quantitatively, after integrating the table shown in Table S3.3, the distribution of overall GRADE RoB across the 12 studies shifted modestly toward higher concern: the number of studies rated Low overall RoB decreased, the number with Some concerns increased, and the count of High RoB studies increased by two (Kumar and Hu). These shifts are reflected in Figure 2 (overall bar/stack counts) and Figure 3 (per-article domain flags).

These upward adjustments primarily affect the interpretation of Group C, predictive/model development, and any findings that rely on manual signal processing for primary outcome derivation. Studies reclassified to Higher RoB should be interpreted with caution regarding model generalizability and clinical applicability.

Measurement-focused studies that reported reliability metrics and explicit acquisition/processing protocols largely retained Low/Some concerns ratings for measurement domains, but several received explicit “Other bias/Applicability” flags where small sample sizes or single-center recruitment limited generalizability.

For transparency, the updated per-article entries in Figure 3 include a bold border reporting a change, summarized in File S3, Table S3.4, drivers that motivated any rating changes (e.g., “non-nested feature selection detected; no external validation”, “manual decomposition w/out inter-rater reliability”). These footnotes allow readers to trace domain-level decisions and to assess how each study’s methodological limitation maps to the global RoB.

### 3.3. Hand Grip-Based Clinical Reference for sEMG Assessment

Upon analysis of the 12 articles, only 6 out of 12 (50%) articles use hand grip MVC as a clinical reference for sEMG assessment.

A quantitative clinical context commonly used in clinical screening of sarcopenia is the hand grip test. It is useful for interpreting surface electromyography (sEMG). A random-effects meta-analysis was conducted on studies comparing hand grip-related measures in older adults with and without sarcopenia. Three prespecified independent subgroups were analyzed separately: elderly females (k = 2), elderly males (k = 2), and the overall elderly subgroup (k = 4). No overall pooled estimate was calculated across these subgroups because some studies contributed data to more than one subgroup, which would result in non-independent reuse of study estimates. For the overall elderly subgroup, the pooled mean difference was 6.95 (95% CI: 4.77–9.13) using REML, with moderate between-study heterogeneity ($I^{2}$ = 41.8%). These findings support the presence of a consistent group-level difference in hand grip-related performance between older adults with and without sarcopenia, providing a clinically interpretable reference against which multimodal sEMG-derived neuromuscular features can be contextualized.

Accordingly, the results presented in File S4 for hand grip performance may serve as a clinically interpretable functional reference, while sEMG provides complementary information regarding the neuromuscular mechanisms underlying force generation and fatigue. The combination of these modalities may, therefore, provide a more comprehensive characterization of sarcopenia than either functional performance or electrophysiological measures considered independently.

Collectively, these findings indicate that sarcopenia is consistently associated with significant alterations in the studied outcome, with the most reproducible evidence observed in elderly men. The greater heterogeneity among studies involving women underscores the need for further research addressing sex-specific biological and methodological factors that may influence the magnitude of this association.

In all comparisons, the pooled effects indicate consistently higher hand grip strength in the control groups relative to individuals with sarcopenia, supporting the established association between sarcopenia and reduced muscular function.

In Table 27, which represents the overall comparison between control and sarcopenic populations, all included studies reported positive mean differences in favour of the control groups. Although some heterogeneity is evident, particularly in the study by Hu (2021), the pooled effect shows a clear reduction in hand grip strength among sarcopenic individuals [28]. The confidence intervals of most studies do not cross zero, indicating statistically meaningful differences between groups. Further details of the analysis can be found in File S4 [40,41].

## 4. Discussion

The integration of the four groups supports the notion that sarcopenia should not be understood solely as a reduction in muscle mass or strength, but rather as a process of neuromuscular reorganization. Group A shows how this reorganization is expressed during functional and postural tasks; Group B demonstrates that it is also reflected in the temporal evolution of the signal through fatigue and fatigability; Group C provides evidence that these alterations leave a sufficiently consistent signature to generate computational models; and Group D contributes the physiological basis that helps explain why activation patterns, variability, co-activation, and classification performance change. Taken together, these groups do not represent isolated blocks, but rather complementary levels of observation of the same biological phenomenon.

One of the most consistent patterns across groups is the presence of neuromuscular compensation mechanisms. In Group A, compensation appears as relative overactivation or changes in postural balance. In Group B, it emerges as atypical fatigue profiles, suggesting alterations in muscle composition and recruitment strategies. In Group C, compensation is detected indirectly, as these very adaptations enable models to distinguish between subjects or functional states. Finally, in Group D, compensation is identified more directly through motor unit parameters, firing rates, and patterns of muscular organization. This reinforces the idea that sarcopenia is a dynamic alteration of the neuromuscular system rather than merely a structural phenomenon.

Nevertheless, the discussion must also acknowledge clear limitations in comparability. The groups differ in their levels of analysis, the nature of their metrics, the experimental tasks, the muscles studied, and the clinical robustness of the reference standards. It is not equivalent to compare normalized sEMG amplitude, co-activation indices, spectral slopes, firing rates, motor unit metrics, or classifier accuracy, as each addresses a different research question. The heterogeneity in study designs and outcomes prevented the application of quantitative meta-analysis, limiting the ability to derive pooled estimates. Furthermore, the search was restricted to selected databases and may not have captured all relevant studies, particularly those not publicly available, so the search for universal biomarkers is contingent on the availability of electromyographic data. Of the 12 studies analysed, only four explicitly reported that their sEMG datasets could be made available upon request, while the remaining studies did not indicate either open data availability or the possibility of data access. This lack of transparency and accessibility limits reproducibility, hinders cross-study validation, and constrains the development and external validation of data-driven approaches, particularly in machine learning. Consequently, the lack of sufficiently large, standardized, and accessible sEMG databases remains a significant barrier to the development of robust, generalizable biomarkers for sarcopenia.

An important aspect of this discussion is the comparison between the available secondary literature and the specific contribution of the present review, allowing the identification of whether the findings are congruent with, complementary to, or divergent from previous systematic reviews. A complementary search, filtered to include systematic reviews and meta-analyses, identified relevant studies. On one hand, reviews with explicit systematic methodology, eligibility criteria, and replicable procedures were identified, such as those by James et al. (2021) and Labata-Lezaun et al. (2020) [42,43]. The former focused on the influence of resistance training on neuromuscular function in middle-aged and older adults, showing effects on voluntary activation but not on sEMG activity during MVC or antagonist co-activation; however, the predominance of healthy older adult populations in the included studies may limit the direct applicability of these findings to sarcopenic or clinical populations. The latter evaluated protein supplementation combined with resistance training and found that, in healthy older adults, there were no clear additional benefits in muscle strength or physical performance compared with resistance training alone or with a placebo.

The results of the studies by Piasecki et al. (2016) and Birkbeck et al. (2021) are valuable for understanding the physiological basis of neuromuscular aging and serve as a relevant precedent for a systematic strategy to compare studies on electromyography and sarcopenia [44,45]. Piasecki et al. (2016) described that healthy aging is associated with an approximate 40% reduction in the number of motor units and an enlargement of the surviving units, providing a solid foundation for understanding neural remodeling with aging [44]. In turn, Birkbeck et al. (2021) reviewed the potential of Motor Unit Magnetic Resonance Imaging (MUMRI) as a non-invasive tool for studying motor unit activity, highlighting both the limitations of conventional electromyography and the value of emerging approaches for investigating sarcopenia and muscle aging [45]. This technique may evolve into a gold standard method in clinical practice, but at least with current technology, it is unlikely to be useful for the early identification of sarcopenia when used as a screening approach. In this context, the main contribution of the present review is the organization of the primary literature on sEMG and sarcopenia into an analytical framework based on the function of the signal and the dominant physiological question. This review demonstrates how sEMG interpretation varies across levels of analysis: functional activation, fatigue, computational modeling, and neural control. It also highlights that, although there is substantial electromyographic evidence in sarcopenia, there is still insufficient standardization to propose a single, universal biomarker, even though important efforts toward standardization have been made, particularly in Asia through the consolidation of the AWGS 2019 criteria in 2022, which is proposed to provide updated diagnostic thresholds and a harmonized framework for identifying sarcopenia in clinical and research settings [46]. In this sense, the review integrates and structures a field that has so far been addressed in a fragmented manner.

An additional limitation identified in this review is the limited availability of electromyographic data in the search for universal biomarkers. Of the 12 studies analyzed, only four explicitly reported that their sEMG datasets could be made available upon request, while the remaining studies did not indicate either open data availability or the possibility of data access. This lack of transparency and accessibility limits reproducibility, hinders cross-study validation, and constrains the development and external validation of data-driven approaches, particularly in machine learning. Consequently, the lack of sufficiently large, standardized, and accessible sEMG databases remains a significant barrier to the development of robust, generalizable biomarkers for sarcopenia.

Overall, the meta-analytic findings consistently demonstrate that hand grip strength is significantly reduced in sarcopenic individuals irrespective of sex, reinforcing its clinical relevance as a functional biomarker for the identification and assessment of sarcopenia.

Our crosswalk between structured RoB tools and GRADE judgments (expanded in File S3) revealed that most studies were concordant across instruments, but that ML/predictive-modeling studies and those with substantial manual outcome processing required targeted adjustments. By explicitly documenting these domain-level issues (e.g., ground-truth clarity, nesting of feature selection within cross-validation, and external validation) and reflecting them in Figure 2 and Figure 3, we aim to provide a more accurate appraisal of study-level certainty and to guide future replication efforts.

From a clinical perspective, these findings highlight the potential of sEMG as a complementary tool for the functional assessment of sarcopenia, particularly in early detection. For research, there is a clear need for standardized acquisition protocols, feature extraction methods, and open-access datasets to enable reproducibility and comparability across studies. Future work should focus on validating robust biomarkers and integrating multimodal approaches to improve assessment accuracy.

## 5. Conclusions

The evidence reviewed indicates that sarcopenia is a multifactorial process of neuromuscular reorganization for which surface electromyography (sEMG) provides a valuable observational window. An analytical approach that grouped studies by primary research question and synthesized findings within and across groups yielded several key conclusions. Changes in muscle activation are evident during functional and postural tasks; fatigue and fatigability are altered; and sEMG signals can be used as tools for classification, screening, and functional estimation; these observations have a physiological basis in motor unit behavior, intermuscular coordination, and neural control. sEMG-derived features, such as normalized RMS/%MVC, integrated activity, co-activation indices, and variability metrics, provide a multidimensional and functionally meaningful assessment of sarcopenia. Across studies, a reproducible pattern is observed: reduced distal activation, increased proximal recruitment and co-activation, and altered motor variability, which link diminished neuromuscular capacity to impaired balance, gait, and functional performance. Incorporating these complex sEMG measures into assessment protocols enhances sensitivity to functional decline, informs targeted rehabilitation strategies (including ankle strengthening, coordination training, and reduction of maladaptive co-activation), and provides objective outcomes for monitoring treatment efficacy. Overall, sEMG complements structural measures of muscle mass and strength and should be considered a valuable component of comprehensive sarcopenia assessment and personalized intervention planning.

The evidence supports a two-tier EMG strategy for sarcopenia assessment. The first tier involves multichannel time-domain features (RMS, MAV, WL, SSC, ZC) and derived symmetry indices (such as ICDMC), which enable non-invasive, low-effort community screening of altered coordination and muscle composition. The second tier utilizes motor unit metrics, including mean firing rate, firing rate per unit force, and MUAP amplitude, obtained from decomposed or high-density EMG, to characterize neural compensation and structural remodeling. Symmetry indices and related multichannel measures are promising for screening at low contraction intensities, while motor unit metrics serve as mechanistic biomarkers: decreased mean firing rate suggests reduced central drive; increased MUAP amplitude may indicate reinnervation or changes in innervation ratio; and altered firing rate per unit force indicates early compensatory neural effort.

The integration of sEMG with machine learning presents substantial potential as a digital health technology for sarcopenia assessment. In addition to improved diagnostic accuracy, these approaches provide objective, non-invasive, and physiologically meaningful biomarkers that support early screening, disease monitoring, rehabilitation assessment, and treatment follow-up.

Despite these advances, important limitations persist. Considerable heterogeneity across studies—including electrode placement, acquisition protocols, contraction tasks, preprocessing, feature extraction, and validation strategies—continues to restrict direct comparison and reduce model generalizability. Furthermore, many investigations rely on relatively small datasets and lack external validation, highlighting the need for standardized acquisition protocols and multicenter collaborative studies.

Collectively, the findings demonstrate that sEMG not only characterizes muscular deterioration but also elucidates how the neuromuscular system compensates, redistributes workload, and ultimately loses efficiency in sarcopenia. Given the widespread use of hand grip strength as a standard functional measure in multicenter studies and its inclusion in operational definitions such as AWGS and EWGSOP, adopting a standardized hand grip protocol across studies would facilitate quantitative comparisons and enable the creation of larger, harmonized electromyographic datasets for biomarker discovery.

This review provides an integrative perspective that organizes the existing literature and demonstrates that the utility of sEMG extends beyond a single metric. Its strength lies in the capacity to interrogate the condition at multiple levels: functional, temporal, computational, and neurophysiological. This reinforces the role of electromyography as a complementary tool for characterization, monitoring, and mechanistic understanding of sarcopenia in older adults. While methodological reporting and outcome management in contemporary sEMG studies are often acceptable, recurrent deficiencies persist, including a lack of experimental blinding, incomplete allocation procedures, and insufficient methodological transparency.

Future research should: (a) integrate multichannel and motor unit features in larger, longitudinal cohorts; (b) control for confounders such as adiposity, sex, and age; (c) standardize acquisition, normalization, and labeling protocols; and (d) evaluate predictive validity for functional outcomes and treatment response. Additionally, emphasis should be placed on developing standardized, explainable artificial intelligence frameworks, integrating multimodal physiological data, and conducting large-scale prospective validation to expedite clinical translation of AI-assisted neuromuscular assessment and establish robust digital biomarkers for sarcopenia.

Future studies should adopt standardized reporting frameworks, provide clearer protocol descriptions, and implement stronger bias-control strategies to enhance reproducibility, comparability, and overall scientific reliability.

## Figures and Tables

**Table 1:** Comparison of studies, including the objective of the study, methods, and results (Part I).

Title and Analytical Label	Authors, Year	Objective, Methods, and Results
[8] Digital Biomarker for Muscle Function Assessment Using Surface Electromyography with Electrical Stimulation and a Non-Invasive Wearable Device **ML/biomarker**	K. Song; H. Eun Shin; W. Park; D. Lee; J. Jang; G. Yang Shim; S. Choi; M. Kim; H. Lee; C. Won Won (2024)	Objective: To develop a digital biomarker of muscle function from sEMG recorded during electrically induced contraction (stimulated muscle contraction signals, SMCS) using a wearable device. Methods: sEMG from the rectus femoris, extraction of muscle contraction pattern (MCP) features (spectrogram, envelope, autocorrelation, and slope), transformation using continuous wavelet transform (CWT), convolutional neural network (CNN), and regression with a multilayer perceptron (MLP) to estimate a muscle quality index (strength/mass). Results: The best model (MCP + CWT/CNN + body variables) achieved r = 0.89, ME = −0.06, and STDE = 0.68 compared with the reference value.
[30] Are there differences in postural control and muscular activity in individuals with COPD and with and without sarcopenia? **Posture + activation**	Sepúlveda-Loyola W, Álvarez-Bustos A, Va-lenzuela-Fuenzalida JJ, Ordinola Ramírez CM, Saldías Solis C, Probst VS. (2025)	Objective: To compare postural control and sEMG activity between peo-ple with chronic obstructive pulmonary disease (COPD) with and without sarcopenia. Methods: 35 patients with COPD, classified according to the European Working Group on Sarcopenia in Older People (EWGSOP) criteria; balance assessment on a force platform under four conditions: feet apart and eyes opened (FHEO), feet apart and eyes closed (FHEC), unstable surface, and single-leg stance; sEMG of lower-limb and trunk muscles, in addition to Timed Up and Go (TUG) and Brief Balance Evaluation Systems Test (Brief-BESTest). Results: The sarcopenia group showed greater postural instability, lower activation of the tibialis anterior and vastus medialis in specific tasks, greater activation of cervical/abdominal muscles during single-leg stance, and poorer TUG/Brief-BESTest performance.
[9] sEMG-based Sarcopenia risk classification using empirical mode decomposition and machine learning algorithms **ML/classification**	Kumar KS, Lee D, Jamsrandoj A, Soylu NN, Jung D, Kim J, Mun KR. (2024)	Objective: To classify sarcopenia risk from sEMG during functional activities. Methods: sEMG of rectus femoris (RF), biceps femoris (BF), tibialis anterior (TA), and gastrocnemius (GA) during normal walking, fast walking, standard squatting, and wide squatting; preprocessing, empirical mode decomposition (EMD), extraction of 36 features per signal and intrinsic mode function (IMF), minimum redundancy maximum relevance (mRMR) selection, and classification with K-nearest neighbors (KNN), Naive Bayes (NB), Random Forest, Extreme Gradient Boosting (XGB), and MLP using leave-one-subject-out cross-validation (LOSO-CV). Results: The best performance was obtained with MLP + EMD, with accuracy values of 0.88 (normal walking), 0.89 (fast walking), 0.81 (standard squatting), and 0.80 (wide squatting).
[31] Multi-channel EMG manifestations of upper-extremity muscle coordination imbalance among community-dwelling sarcopenic seniors. **Coordination/symmetry**	He H, Wu X, Li N, Jiang Y, He J, Jiang N. (2024)	Objective: To identify multichannel sEMG biomarkers that differentiate healthy older adults and older adults with sarcopenia during hand grip. Methods: sEMG from 6 forearm muscles during hand grip at 20% and 50% of maximal voluntary contraction (MVC); extraction of temporal and spectral features, normalization by MVC, and construction of the geometric index, Incenter-circumcenter distance of muscle coordination (ICDMC) to quantify multimuscular symmetry/coordination. Results: At 20% MVC, several ICDMC versions (based on root mean square (RMS), mean absolute value (MAV), slope sign changes (SSC), and waveform length (WL)) were significantly lower in sarcopenia, indicating more symmetric/consistent coordination patterns in the sarcopenia group.

**Table 2:** Comparison of studies, including the objective of the study, methods, and results (Part II).

Title and Analytical Label	Authors, Year	Objective, Methods, and Results
[32] Quadriceps muscle electromyography activity during physical activities and resistance exercise modes in younger and older adults **Activation**	Marshall RN, Morgan PT, Martinez-Valdes E, Breen L. (2020)	Objective: To compare relative quadriceps sEMG activity between younger and older adults during daily activities and three strength exercise modalities. Methods: Quadriceps sEMG recorded with textile shorts during walking, stair climbing, chair squats, elastic-band knee extension, and machine knee extension, normalized to the 1s peak MVC. Results: Older adults showed greater relative sEMG across all tasks; elastic-band extension produced activation similar to machine extension, and both clearly exceeded chair squat.
[33] Exploration of a machine learning approach for diagnosing sarcopenia among Chinese community-dwelling older adults using sEMG-based data **ML/diagnosis**	Li N, Ou J, He H, He J, Zhang L, Peng Z, Zhong J, Jiang N. (2024)	Objective: To develop a rapid sarcopenia screening/diagnostic method based on forearm sEMG during hand grip. Methods: Older adults classified according to the Asian Working Group for Sarcopenia 2019 (AWGS 2019) criteria, sEMG from 6 muscles during 20% and 50% MVC, extraction of 9 features (6 temporal and 3 time-frequency features based on CWT), normalization by MVC, and classification with a soft-voting ensemble of Support Vector Machine (SVM), RF, and Gradient Boosting Machine (GBM). Results: The Voting model achieved an accuracy of approximately 0.73, a sensitivity of approximately 0.79, and an area under the curve (AUC) of 0.78; WL and CWT_kurtosis were the most influential features according to Shapley Additive exPlanations (SHAP).
[34] Neural control properties of the external anal sphincter in young and elderly women **Neural control (MU)**	Dias N, Zhang C, Li X, Neshatian L, Orejuela FJ, Zhang Y. (2019)	Objective: To quantify the effect of age on neuromuscular control of the external anal sphincter in healthy women. Methods: Intrarectal high-density surface electromyography (HD-sEMG) with 64 channels during 6 maximal voluntary contractions of 10 s, k-means clustering convolution kernel compensation (KmCKC) decomposition, reconstruction of motor unit action potentials (MUAPs) by spike-triggered averaging (STA), and comparison between younger and older women. Results: Older women showed lower mean firing rate and greater MUAP amplitude, consistent with lower neural drive and compensation by larger motor units.
[28] Characteristics of the Electrophysiological Properties of Neuro-muscular Motor Units and Its Adaptive Strat-egy Response in Lower Extremity Muscles for Seniors with Pre-Sarcopenia: A Pre-liminary Study **Neural control (MU)**	Hu CH, Yang CC, Tu SJ, Huang IJ, Ganbat D, Guo LY. (2021)	Objective: To compare the electrophysiological properties of motor units in the vastus medialis among young adults, non-sarcopenic older adults, and older adults with pre-sarcopenia/risk. Methods: Multichannel decomposed electromyography (dEMG) during isometric knee extension at 50% MVC, Precision Decomposition III (PD III), and analysis of mean firing rate (MFR), recruitment threshold (RT), and the MFR-RT relationship. Results: No clear differences were found in the number of motor units or average MFR; however, the pre-sarcopenia group showed a higher firing rate per unit of force and a different MFR-RT slope, suggesting neuromuscular compensation.

**Table 3:** Comparison of studies, including the objective of the study, methods, and results (Part III).

Title and Analytical Label	Authors, Year	Objective, Methods, and Results
[35] Lower extremity muscle activation patterns in sarcopenic older adults during physical performance tests: implications for rehabilitation approaches **Activation + coordination**	Cai H, Zhang G, Wei L, Xu J, Yan F, Zhang M. (2025)	Objective: To compare lower-limb muscle activation patterns in older adults with sarcopenia and healthy controls during the Six-Minute Walk Test (6MWT) and the Five-Times Sit-to-Stand test (5STS). Methods: sEMG of 8 muscles, Vicon kinematics, force platforms, normalization to MVC, phase-specific analysis of the sEMG envelope, co-activation index, and between-trial variability. Results: The sarcopenia group walked more slowly and performed worse in 5STS, showing greater proximal and lower distal activation, greater antagonist co-activation, and task-specific changes in sEMG variability.
[36] Age-specific differences in the time-frequency representation of surface electromyographic data recorded during a sub-maximal cyclic back extension exercise: a promising biomarker to detect early signs of sarcopenia **Fatigue**	Habenicht R, Ebenbich-ler G, Bonato P, Koll-mitzer J, Ziegelbecker S, Unterlerchner L, Mair P, Kienbacher T. (2020)	Objective: To evaluate whether the time-frequency representation of lumbar sEMG during submaximal cyclic back extensions detects early changes associated with aging/sarcopenia. Methods: Healthy adults < 50 and >50 years performed lumbar extension MVC, an isometric contraction at 80% MVC, and 25 dynamic cycles at 50% MVC; bilateral sEMG at L1, L2, and L5; analysis using RMS and instantaneous median frequency (IMDF) with mixed models and test-retest reliability assessment. Results: The fatigue slope based on IMDF was clearly steeper in younger adults than in older adults, whereas RMS did not discriminate well by age.
[37] Effect of 6-Min Mastication Test on Masticatory Function in Young Ver-sus Older Adults: A Comparative SEMG Study **Activation + fatigue**	Chilwan U, Shameer S, Hanan A, Balasubrama-nium RK. (2025)	Objective: To compare masticatory function between young and older adults using a 6-min chewing test with sEMG. Methods: Bilateral sEMG recording of the masseter and submental region during continuous chewing of gum for 6 min; comparisons between groups and between minute 1 and minute 6. Results: Older adults showed poorer masseter performance in all sEMG variables and only a lower submental peak; within-group changes compatible with fatigue were observed, especially in the submental region.
[38] Age and sex specific effects in paravertebral surface electromyographic back extensor muscle fatigue in chronic low back pain **Fatigue**	Ebenbichler G, Habenicht R, Ziegelbecker S, Koll-mitzer J, Mair P, Kienbacher T. (2020)	Objective: To determine whether back extensor fatigue measured by sEMG during sustained isometric extension at 80% MVC shows age and sex-specific effects in chronic low back pain, and to assess its reliability. Methods: Bilateral sEMG at L1, L2, and L5 during a 30 s contraction, spectral analysis of median frequency (MF) with linear regression, together with lateral imbalance metrics and test-retest assessment. Results: The normalized MF fatigue slope was lower in older adults than in younger adults and showed sex effects, with very good relative reliability but high standard error of measurement (SEM) for some measures.

**Table 4:** Relationship between analysis type, group and article (A = Functional activation and motor control; B = Fatigue (temporal dynamics); C = ML: performance and biomarkers; D = Neural control and advanced coordination).

Analysis Label	Group	Reference
Activation	A	[32]
Activation + coordination	A	[35]
Posture + activation	A	[30]
Fatigue	B	[36,38]
Activation + fatigue	B	[37]
ML/biomarker	C	[8]
ML/classification	C	[9]
ML/diagnosis	C	[33]
Neural control (MU)	D	[28,34]
Coordination/symmetry	D	[31]

**Table 5:** Studied muscles using sEMG, study population, and database availability of Group A.

Article	Studied Muscles	Study Population	Database Availability
Sepúlveda-Loyola et al. (2025) [30]	Tibialis anterior (TA), gas-trocnemius (GA), vastus medialis (VM), gluteus medius (GM), erector spinae (ES), rectus abdominis (RA), external intercostal (INT), sternocleidomastoid (SCM), scalene (SC)	35 subjects with chronic obstructive pulmonary disease (COPD): 13 with sarcopenia and 22 without sarcopenia	No raw sEMG dataset is mentioned in a public repository, nor is it indicated that it can be requested from the authors
Marshall et al. (2020) [32]	Quadriceps of both legs, mainly vastus lateralis and vastus medialis	30 subjects: 15 young adults and 15 older adults	The article does not report a public repository nor indicate that raw sEMG data can be requested from the authors
Cai H et al. (2025) [35]	Gluteus maximus (GMax), gluteus medius (GMed), rectus femoris (RF), vastus medialis (VM), vastus lateralis (VL), biceps femoris (BF), gastrocnemius medialis (GA), tibialis anterior (TA)	316 older adults: 8 with sarcopenia and 8 healthy controls	The original data can be requested from the corresponding author

**Table 6:** Feature extraction methods, evaluation metrics, and results from the obtained signals of Group A.

Article	Evaluation Metrics	Feature Extraction Methods	Results
Sepúlveda-Loyola et al. (2025) [30]	sEMG expressed as RMS and relative normalized change %Δ*μ*VRMS Center of pressure dis-placement area (COParea), velocity in the anterior–posterior direction (Vel-AP), velocity in the medial–lateral direction (Vel-ML), amplitude in the anterior–posterior direction (Amp-AP), and amplitude in the medial-lateral direction (Amp-ML) Brief-BESTest, Timed Up and Go (TUG), hand grip strength (HGS), and 4 m gait speed (4MGS)	Trigno™ system (Delsys) Gain (G) = 1000 BP: 25–450 Hz fs = 2 kHz Normalization with respect to bipedal stance with eyes opened and expressed as %Δ*μ*VRMS Analysis under four balance conditions: feet apart and eyes opened (FHEO), feet apart and eyes closed (FHEC), unstable surface (US), and one-legged stance (OLS)	Sarcopenia in patients with COPD is associated with poorer postural control, reduced functional performance, and decreased muscle acti-vation in the lower limbs
Marshall et al. (2020) [32]	sEMG in *μ*V, relative sEMG (%) with respect to the maximum sEMG obtained during maximal voluntary contraction (MVC) MVC torque, knee extension torque relative to body mass (KET/BM), 12-repetition maximum (12-RM) with elastic band and machine, and rating of perceived exertion (RPE)	Signals recorded using textile-embedded sEMG shorts Pre-amplified signal BP: 40–300 Hz Signal rectified and averaged Non-overlapping windows of 100 ms Procedure described as similar to the root mean square (RMS) method Mean rectified amplitude obtained from the beginning to the end of each task Signal is also expressed as a percentage relative to the maximum sEMG obtained during a 1s peak period in MVC	The study concludes that Elastic Band Resistance Exercise Training (EB-RET) can induce muscle activation comparable to Machine Resistance Exercise Training (MN-RET), making it an accessible and pragmatic alternative for older adults These findings support the use of elastic band exercises as a potential strategy to counteract muscle loss associated with aging and sarcopenia
Cai H et al. (2025) [35]	Gait (6MWT): walking speed, non-dimensional walking speed, stride length, leg-normalized stride length, cadence, stride time, and stance phase percentage 5STS: total time, average repetition time, and duration of forward, upward, downward, and backward phases sEMG: muscle activation amplitudes, maximum EMG value, mean EMG value, co-activation index (CI) for RF/BF, VM/BF, VL/BF, and TA/GA pairs, and variability of activation (MeanSD)	Maximal voluntary contraction (MVC) used to normalize sEMG signals for each muscle BP: 20–450 Hz, full-wave rectification, and smoothing using a 6 Hz low-pass Butterworth filter Temporal interpolation to 101 points for gait and 5STS data Averaging of five gait cycles and three 5STS trials per participant Feature extraction: signal envelope, maximum and mean envelope values, co-activation indices, and variability across repetitions/cycles	The study concludes that sarcopenia involves not only muscle mass loss but also complex neuromuscular alterations, characterized by greater reliance on proximal muscles, increased antagonist co-activation, and task-specific control patterns

**Table 7:** EMG-derived features used as metrics of muscle activation or postural effects associated with sarcopenia.

EMG Feature	Sepúlveda- Loyola et al., 2025 [30]	Marshall et al., 2020 [32]	Cai et al., 2025 [35]	Interpretation Regarding Muscle Function/Sarcopenia
RMS (normalized %Δ*μ*VRMS)	✓	—	✓	Instantaneous/relative activation for postural tasks; lower distal RMS in sarcopenia → instability
MAV/mean rectified amplitude	—	✓	✓	Mean activation magnitude during tasks; higher % of max in older adults = greater relative effort
iEMG (integrated EMG/cumulative activity)	—	✓	—	Cumulative muscle work over task; indicates total neuromuscular demand
%MVC (EMG normalized to MVC)	—	✓	✓	Activation relative to individual capacity; sarcopenic subjects show higher %MVC for same tasks (reduced reserve)
Co-activation index (CI)	—	—	✓	Antagonist/agonist simultaneous activation; ↑ CI in sarcopenia → compensatory stabilization, reduced efficiency
Variability (MeanSD across cycles)	—	—	✓	Motor control consistency; ↑ variability (proximal) in gait for sarcopenia indicates altered control; ↓ variability in 5STS suggests rigid strategy

**Table 8:** Studied muscles using sEMG, study population, and database availability of Group B.

Article	Studied Muscles	Study Population	Database Availability
Habenicht R et al. (2020) [36]	Three lumbar levels: L1: iliocostalis lumborum L2: longissimus L5: multifidus	56 participants with a complete dataset free of artifacts	The authors indicate that anonymized data are available upon request
Chilwan U et al. (2025) [37]	Masseter muscle and submental muscle region, targeting the suprahyoid muscle group	50 participants: 25 healthy young adults (18–35 years) 25 healthy older adults (≥60 years)	The authors indicate that the datasets are available upon reasonable request to the corresponding author
Ebenbichler G et al. (2020) [38]	Lumbar paravertebral extensor muscles: L5: multifidus L2: longissimus L1: iliocostalis lumborum/ili-olumbalis gastrocnemius medialis (GA) tibialis anterior (TA)	117 young subjects and 112 older subjects, with an approximate distribution of 58 young women and 56 older women	The article does not explicitly report a public dataset nor a formal data request mechanism

**Table 9:** Feature extraction methods, evaluation metrics, and results from the obtained signals of Group B.

Article	Evaluation Metrics	Feature Extraction Methods	Results
Habenicht R et al. (2020) [36]	Lumbar extension MVC Hand grip strength Initial RMS-sEMG and temporal slope Initial IMDF-sEMG and temporal slope Fatigue slopes normalized to the intercept/onset Variables calculated separately for shortening (concentric) and lengthening (eccentric) phases Standard error of measurement (SEM) Coefficient of dependability (D) as a relative reliability measure (ICC-like) BORG fatigue rating at the end of the cyclic exercise Range of motion (ROM) and movement velocity	Active Trigno electrodes (Delsys) fs = 2000 Hz High-pass filter (HP): 20 Hz Low-pass filter (LP): 500 Hz Signals segmented according to movement phases using accelerometry Extraction of root mean square (RMS) and instantaneous median frequency (IMDF) using Cohen–Posch class time–frequency analysis Linear fitting to obtain temporal slopes of RMS-sEMG and IMDF-sEMG across 25 cycles Slopes normalized with respect to the initial value/intercept Averaging across the three lumbar levels	Maximal lumbar extension strength was comparable between younger and older adults RMS amplitude was considered a less sensitive variable for detecting early changes The temporal slope of IMDF-sEMG showed age-dependent differences The authors interpret these findings as early functional changes associated with aging and possible loss of fast motor units
Chilwan U et al. (2025) [37]	Mean average amplitude Peak amplitude MVC power Comparison between minute 1 and minute 6 of the 6-min mastication test (6MMT), and between young and older adults	Participants performed a 6MMT, chewing gum continuously for 6 min Synchronization with a metronome at 80 bpm Pre-chewing task of 40 s followed by 80 s rest before the main test Chattanooga VitalStim Plus 4-channel system Parameters automatically provided by the software	The authors interpret that aging mainly affects the masseter muscle The submental region primarily shows changes related to task-induced fatigue Sarcopenia, neural degeneration, and increased non-contractile tissue may explain the reduced performance in older adults
Ebenbichler G et al. (2020) [38]	Median frequency sEMG (MF-sEMG) onset MF-sEMG slope and MF-sEMG slope normalized to onset as indicators of neuromuscular fatigue Lumbar extension MVC Hand grip strength Balance: uncompensated imbalance and compensated imbalance Control: trunk position and position change during sustained contraction Questionnaires: Pain intensity, Roland Morris Disability Questionnaire (RMDQ), Pain Disability Index (PDI), and International Physical Activity Questionnaire (IPAQ)	Sustained isometric lumbar extension at 80% MVC for 30 s in a seated position with slight trunk flexion Trigno sensors (Delsys) fs = 2000 Hz BP: 20–450 Hz First 3 s discarded Fourier transform (FT) with Blackman window, using 500 ms windows with 50% overlap Median frequency (MF) calculation Linear regression applied to MF decay between 3 and 30 s Slope expressed in Hz/s and normalized to the initial value (%/s)	Lumbar extensor muscle fatigue measured by MF-sEMG was significantly lower in older participants The authors interpret this as a potential indicator of reduced glycolytic muscle activity and aging- and sarcopenia-related changes They conclude that MF-sEMG, even in the presence of chronic low back pain (cLBP), could be developed as an early biomarker of lumbar muscle function at risk of sarcopenia

**Table 10:** Comparison of EMG metrics used in Group B to assess fatigue and fatigability and their relationship with sarcopenia. Part I.

EMG Feature	Habenicht et al., 2020 [36]	Chilwan et al., 2025 [37]	Ebenbichler et al., 2020 [38]	Interpretation Regarding Sarcopenia
Root Mean Square (RMS)	Used to evaluate fatigability through amplitude changes during cyclic exercise. Limited sensitivity for detecting age-related differences.	Not evaluated.	Used to assess fatigability; however, RMS showed limited discriminative ability between younger and older adults.	Reflects motor unit recruitment and activation level. Reduced responsiveness may indicate diminished neuromuscular reserve associated with sarcopenia.
Mean Average Amplitude	Not evaluated.	Used to assess both fatigue and fatigability through changes during the 6-Minute Mastication Test.	Not evaluated.	Lower amplitude suggests reduced muscle activation capacity and impaired neuromuscular function, consistent with sarcopenic muscle deterioration.
Peak Amplitude	Not evaluated.	Used as a marker of fatigue, comparing younger and older adults.	Not evaluated.	Reduced peak activation reflects diminished ability to generate maximal muscle activation, a characteristic of age-related muscle decline.
EMG Power	Not evaluated.	Used to assess both fatigue and fatigability.	Not evaluated.	Represents cumulative electrical activity; lower values indicate reduced muscular work capacity associated with impaired muscle quality.
Maximum Voluntary Contraction (%MVC)	Used only to normalize exercise intensity (50% MVC); not analyzed as a fatigue metric.	Used as a functional indicator of muscle performance.	Used only for normalization of exercise intensity.	Declining MVC reflects reduced force-generating capacity, one of the functional hallmarks of sarcopenia.
Median Frequency (MF/IMDF)	Used as an indicator of fatigue through spectral shifts during sustained contractions.	Not evaluated.	Decreasing median frequency reflects reduced muscle fiber conduction velocity during fatigue.	
Median Frequency Slope (MF slope/IMDF slope)	Primary biomarker of fatigability.	Not evaluated.	Primary outcome for assessing fatigability.	A less negative slope in older adults suggests reduced recruitment of fast-twitch (Type II) fibers and lower conduction velocity, physiological features commonly associated with sarcopenia.

**Table 11:** Comparison of EMG metrics used in Group B to assess fatigue and fatigability and their relationship with sarcopenia. Part II.

EMG Feature	Habenicht et al., 2020 [36]	Chilwan et al., 2025 [37]	Ebenbichler et al., 2020 [38]	Interpretation Regarding Sarcopenia
MF/IMDF Onset	Baseline reference only.	Not evaluated.	Baseline reference only.	Used to normalize fatigue-related changes rather than representing fatigue itself.
Muscle Imbalance Indices (Compensated and Uncompensated)	Used to evaluate bilateral activation symmetry rather than fatigue.	Not evaluated.	Used similarly to characterize muscle activation asymmetry.	May reflect compensatory motor strategies but are not direct biomarkers of sarcopenia.

**Table 12:** Studied muscles using sEMG, study population, and database availability of Group C.

Article	Studied Muscles	Study Population	Database Availability
Song K et al. (2024) [8]	Rectus femoris (RF)	98 healthy participants aged 20 to 60 years, including 48 men (49%) and 50 women (51%)	The article does not explicitly re-port a public dataset nor a formal data request procedure
Kumar KS et al. (2024) [9]	Rectus femoris (RF) Biceps femoris (BF) Tibialis anterior (TA) Gastrocnemius (GA)	93 adults aged 20 to 59 years: 49 in the control group 44 in the at-risk sarcopenia group	The article does not explicitly report a public repository nor a formal data request mechanism
Li N et al. (2024) [33]	Brachioradialis (BRA) Flexor carpi radialis (FCR) Flexor digitorum superficialis (FDS) Flexor carpi ulnaris (FCU) Extensor carpi ulnaris (ECU) Extensor digitorum (ED)	The final model was trained on older adults (≥60 years): 45 healthy and 48 with sarcopenia	The authors indicate that anonymized data are available upon request

**Table 13:** Feature extraction methods, evaluation metrics, and results from the obtained signals of Group C (Part I).

Article	Evaluation Metrics	Feature Extraction Methods	Results
Song K et al. (2024) [8]	Digital biomarker defined as muscle strength/muscle mass Appendicular skeletal muscle mass index (AS-MI) Right leg muscle mass Knee extension and flexion force Model performance metrics: Pearson correlation coefficient (r), mean error (ME), standard deviation of the error (STDE), and Bland–Altman analysis Muscle mass measured using bioelectrical impedance analysis (BIA) Reference label combining muscle strength measured with a leg muscle dynamometer	Non-invasive wearable system: electrical stimulation at 5, 10, 15, 20, 25, and 30 Hz fs = 1000 Hz, 8-bit ADC Offset removal SMCS segmentation Spectrogram calculation Extraction of muscle contraction pattern (MCP) features: envelope, peak detection, autocorrelation, and auto-correlation slope Transformation into images using continuous wavelet transform (CWT) with a third-order Gaussian derivative wavelet Deep feature extraction using a convolutional neural network (CNN) Concatenation of MCP features + CNN/CWT features + body information Final training using a multilayer perceptron (MLP) regression model	The study showed that the proposed digital biomarker can be robustly estimated The best model, integrating body information, MCP features, and CWT/CNN features, achieved a Pearson correlation of 0.89 between the reference label and model estimation Mean error was −0.06 and STDE was 0.68
Kumar KS et al. (2024) [9]	Reference variables: Age, Weight, Height, BMI, ASMI, knee extension and flexion force Classifier performance metrics: Accuracy, Precision, Recall, F1-score, Confusion matrix 36 EMG features per signal, including time, frequency, and time–frequency domain variables: MAV, RMS, VAR, STD_DEV, KURT, SKEW, WAMP, SSC, WL, SSI, AAC, DASDV, MYOP, MFL, LD, IEMG, IQR, CARD, H, SE, SD1, SD2, ZC, MNF, MDF, TTP, MNP, PKF, and MAXP	DELSYS Trigno Wireless system fs = 1259 Hz, IMU at 148 Hz Four physical activities: normal walking, fast walking, standard squat, and wide squat BP: 20–400 Hz sEMG/IMU synchronization Event-based segmentation using IMU angular signal Normalization with respect to MVC during wide squat Full-wave rectification Signal decomposition using empirical mode decomposition (EMD) into intrinsic mode functions (IMFs); first five IMFs used Evaluation of normalized sEMG and sEMG + IMFs Feature selection using minimum redundancy maximum relevance (mRMR) Classifiers with leave-one-subject-out cross-validation (LO-SO-CV): KNN, Naive Bayes, Random Forest, XGBoost, and MLP	The study showed that combining sEMG + EMD + machine learning ena-bles reliable classification of sarcopenia risk during daily activities Best performance obtained with MLP using EMD-based features Reported accuracies: 0.88 (normal walking), 0.89 (fast walking), 0.81 (standard squat), and 0.80 (wide squat) IMF-derived features improved performance by approximately 4–5% compared to non-EMD features MLP and XGBoost were consistently the best-performing classifiers

**Table 14:** Feature extraction methods, evaluation metrics, and results from the obtained signals of Group C (Part II).

Article	Evaluation Metrics	Feature Extraction Methods	Results
Li N et al. (2024) [33]	Clinical reference metrics for sarcopenia screening: skeletal mus-cle index (SMI) via BIA, grip strength (GS), and 5-times chair sit-to-stand test (5TCST) Nine representative sEMG features Time domain (6): RMS, MAV, iEMG, WL, ZC, SSC Time–frequency domain (3): CWT_power, CWT_kurtosis, WE Classification evaluation: Accuracy, Sensitivity, Specificity, F1-score, and ROC-AUC	Wireless Ultium sEMG system (Noraxon) fs = 2000 Hz G = 1000 Average of three MVC trials Tasks at 20% and 50% MVC Notch filters at multiples of 50 Hz BP filter: 10–500 Hz Visual selection of a 3-s stationary segment per contraction 200 ms windows with 50 ms overlap MVC normalization per channel using session 1 MVC trials CWT coefficient calculation using Morlet wavelet Voting classifier (soft voting ensemble): SVM, Random Forest, and Gradient Boosting Machine Evaluation using five-fold cross-validation Model interpretability using SHAP	The study showed that forearm sEMG features during hand grip can moderately distinguish between healthy older adults and those with sarcopenia Voting classifier achieved the best performance: Accuracy = 0.73 ± 0.07, Sensitivity = 0.79 ± 0.07, Specificity = 0.67 ± 0.07, F1-score = 0.73 ± 0.06, AUC = 0.78 SHAP analysis identified waveform length (WL) and CWT_kurtosis as the most influential features These results are interpreted as reflecting changes in motor unit activation

**Table 15:** EMG-derived features used as machine learning inputs for Group C. Part I.

EMG Feature	Kumar et al., 2024 [9]	Li et al., 2024 [33]	Song et al., 2024 [8]	Interpretation Regarding Muscle Function/Sarcopenia
RMS	✓	✓	—	Overall muscle activation
MAV	✓	✓	—	Mean activation level
iEMG	✓	✓	—	Cumulative muscle activity
Waveform Length	✓	✓	—	Neuromuscular complexity
Zero Crossing	✓	✓	—	Motor unit firing characteristics
Slope Sign Changes	✓	✓	—	Signal complexity
Willison Amplitude	✓	—	—	Motor unit recruitment
Median Frequency	✓	—	—	Muscle fiber conduction velocity
Mean Frequency	✓	—	—	Spectral fatigue descriptor
Sample Entropy	✓	—	—	Signal complexity
Wavelet Features	✓	✓	✓ (CWT images)	Time-frequency representation
CWT Kurtosis	—	✓	—	High predictive value
Muscle Contraction Pattern (MCP) features	—	—	✓	Muscle response pattern elicited by electrical stimulation

**Table 16:** EMG-derived features used as Machine Learning inputs for Group C. Part II.

EMG Feature	Kumar et al., 2024 [9]	Li et al., 2024 [33]	Song et al., 2024 [8]	Interpretation Regarding Muscle Function/Sarcopenia
Original Envelope (OE)	—	—	✓	Envelope morphology
Normalized Envelope (NE)	—	—	✓	Standardized muscle response
Body Information (Age, Sex, Height, Weight)	✓	✓	✓	Improves prediction performance

**Table 17:** Machine learning techniques used for sensorimotor performance evaluated with EMG.

ML Technique	Kumar et al., 2024 [9]	Li et al., 2024 [33]	Song et al., 2024 [8]	Purpose
Random Forest	✓	✓	—	Classification
XGBoost	✓	—	—	Classification
KNN	✓	—	—	Baseline classifier
Naïve Bayes	✓	—	—	Baseline classifier
Multilayer Perceptron	✓	—	✓	Classification/Regression
CNN	—	—	✓	Automatic feature extraction from CWT images
SVM	—	✓	—	Ensemble classifier
Gradient	—	✓	—	Classification
Voting Ensemble	—	✓	—	Ensemble classification

**Table 18:** Complementary preprocessing methods for the machine learning techniques used for sensorimotor performance evaluated with EMG.

ML Technique	Kumar et al., 2024 [9]	Li et al., 2024 [33]	Song et al., 2024 [8]	Purpose
mRMR	✓	—	—	Feature selection
SHAP	—	✓	—	Explainability
Pearson Correlation	—	—	✓	Feature relevance analysis
Three-fold Cross Validation	—	—	✓	Model validation
Five-fold Cross Validation	—	✓	—	Model validation
LOSO Cross Validation	✓	—	—	Subject-independent validation

**Table 19:** Performance metrics of the machine learning models.

Performance Metric	Kumar et al., 2024 [9]	Li et al., 2024 [33]	Song et al., 2024 [8]	Purpose
Accuracy	✓	✓	—	Classification performance
Precision	✓	✓	—	Positive prediction reliability
Recall	✓	✓	—	Sensitivity
Specificity	—	✓	—	Healthy subject identification
F1-score	✓	✓	—	Precision–recall balance
ROC-AUC	—	✓	—	Class discrimination
Confusion Matrix	✓	✓	—	Classification errors
Mean Error (ME)	—	—	✓	Regression accuracy
Standard Deviation of Error (STDE)	—	—	✓	Regression variability
Pearson Correlation (r)	—	—	✓	Agreement between predicted and reference biomarker
Bland–Altman Analysis	—	—	✓	Agreement analysis
*p*-value for correlation	—	—	✓	Statistical significance

**Table 20:** Summary of the articles considered in Group C dedicated to machine learning (ML) applications using sEMG. Part I.

Field	Song et al., 2024 [8]	Kumar et al., 2024 [9]	Li et al., 2024 [33]
Article identifier	“Digital Biomarker for Muscle Function Assessment Using Surface Electromyography With Electrical Stimulation and a Non-Invasive Wearable Device”	“sEMG-based Sarcopenia risk classification using empirical mode decomposition and machine learning algorithms”	“Exploration of a machine learning approach for diagnosing sarcopenia among Chinese community-dwelling older adults using sEMG-based data”
Class sizes and imbalance	98 Participants total; 48 men, 50 women; No other class labels; Sex nearly balanced.	Total n = 93; Control group = 49, Sarcopenia-risk n = 44. Sex distribution (47.3%/52.7%) approx.: 44 men/49 women.	After QC, Total n = 93 were analyzed: Healthy n = 45, Sarcopenic n = 48. Demographics provided in Tables.
Reference standards	(1) Muscle strength: leg muscle dynamometer (BS-LS, InBody); (2) Muscle mass: BIA (InBody770).	Grouping derived from BMI and self-reported physical activity questionnaire; ASMI reported but no gold-standard imaging (DXA) used for labelling; Authors note this limitation.	AWGS 2019 diagnostic criteria. Muscle mass by BIA (InBody770), Grip strength by CAMRY dynamometer, physical performance via 5× chair stand. Sarcopenia defined per AWGS: low muscle mass plus low strength and/or low performance.
Subject-level vs. Window-level splitting	Subject-level splitting: Three-fold CV assigned by participant number.	Subject-level splitting: Leave-One-Subject-Out Cross Validation (LOSO-CV).	Subject-level. A 5-fold Cross Validation with Shuffle Split to balance folds; Leave-one-out CV inside each fold for evaluation across participants. Overall evaluation reported across 93 subjects.
Timing of preprocessing	Preprocessing: Offset removal, segmentation by stimulation frequency, envelope/spectrogram/CWT extraction; artifact removal not performed.	Not reported, but preprocessing steps described (bandpass Butterworth 20–400 Hz, segmentation using IMU angular velocity, synchronization with IMU, rectification, normalization to MVC).	Preprocessing: channel inspection (expert), notch filters (multiples of 50 Hz), bandpass 10–500 Hz Butterworth, selection of 3-s stationary segment, extraction of 200-ms windows with 50 ms increment, MVC-based normalization (channel-specific).
Feature selection	Features selected if absolute correlation with digital biomarker > 0.35; selection determined by voting across folds (three-fold CV). It is stated that feature selection was performed and selected features were determined by voting across all folds (implies selection within cross-validation, but the description is ambiguous about whether selection was nested).	Feature extraction was performed after EMD decomposition (first five IMFs) and raw normalized EMG; then mRMR feature selection was applied to derive optimal subsets. mRMR was applied to obtain the best subset used for training; it does not explicitly state whether feature selection was performed inside each LOSO training fold or on the whole dataset.	Feature extraction: Nine representative features (six Hudgins time-domain + three CWT time-frequency features). Channel selection and feature selection were applied (removing the three lowest-SHAP features and selecting channel combinations 2 and 3); however, it is not explicitly stated whether feature selection (channel/feature removal) was performed strictly within training folds or on the whole dataset prior to CV.

**Table 21:** Summary of the articles considered in Group C dedicated to machine learning (ML) applications using sEMG. Part II.

Field	Song et al., 2024 [8]	Kumar et al., 2024 [9]	Li et al., 2024 [33]
Hyper-parameter tuning approach	Training details include model architecture choices (MLP with two hidden layers of 64 units, dropout 0.5), number of epochs (50), activation (ELU), and loss (MSE). However, an explicit hyperparameter-tuning procedure (grid search, validation-based tuning, or which parameters were tuned) is not specified.	Hyperparameters optimized using GridSearchCV; parameter grids not reported, and nesting relative to LOSO not specified.	Grid-Search used for classifier hyperparameters and voting weights (weights optimized to maximize sensitivity). Hyperparameter grids and nesting are not detailed.
Whether nested CV was used	Not specified/not used. Only three-fold CV described; no nested CV reported.	Not specified. GridSearchCV used, but nested-CV relative to LOSO not described.	Not specified. Nested CV not explicitly described, although they used inner leave-one-out within folds and Grid-Search.
Presence-/absence of external validation	Absent. No external dataset reported.	Absent. Only internal dataset used.	Absent. No external validation cohort.
Confidence intervals for reported metrics	Reported metrics: Pearson correlation coefficient (r), mean errors (ME) and standard deviation (SD) of error, Bland–Altman plots, and *p*-values for correlations. No confidence intervals are reported.	Not reported. Metrics presented as point estimates (accuracy, precision, recall, F1) and confusion matrices; no CIs.	Not reported. Metrics shown as mean ± SD across CV folds; AUC reported as point estimate (0.78); no 95% CIs.
Calibration measures Thresholds	Not reported. Bland–Altman used for agreement; no formal calibration statistics. Not applicable (regression task).	Not reported calibration metrics. Not applicable This is a binary classifier with class labels; authors report accuracy/precision/recall. Probability threshold choice not stated.	An “Optimal Operating Point (OOP)” on ROC chosen to prioritize sensitivity with specificity > 0.65; numerical probability threshold not reported.
Code/data availability	Not specified. No data/code repository link provided.	Not specified; no link or repository for code/data.	Data availability: anonymized data available upon request. Code: not specified; software packages listed (Python version 3.11.3, Scikit-learn version 1.3.0, PyWavelets version 1.4.1, and Shap version 0.42.1).
Commentary on possible leakage risks	No explicit dedicated discussion of leakage risks. Potential leakage points identified: Feature selection voting across folds is ambiguous and may have used test data; Unclear whether preprocessing/selection occurred inside folds. Model selection criteria not fully detailed.	No explicit discussion of data leakage risks. Potential leakage: Unclear whether mRMR feature selection. GridSearchCV was performed inside LOSO training folds; if applied to full data, test-subject info may leak; Nesting of hyperparameter tuning relative to LOSO not described.	No explicit discussion of leakage Concrete potential leakage: feature removal based on SHAP computed using all 93 participants suggests selection may have been informed by test data. Unclear whether Grid-Search was nested within CV; Paper lacks explicit statements ensuring selection and tuning were performed solely within training folds.

**Table 22:** Studied muscles using sEMG, study population, and database availability of Group D.

Article	Studied Muscles	Study Population	Database Availability
He H et al. (2024) [31]	Six forearm muscles: Brachioradialis (BRA) Flexor carpi ulnaris (FCU) Flexor carpi radialis (FCR) Extensor carpi ulnaris (ECU) Flexor digitorum superficialis (FDS) Extensor digitorum (ED)	153 older adults aged 60 to 91 years: 55 in the sarcopenia group 98 in the control group	No datasets were reported
Dias N et al. (2019) [34]	External anal sphincter (EAS)	26 healthy women: 13 young (31.0 ± 3.6 years) 13 older adults (64.3 ± 6.2 years)	Not reported
Li N et al. (2024) [33]	Brachioradialis (BRA) Flexor carpi radialis (FCR) Flexor digitorum superficialis (FDS) Flexor carpi ulnaris (FCU) Extensor carpi ulnaris (ECU) Extensor digitorum (ED)	The final model was trained on older adults (≥60 years): 45 healthy and 48 with sarcopenia	Not reported
Hu C et al. (2021) [28]	Vastus medialis (VM)	22 participants: 5 in the at-risk sarcopenia group (RS) 5 in the non-sarcopenia group (NS) 12 in the young group (YG)	Not reported

**Table 23:** Feature extraction methods, evaluation metrics, and results from the obtained signals of Group D (Part I).

Article	Evaluation Metrics	Feature Extraction Methods	Results
He et al. (2024) [31]	Clinical and functional variables: Age, BMI, calf circumference, height, weight, hand grip strength, 5-time sit-to-stand test (5-STS), and skeletal muscle mass index (SMI) Time-domain sEMG features: RMS, MAV, ZC, IEMG, SSC, WL Frequency-domain sEMG features: MPF, MDF Main proposed metric: incenter–circumcenter distance of muscle coordination (ICDMC) as a multi-muscle asymmetry/coordination index A lower ICDMC indicates a more symmetric muscle coordination pattern	Ultium sEMG system (Noraxon) Hand grip task: 3 MVC trials and 4 trials at 20% and 50% MVC in random order Notch filter at 50 Hz and BP filter: 20–500 Hz Visual inspection by an expert Extraction of 1s stable segment for MVC and 3 s stable segments for 20% and 50% MVC Sub-segmentation into 200 ms windows with 50 ms step Feature computation and averaging per segment Normalization using mean MVC values Spider plots/hexagons per condition Geometric ratios derived from hexagon symmetry ICDMC computed using perimeter, area, inradius, and circumradius	Older adults with sarcopenia showed more symmetric and consistent multichannel patterns than healthy controls, reflected by lower ICDMC values, especially at low intensity (20% MVC) The study concludes that sarcopenia alters upper limb muscle coordination and that ICDMC can capture these differences, with better discriminative capacity at low submaximal contractions
Dias N et al. (2019) [34]	Neuromuscular metrics: mean motor unit firing rate (pulses per second, PPS), motor unit action potential amplitude (MUAP amplitude) Average number of decomposed motor units (MUs) per subject Relationships between age–firing rate and age–MUAP amplitude via linear regression	64-channel intra-rectal probe fs = 5000 Hz Amplification using BrainVision amplifier Participants performed 6 repetitions of MVC of the EAS for 10 s, with sufficient rest to avoid fatigue BP: 10–400 Hz Notch filter at 50 Hz Segmentation of con-traction periods Signal decomposition using k-means clustering convolution kernel compensation (KmCKC) to obtain MUAP spike trains MUAP waveform reconstruction using spike-triggered averaging (STA) Firing rate computed as mean discharges per second Visual identification of innervation zone channel from reconstructed bipolar map MUAP size estimated using the largest negative peak above the innervation zone	HD-sEMG decomposition was successful in all analyzed participants Young women showed significantly higher mean MU firing rates than older women MUAP amplitude was significantly higher in older adults Both variables differed significantly between groups (*p* < 0.05) Linear regression showed that age was negatively associated with firing rate and positively with MUAP amplitude Findings suggest age-related impairment in neural control of the EAS, with reduced descending excitation and compensatory increases at the motor unit level

**Table 24:** Feature extraction methods, evaluation metrics, and results from the obtained signals of Group D (Part II).

Article	Evaluation Metrics	Feature Extraction Methods	Results
Hu C. et al. (2021) [28]	Clinical and functional variables: ASM, gait speed, grip strength, MVC, PASE, IPAQ Motor unit number Recruitment threshold (RT) Slope of mean firing rate (MFR) vs. RT Y-intercept of MFR vs. RT relationship Mean firing rate (MFR) Firing rate per unit force Muscle fiber discrimination (MFD) Force variance as an index of stability/performance	Bagnoli 16-channel sEMG system (Delsys) using a single four-channel sensor placed over the vastus medialis (VM) Precision Decomposition III (PD III) algorithm used to extract motor unit spike trains Only motor units with >90% decomposition accuracy included MVC and 50% MVC knee extension tasks: 5 s rest, 4 s ramp-up, 10 s steady contraction, 4 s ramp-down, 5 s rest From decomposed signals: MFR computed during the 10 s steady phase RT determined from first discharge Linear regression MFR vs. RT (slope and intercept) MFD: temporal integral of firing rate to infer type I/type II functional classification Firing rate per unit force = mean firing rate/50% MVC force Force variance during steady contraction	Differences were found in variables reflecting neuromuscular adaptation The at-risk sarcopenia group showed a significantly steeper MFR vs. RT slope compared to young subjects Firing rate per unit force was significantly higher in the at-risk group than in young individuals Results suggest a compensatory neuromuscular strategy in aging and pre-sarcopenia, with relative preservation of type I fibers and increased neural drive required to maintain force output

**Table 25:** EMG features used in muscle activation and co-coordination related to sarcopenia. Part I.

EMG Feature	He, H., 2024 [31]	Dias, N., 2019 [34]	Hu, C., 2021 [28]	Interpretation Regarding Muscle Control/Sarcopenia
RMS	✓	—	✓	Signal energy/amplitude — reflects overall activation level; changes indicate altered force generation or fiber composition
MAV/IEMG	✓	—	✓ (IEMG listed)	Mean amplitude—similar to RMS; sensitive to subcutaneous tissue but useful to detect loss of mass/activation
WL (wavelength)	✓	—	✓	Time–frequency feature reflecting spectral content and changes in conduction/fast fiber contribution
SSC (slope sign changes)	✓	—	—	Index of high-frequency transients; alterations indicate modifications in recruitment or coordination
ZC (zero crossings)	✓	—	—	Frequency-related indicator; may reflect recruitment changes or fatigue
MDF/MPF	—	—	—	Frequency metrics related to conduction
(median/mean frequency)	(extracted but not discriminative)	—	—	velocity/type II fiber proportion; may decrease with loss of fast fibers
ICDMC (incenter–circumcenter distance) — novel index	✓	—	—	Multichannel symmetry/variability index: smaller in sarcopenic subjects → more uniform patterns (possible type I predominance/altered recruitment)
Mean firing rate (MFR/PPS)	—	✓	✓	Motor unit discharge rate; decreases with age, indicating reduced central drive; changes reflect compensation or neural deterioration
Firing rate per unit force (pps/N)	—	—	✓	Increased value indicates higher discharge required per force unit → compensatory response to atrophy; potential early sarcopenia marker
Slope and y-intercept (MFR vs. RT)	—	—	✓	Describes MFR–recruitment relationship: changes associated with fatigue, MU remodeling, and fiber composition
Recruitment threshold (RT)	—	— (MUAP amplitude/size reported)	✓ (MU counts, etc.)	Loss of MUs and increase in MU size indicate denervation and reinnervation (sarcopenic remodeling)

**Table 26:** EMG features used in muscle activation and co-coordination related to sarcopenia. Part II.

EMG Feature	He, H., 2024 [31]	Dias, N., 2019 [34]	Hu, C., 2021 [28]	Interpretation Regarding Muscle Control/Sarcopenia
MUAP amplitude/MU size	—	✓ (MUAP amplitude ↑ with age)	—	Increased amplitude suggests reinnervation/higher innervation ratio; marker of compensatory remodeling
Muscle fiber discrimination (MFD)/type I vs. II classification	—	—	✓	Indirect inference of type I vs. II predominance; useful to detect shifts in fiber composition (loss of type II)
Force variance/CV (stability)	—	—	✓	Measures force stability/precision; changes indicate impaired motor control
Time-domain multichannel symmetry (hexagon → combined features)	✓ (basis for ICDMC)	—	—	Assesses multi-muscle coordination; reveals more consistent patterns in sarcopenic subjects at low efforts

**Table 27:** Meta-analysis metrics for hand grip strength statistics for sarcopenic and control groups across the evaluated articles.

Study	Sub Group	N Control	N Case	MD	SE	Var
He (2024)_F [31]	Elderly Female	68	36	5.6	0.615	0.378
Li (2024)_F [33]	Elderly Female	29	31	8	0.750	0.56
He (2024)_M [31]	Elderly Male	30	19	8	1.174	1.37
Li (2024)_M [33]	Elderly Male	16	17	10.3	1.451	2.10
Sepulveda (2025) [30]	Elderly	22	13	4.7	2.273	5.16
He (2024) [31]	Elderly	98	55	5.9	0.995	0.99
Li (2024) [33]	Elderly	45	48	8.9	1.273	1.62
Hu (2021) [28]	Elderly	5	5	9.6	4.309	18.56

## Data Availability

The source code and Supplementary Material are available in the GitHub repository: https://github.com/c4macho/LIPS_ARTICULOS (accessed on 5 August 2026).

## Supplementary Materials

The following supporting information can be downloaded at: https://www.mdpi.com/article/10.3390/s26165121/s1.

## Abbreviations

The following abbreviations are used in this manuscript:

6MWT	Six-Meter Walk Test
A/D	Analog/Digital
ACCI	Age-adjusted Charlson Comorbidity Index
ADC	Analog-to-Digital Converter
ADL	Activities of Daily Living
AI	Artificial Intelligence
ALS	Amyotrophic Lateral Sclerosis
Amp-AP	Amplitude of the movement of center of pressure in anterior-posterior
	direction
Amp-ML	Amplitude of the movement of center of pressure in medial-lateral direction
ANOVA	Analysis of Variance
ASM	Appendicular Skeletal Muscle
ASMI	Appendicular Skeletal Muscle Mass Index
AUC	Area Under the Curve
AWGS	Asian Working Group for Sarcopenia
AWGS 2019	Asian Working Group for Sarcopenia 2019
BF	Biceps Femoris
BF%	Body Fat Percentage
BI	Body Information
BIA	Bioelectrical Impedance Analysis
BIVA	Bioelectrical Impedance Vector Analysis
BMI	Body Mass Index
BP	Band Pass filter
bpm	Beats Per Minute
BRA	Brachioradialis
Brief-BESTest	Brief Balance Evaluation Systems Test
BW-RET	Body-Weight Resistance Exercise Training
cLBP	Chronic Low Back Pain
CARD	Cardinality
CI	Co-Activation Index
CNN	Convolutional Neural Network
CNS	Central Nervous System
COP-area/COP-a	Centre of Pressure Displacement Area
CT	Computed Tomography
CWT	Continuous Wavelet Transform
CWT_kurtosis	Kurtosis of Continuous Wavelet Transform Coefficients
CWT_power	Absolute Power of Continuous Wavelet Transform
D	Coefficient of Dependability
DAMV	Difference Absolute Mean Value
DASDV	Difference Absolute Standard Deviation Value
dEMG	Decomposed Electromyography
DC/TMD	Diagnostic Criteria for Temporomandibular Disorders
DEXA	Dual-Energy X-ray Absorptiometry
DVARV	Difference Variance Value
DXA	Dual-Energy X-ray Absorptiometry
EAS	External Anal Sphincter
EB	Elastic Band
EB-RET	Elastic Band Resistance Exercise Training
ECU	Extensor Carpi Ulnaris
ED	Extensor Digitorum
EIM	Electrical Impedance Myography
EMD	Empirical Mode Decomposition
EMG	Electromyography
ES	Erector Spinae
EWGSOP	European Working Group on Sarcopenia in Older People
F1-score	F1 Score
FCU	Flexor Carpi Ulnaris
FCR	Flexor Carpi Radialis
FDS	Flexor Digitorum Superficialis
FFM	Fat-Free Mass
FFMI	Fat-Free Mass Index
FI	Fecal Incontinence
FHEC	Feet Apart and Eyes Closed
FHEO	Feet Apart and Eyes Opened
FMI	Fat Mass Index
FM	Fat Mass
fs	Sampling frequency
FT	Fourier Transform
G-Theory	Generalizability Theory
GA	Gastrocnemius/Gastrocnemius Medialis
GBM	Gradient Boosting Machine
GM	Gluteus Medius
GMax	Gluteus Maximus
GMed	Gluteus Medius
GS	Grip Strength
H	Hurst Exponent
HD-sEMG	High-Density Surface Electromyography
HG	Healthy Group
HGS	Hand Grip Strength
HP	High Pass filter
HS	Heel Strike
$Ht^{2}$	Squared Height
ICDMC	Incenter–Circumcenter Distance of Muscle Coordination
ICC	Intraclass Correlation Coefficient
ICW	Intracellular Water
iEMG	Intramuscular Electromyography
IEMG	Integrated Electromyography
IMDF	Instantaneous Median Frequency
IMF	Intrinsic Mode Function
IMU	Inertial Measurement Unit
INT	External Intercostal
IPAQ	International Physical Activity Questionnaire
IQR	Interquartile Range
IRB	Institutional Review Board
KET/BM	Knee Extension Torque Relative to Body Mass
KmCKC	k-means Clustering Convolution Kernel Compensation
KNN	K-Nearest Neighbors
KURT	Kurtosis
LD	Log Detector
LED	Light-Emitting Diode
LOSO-CV	Leave-One-Subject-Out Cross-Validation
LP	Low Pass filter
MAV	Mean Absolute Value
MAXP	Maximum EMG Power
MCP	Muscle Contraction Pattern
MDF	Median Frequency
ME	Mean Error/Average Energy
MEAN_AD	Mean Absolute Deviation
MED_AD	Median Absolute Deviation
MET	Metabolic Equivalent of Task
MF	Median Frequency
MF-SEMG	Median Frequency Surface Electromyography
MFD	Muscle Fiber Discrimination
MFL	Max Fractal Length
ML	Machine Learning
MLP	Multilayer Perceptron
MMAV	Modified Mean Absolute Value
MMAV2	Modified Mean Absolute Value 2
MN	Machine
MNF	Mean Frequency
MNP	Mean Power
MN-RET	Machine Resistance Exercise Training
MPF	Mean Power Frequency
MRI	Magnetic Resonance Imaging
MU	Motor Unit
MUAP/MUAPs	Motor Unit Action Potential/Motor Unit Action Potentials
MVC	Maximal Voluntary Contraction
MYOP	Myopulse Percentage Rate
NB	Naive Bayes
NMJ	Neuromuscular Junction
NRMSE	Normalized Root Mean Square Error
NS	Non-Sarcopenia Group
OLS	One-Legged Stance
PASE	Physical Activity Scale for the Elderly
PDI	Pain Disability Index
PD III	Precision Decomposition III
PDF	Probability Density Function
PKF	Peak Frequency
PPS	Pulses Per Second
QFM-group	Quadriceps Femoris Muscle Group
RA	Rectus Abdominis
RET	Resistance Exercise Training
RF	Rectus Femoris/Random Forest
RMDQ	Roland Morris Disability Questionnaire
RMSE	Root Mean Square Error
RMS	Root Mean Square
ROC	Receiver Operating Characteristic
ROM	Range of Motion
RPE	Risk-Sarcopenia Group
RT	Recruitment Threshold
SARC-F	Strength, Assistance with Walking, Rising from a Chair, Climbing Stairs,
	and Falls
SC	Scalene
SCM	Sternocleidomastoid
SD	Standard Deviation
SD1	Poincaré Plot Index 1
SD2	Poincaré Plot Index 2
SE	Sample Entropy
SEM	Standard Error of Measurement
SENIAM	Surface Electromyography for the Non-Invasive Assessment of Muscles
sEMG/SEMG	Surface Electromyography
SG	Sarcopenic Group
SHAP	SHapley Additive exPlanations
SIAS	Spina Iliaca Anterior Superior
SKEW	Skewness
SMI	Skeletal Muscle Index/Skeletal Muscle Mass Index
SMM	Skeletal Muscle Mass
SMMI	Skeletal Muscle Mass Index
SMCSs	Stimulated Muscle Contraction Signals
SPP	Superior Part of the Patella
SPPB	Short Physical Performance Battery
SSI	Simple Square Integral
SSC	Slope Sign Change/Slope Sign Changes
STA	Spike-Triggered Averaging
STD_DEV	Standard Deviation
STDE	Standard Deviation of the Error
STM32	STMicroelectronics 32-bit Microcontroller
SVE	Soft Voting Ensemble
SVM	Support Vector Machine
TA	Tibialis Anterior
TBW	Total Body Water
TO	Toe-Off
TTP	Total Power
TUG	Timed Up and Go
UART	Universal Asynchronous Receiver-Transmitter
*μ*V	Microvolts
US	Ultrasound/Unstable Surface
USB	Universal Serial Bus
VAR	Variance
VAS	Visual Analog Scale
Vel-AP	Velocity of center of pressure displacement in the anterior–posterior direction
Vel-ML	Velocity of center of pressure displacement in the medial–lateral direction
VL	Vastus Lateralis
VM	Vastus Medialis
WAMP	Willison Amplitude
WE	Wavelet Entropy
WL	Waveform Length/Wavelength
XGB	Extreme Gradient Boosting
YG	Young Group
ZC	Zero Crossing
