# Supplementary material for: Electrophysiological Signatures of Sarcopenia: A Systematic Review of sEMG Features, Fatigue Indices and AI-Based Classifiers
Source: Sensors (Basel). 2026 Aug 13;26(16):5121. doi: 10.3390/s26165121 (PMC13517259; doi:10.3390/s26165121)
Supplement: Supplementary file 1 [file sensors-26-05121-s001.zip › File S1.pdf]

S1: Queries of systematic searches

Table S1.1. Primary search considering research manuscripts associated to each consulted databases.

No	Data-base	Primary query (Open access, Title/Abstract)	hits	Date range	Filters applied (primary)	Fields searched	hits	Query without OA filter (Title/Abstract)	hits
1	PubMed	("healthy"[Title/Abstract] AND ("elderly"[Title/Abstract] OR "aged"[Title/Abstract] OR "aging"[Title/Abstract]) AND ("electromyography"[Title/Abstract] OR "EMG"[Title/Abstract]) AND "sarcopenia"[Title/Abstract])	24	2019/01/01 to 2026/03/31	Free full text (Open access), Humans, Publication dates 2019–2026	Titles + Abstracts	10	("healthy"[Title/Abstract] AND ("elderly"[Title/Abstract] OR "aged"[Title/Abstract] OR "aging"[Title/Abstract]) AND ("electromyography"[Title/Abstract] OR "EMG"[Title/Abstract]) AND "sarcopenia"[Title/Abstract])	12
2	Scopus	TITLE-ABS-KEY(healthy AND (elderly OR aged OR aging) AND (electromyography OR EMG) AND sarcopenia)	52	2019 – 2026	Open Access filter, Years 2019..2026, Document type: Article (optional)	Title/Abstract/Keywords	39	TITLE-ABS-KEY(healthy AND (elderly OR aged OR aging) AND (electromyography OR EMG) AND sarcopenia)	16
3	IEEE Xplore	("Healthy" AND ("Elderly" OR "Aged" OR "Aging") AND ("Electromyography" OR "EMG") AND "Sarcopenia")	4	2019 – 2026	Open Access (free) content, Year range 2019–2026	Metadata / Title & Abstract	3	("Healthy" AND ("Elderly" OR "Aged" OR "Aging") AND ("Electromyography" OR "EMG") AND "Sarcopenia")	1

Table S1.2. Queries of the Secondary search considering systematic reviews and meta-analyses

No	Database	Query (Open access, Title/Abstract) All	Hits	Date range	Filters applied	Hits	Fields searched	Query without OA filter (Title/Abstract)	Hits
1	PubMed	("healthy"[Title/Abstract] AND ("elderly"[Title/Abstract] OR "aged"[Title/Abstract] OR "aging"[Title/Abstract]) AND ("electromyography"[Title/Abstract] OR "EMG"[Title/Abstract]) AND "sarcopenia"[Title/Abstract])	11	2019/01/01 to 2026/03/31	Free full text (Open access), Humans, Publication dates 2019–2026	1	Titles + Abstracts	("healthy"[Title/Abstract] AND ("elderly"[Title/Abstract] OR "aged"[Title/Abstract] OR "aging"[Title/Abstract]) AND ("electromyography"[Title/Abstract] OR "EMG"[Title/Abstract]) AND "sarcopenia"[Title/Abstract])	1
2	Scopus	TITLE-ABS-KEY(healthy AND (elderly OR aged OR aging) AND (electromyography OR EMG) AND sarcopenia)	6	2019 – 2026	Open Access filter, Years 2019..2026, Document type: Article (optional)	4	Title + Abstract + Keywords	TITLE-ABS-KEY(healthy AND (elderly OR aged OR aging) AND (electromyography OR EMG) AND sarcopenia)	4
3	IEEE Xplore	("Healthy" AND ("Elderly" OR "Aged" OR "Aging") AND ("Electromyography" OR "EMG") AND "Sarcopenia")	0	2019 – 2026	Open Access (free) content, Year range 2019–2026	0	Metadata + Title + Abstract	("Healthy" AND ("Elderly" OR "Aged" OR "Aging") AND ("Electromyography" OR "EMG") AND "Sarcopenia")	0

Table S1.3. Queries of the Secondary search considering systematic reviews and meta-analyses of Query Elderly without Sarcopenia

No	Database	Query (Open access, Title/Abstract) All	Hits	Date range	Filters applied	Hits	Fields searched	Query without OA filter (Title/Abstract)	Hits
1	PubMed	("healthy"[Title/Abstract] AND ("elderly"[Title/Abstract] OR "aged"[Title/Abstract] OR "aging"[Title/Abstract]) AND ("electromyography"[Title/Abstract] OR "EMG"[Title/Abstract])	14	2019/01/01 to 2026/03/31	Free full text (Open access), Humans, Publication dates 2019–2026	8	Titles + Abstracts	("healthy"[Title/Abstract] AND ("elderly"[Title/Abstract] OR "aged"[Title/Abstract] OR "aging"[Title/Abstract]) AND ("electromyography"[Title/Abstract] OR "EMG"[Title/Abstract]) AND "sarcopenia"[Title/Abstract])	2

Table S1.4. Queries E of the Secondary search considering systematic reviews and meta-analyses: Elderly with Sarcopenia without Healthy keyword.

No	Database	Query (Open access, Title/Abstract) All	Hits	Date range	Filters applied	Hits	Fields searched	Query without OA filter (Title/Abstract)	Hits
1	PubMed	("elderly"[Title/Abstract] OR "aged"[Title/Abstract] OR "aging"[Title/Abstract]) AND ("electromyography"[Title/Abstract] OR "EMG"[Title/Abstract]) AND "sarcopenia"[Title/Abstract])	2	2019/01/01 to 2026/03/31	Free full text (Open access), Humans, Publication dates 2019–2026	1	Titles + Abstracts	("healthy"[Title/Abstract] AND ("elderly"[Title/Abstract] OR "aged"[Title/Abstract] OR "aging"[Title/Abstract]) AND ("electromyography"[Title/Abstract] OR "EMG"[Title/Abstract]) AND "sarcopenia"[Title/Abstract])	1

Conclusion

When considering aging with and without sarcopenia exclusively, it is observed that in the study of healthy patients or patients with other chronic morbidities has been prioritized in a 1:7 ratio. If all searches are analysed together, it is argued that the period considered represents a compromise between the quantity and the novelty of the information.

Table S1.5. Primary PubMed search query, results, time and file name.

File Name	Content	Results	Time
**PUBMED_A_FILTERCA_20260705.csv**	Filtered search results within the proposed time period	12	12:05am
**PUBMED_A_FILTERCA_OA_20260704.csv**	Result of the filtered search within the proposed open access time period	10	11:58pm
**PUBMED_A_FILTERCA_REV_20260705.csv**	Result of reviews within proposed time period	1	12:34am
**PUBMED_A_FILTERCA_REV_OA_20260705.csv**	Results of reviews within the proposed time period (OA)	1	12:34am
**PUBMED_A_ALL_20260704.png**	Search Query	24	11:44pm
**PUBMED_A_FULL_20260704.png**	Search Query+Full papers	14	11:46pm
**PUBMED_A_FILTER_20260704.png**	Search Query + Full papers + humans	11	11:48pm
**PUBMED_A_FILTER5A_20260705.png**	Search Query + Full papers + humans in 5 years	6	12:11am
**PUBMED_A_FILTERCA_20260705.png**	Search Query+Full papers + humans in the period 2019-0-01 to 2026-03-31	12	12:03am
**PUBMED_A_FILTER10A_20260705.png**	Search Query + Full papers + humans in 10 years	15	12:13am
**PUBMED_A_FILTER5A_OA_20260704.png**	Search Query+Free Full papers + humans in 5 years	5	11:51pm
**PUBMED_A_FILTERCA_OA_20260704.png**	Search Query+Free Full papers + humans in the period 2019-0-01 to 2026-03-31	10	11:49pm
**PUBMED_A_FILTER10A_OA_20260704.png**	Search Query+Free Full papers + humans in 10 years	11	11:53pm

**PUBMED_A_FILTER5A_REV_20260705.png**	Búsqueda Query+Full papers + humanos + Systematic Review or Metadatos en 5 años	0	12:24am
**PUBMED_A_FILTERCA_REV_20260705.png**	Search Query+Full papers + humans + Systematic Review or Metadata in the period 2019-0-01 to 2026-03-31	1	12:26am
**PUBMED_A_FILTER10A_REV_20260705.png**	Search Query+Full papers +humans +Systematic Review or Metadata in 10 years	1	12:15am
**PUBMED_A_FILTER5A_REV_OA_20260705.png**	Search Query + Free Full papers + human + Systematic Review or Metadata in 5 years OA	0	
**PUBMED_A_FILTERCA_REV_OA_20260705.png**	Search Query + Free Full papers + humans in + Systematic Review or Metadata in the period 2019-0-01 to 2026-03-31	1	12:31am
**PUBMED_A_FILTER10A_REV_OA_20260705.png**	Search Query Free Full papers + humans + Systematic Review or Metadata in 10 years	1	12:20

Table S1.6. Primary Scopus search query, results, time and file name.

File Name	Content	Results	Time
**SCOPUS_B_FILTERCA_20260705.csv**	Filtered search results within the proposed time period	23	1:04am
**SCOPUS_B_FILTERCA_OA_20260705.csv**	Result of the filtered search within the proposed open access time period	9	1:16am
**SCOPUS_B_FILTERCA_REV_20260705.csv**	Result of reviews within proposed time period	4	1:50am
**SCOPUS_B_FILTERCA_REV_OA_20260705.csv**	Results of reviews within the proposed time period (OA)	4	1:50am
**SCOPUS_B_ALL_20260705.png**	Search Query	52	12:46am
**SCOPUS_B_FULL_20260705.png**	Search Query+Full papers	51	12:56am
**SCOPUS_B_FILTER_20260705.png**	Search Query + Full papers + humans	39	12:59am
**SCOPUS_B_FILTER5A_20260705.png**	Search Query + Full papers + humans in 5 years	12	1:10am
**SCOPUS_B_FILTERCA_20260705.png**	Search Query+Full papers + humans in the period 2019-0-01 to 2026-03-31	22	1:02am
**SCOPUS_B_FILTER10A_20260705.png**	Search Query + Full papers + humans in 10 years	28	1:07am
**SCOPUS_B_FILTER5A_OA_20260705.png**	Search Query+Free Full papers + humans in 5 years	9	1:13am
**SCOPUS_B_FILTERCA_OA_20260705.png**	Search Query+Free Full papers + humans in the period 2019-0-01 to 2026-03-31	16	1:14am
**SCOPUS_B_FILTER10A_OA_20260705.png**	Search Query+Free Full papers + humans in 10 years	20	1:19am
**SCOPUS_B_FILTER5A_REV_20260706.png**	Búsqueda Query+Full papers + humanos + Systematic Review or Meta-Analysis en 5 años	6/15	7:51pm
**SCOPUS_B_FILTERCA_REV_20260706.png**	Search Query+Full papers + humans + Systematic Review or Metadata in the period 2019-0-01 to 2026-03-31	4/22	1:08am
**SCOPUS_B_FILTER10A_REV_20260706.png**	Search Query+Full papers +humans +Systematic Review or Metadata in 10 years	5/28	1:07am
**SCOPUS_B_FILTER5A_REV_OA_20260706.png**	Search Query + Free Full papers + human + Systematic Review or Metadata in 5 years OA	1/9	1:52am
**SCOPUS_B_FILTERCA_REV_OA_20260706.png**	Search Query + Free Full papers + humans in + Systematic Review or Metadata in the period 2019-0-01 to 2026-03-31	4/16	1:56am

**SCOPUS_B_FILTER10A_REV_OA_20260706.png**	Search Query Free Full papers + humans + Systematic Review or Metadata in 10 years	4/20	1:51am

Table S1.7. Primary IEEE search query, results, time and file name.

File Name	Content	Results	Time
**IEEE_C_FILTERCA_20260707-01.44.02.csv**	Filtered search results within the proposed time period	3	11:44pm
**IEEE_C_FILTERCA_OA_20260707-01.42.14.csv**	Result of the filtered search within the proposed open access time period	1	11:42pm
**IEEE_C_FILTERCA_REV_20260706.csv ***	Result of reviews within proposed time period	0	11:45pm
**IEEE_C_FILTERCA_REV_OA_20260706.csv***	Results of reviews within the proposed time period (OA)	0	11:45pm
**IEEE_C_ALL_20260706.png**	Search Query	4	11:26pm
**IEEE_C_FULL_20260706.png****	Search Query+Full papers	4	11:30pm
**IEEE_C_FILTER_20260706.png****	Search Query + Full papers + humans	4	11:36pm
**IEEE_C_FILTER5A_20260706.png**	Search Query + Full papers + humans in 5 years	3	11:49
**IEEE_C_FILTERCA_20260706.png**	Search Query+Full papers + humans in the period 2019-0-01 to 2026-03-31	3	11:36pm
**IEEE_C_FILTER10A_20260706.png**	Search Query + Full papers + humans in 10 years		
**IEEE_C_FILTER5A_OA_20260706.png**	Search Query+Free Full papers + humans in 5 years*	2	11:52pm
**IEEE_C_FILTERCA_OA_20260706.png**	Search Query+Free Full papers + humans in the period 2019-0-01 to 2026-03-31	1	11:39pm
**IEEE_C_FILTER10A_OA_20260707.png**	Search Query + Full papers + humans in 10 years	3	12:09am
**IEEE_C_FILTER5A_REV_20260706.png***	Search Quary+Full papers + humans + Systematic Revision or Metadata in 5 years *	0	12:18am
**IEEE_C_FILTERCA_REV_20260706.png***	Search Query+Free Full papers + humans in the period 2019-0-01 to 2026-03-31*	0	12:18am
**IEEE_C_FILTER10A_REV_20260706.png***	Search Query+Free Full papers + humans in 10 years	0	12:08am
**IEEE_C_FILTER5A_REV_OA_20260706.png***	Search Query + Free Full papers + human + Systematic Review or Metadata in 5 years OA	0	12:08am
**IEEE_C_FILTERCA_REV_OA_20260706.png***	Search Query + Free Full papers + humans in + Systematic Review or Metadata in the period 2019-0-01 to 2026-03-31*	0	12:08am
**IEEE_C_FILTER10A_REV_OA_20260706.png***	Search Query Free Full papers + humans + Systematic Review or Metadata in 10 years *	0	12:08am

*No file was generated because we do not have available information; the filters between OA and NOA were done manually. ** same information with and without OA

Table S1.8. Secondary PubMed – just elderly not sarcopenia, search query, results, time and file name.

File Name	Content	Results	Time
**PUBMED_D_FILTERCA_20260707.csv**	Filtered search results within the proposed time period	238	3:53pm
**PUBMED_D_FILTERCA_OA_20260707.csv**	Result of the filtered search within the proposed open access time period	126	4:01pm
**PUBMED_D_FILTERCA_REV_20260707.csv**	Result of reviews within proposed time period	8	4:07pm
**PUBMED_D_FILTERCA_REV_OA_20260707.csv**	Results of reviews within the proposed time period (OA)	2	5:33pm
**PUBMED_D_ALL_20260707.png**	Search Query	853	3:44pm
**PUBMED_D_FULL_20260707.png**	Search Query+Full papers	771	3:47pm
**PUBMED_D_FILTER_20260707.png**	Search Query + Full papers + humans	624	3:49pm
**PUBMED_D_FILTER5A_20260707.png**	Search Query + Full papers + humans in 5 years	151	3:50pm
**PUBMED_D_FILTERCA_20260707.png**	Search Query+Full papers + humans in the period 2019-0-01 to 2026-03-31	238	3:52pm
**PUBMED_D_FILTER10A_20260707.png**	Search Query + Full papers + humans in 10 years	308	3:55pm
**PUBMED_D_FILTER5A_OA_20260707.png**	Search Query+Free Full papers + humans in 5 years	89	3:58pm
**PUBMED_D_FILTERCA_OA_20260707.png**	Search Query+Free Full papers + humans in the period 2019-0-01 to 2026-03-31	126	4:00pm
**PUBMED_D_FILTER10A_OA_20260707.png**	Search Query + Full papers + humans in 10 years	155	3:56pm
**PUBMED_D_ALL_REV_20260707.png**	Search Query+ Systematic Review or Meta-analysis	14	8:41pm
**PUBMED_D_FILTER5A_REV_20260707.png**	Search Query + Full papers + human + Systematic Revision or Metadata in 5 years	4	4:10pm
**PUBMED_D_FILTERCA_REV_20260707.png**	Search Query+Full papers + humans + Systematic Review or Meta-analysis in the period 2019-0-01 to 2026-03-31	8	4:07pm
**PUBMED_D_FILTER10A_REV_20260707.png**	Search Query+Full papers +humans +Systematic Review or Metadata in 10 years	8	4:09pm
**PUBMED_D_FILTER5A_REV_OA_20260707.png**	Search Query + Free Full papers + human + Systematic Review or Metadata in 5 years OA	1	4:12pm
**PUBMED_D_FILTERCA_REV_OA_20260707.png**	Search Query + Free Full papers + humans in + Systematic Review or Metadata in the period 2019-0-01 to 2026-03-31	2	4:04pm
**PUBMED_D_FILTER10A_REV_OA_20260707.png**	Search Query Free Full papers + humans + Systematic Review or Metadata in 10 years	2	4:13pm

Table S1.9. PubMed -Secondary – just sarcopenia, search query, results, time and file name.

File Name	Content	Results	Time
**PUBMED_E_FILTERCA_20260707.csv**	Filtered search results within the proposed time period	25	5:57pm
**PUBMED_E_FILTERCA_OA_20260707.csv**	Result of the filtered search within the proposed open access time period	18	6:00pm
**PUBMED_E_FILTERCA_REV_20260707.csv**	Result of reviews within proposed time period	1	6:35pm
**PUBMED_E_FILTERCA_REV_OA_20260707.csv**	Results of reviews within the proposed time period (OA)	1	6:19pm
**PUBMED_E_ALL_20260707.png**	Search Query	63	3:49pm
**PUBMED_E_FULL_20260707.png**	Search Query+Full papers	60	5:50pm
**PUBMED_E_FILTER_20260707.png**	Search Query + Full papers + humans	40	5:52pm
**PUBMED_E_FILTER5A_20260707.png**	Search Query + Full papers + humans in 5 years	17	6:02pm
**PUBMED_E_FILTERCA_20260707.png**	Search Query+Full papers + humans in the period 2019-0-01 to 2026-03-31	25	5:56pm
**PUBMED_E_FILTER10A_20260707.png**	Search Query + Full papers + humans in 10 years	31	6:05pm
**PUBMED_E_FILTER5A_OA_20260707.png**	Search Query+Free Full papers + humans in 5 years	0	6:16pm
**PUBMED_E_FILTERCA_OA_20260707.png**	Search Query+Free Full papers + humans in the period 2019-0-01 to 2026-03-31	18	5:59pm
**PUBMED_E_FILTER10A_OA_20260707.png**	Search Query+Free Full papers + humans in 10 years	19	6:22pm
**PUBMED_E_ALL_REV_20260707.png**	Search Query+ Systematic Review or Meta-analysis	2	8:35pm
**PUBMED_E_FILTER5A_REV_20260707.png**	Search Query + Full papers + human + Systematic Revision or Metadata in 5 years	0	6:27pm
**PUBMED_E_FILTERCA_REV_20260707.png**	Search Query+Full papers + humans + Systematic Review or Meta-analysis in the period 2019-0-01 to 2026-03-31	1	6:30pm
**PUBMED_E_FILTER10A_REV_20260707.png**	Search Query+Full papers +humans +Systematic Review or Metadata in 10 years	1	6:07pm
**PUBMED_E_FILTER5A_REV_OA_20260707.png**	Search Query + Free Full papers + human + Systematic Review or Metadata in 5 years OA	0	6:10pm
**PUBMED_E_FILTERCA_REV_OA_20260707.png**	Search Query + Free Full papers + humans in + Systematic Review or Metadata in the period 2019-0-01 to 2026-03-31	1	6:32pm
**PUBMED_E_FILTER10A_REV_OA_20260707.png**	Search Query Free Full papers + humans + Systematic Review or Metadata in 10 years	1	6:18pm
