# Supplementary material for: Electrophysiological Signatures of Sarcopenia: A Systematic Review of sEMG Features, Fatigue Indices and AI-Based Classifiers
Source: Sensors (Basel). 2026 Aug 13;26(16):5121. doi: 10.3390/s26165121 (PMC13517259; doi:10.3390/s26165121)
Supplement: Supplementary file 1 [file sensors-26-05121-s001.zip › File S2.pdf]

S2. Reviewers' Concordance Judgment Analysis

The selection of articles was conducted using the search query in March 2026 by K.V.V.D.L and verified by B.T.C. Any disagreements were resolved by consensus among all authors. Then, in July 2026, L.I.G.J., as an independent reviewer, repeated the search using the same query and documented the entire process. After independently application of PRISMA selection criteria, authors consensus was reached for both results.

Table S2.1. Complete Table of reviewers' consensus and excluded papers, REV1 (K.V.V.D.L & B.T.C.), REV2 (L.I.G.J)

REVIEWER 1				REVIEWER 2			JUDGEMENT		INCLUSION CONSENSUS	
#	SOURCE	ID	TITLE	ID	SOURCE	TITLE	REV1	REV2	SELEC-TION	RACIONALE
1	PUBMED	15	Age- and sex-specific effects in paravertebral surface electromyographic back extensor muscle fatigue in chronic low back pain	1	PUBMED	Age- and sex-specific effects in paravertebral surface electromyographic back extensor muscle fatigue in chronic low back pain	1	1	1	
2	PUBMED	13	Age-specific differences in the time-frequency representation of surface electromyographic data recorded during a submaximal cyclic back extension exercise: a promising biomarker to detect early signs of sarcopenia	2	PUBMED	Age-specific differences in the time-frequency representation of surface electromyographic data recorded during a submaximal cyclic back extension exercise: a promising biomarker to detect early 1gns of sarcopenia	1	1	1	
3	PUBMED	11	Characteristics of the Electrophysiological Properties of Neuromuscular Motor Units and Its Adaptive Strategy Response in Lower Extremity Muscles for Seniors with Pre-Sarcopenia: A Preliminary Study	3	PUBMED	Characteristics of the Electrophysiological Properties of Neuromuscular Motor Units and Its Adaptive Strategy Response in Lower Extremity Muscles for Seniors with Pre-Sarcopenia: A Preliminary Study	1	1	1	
4	IEEE	1	Digital Biomarker for Muscle Function Assessment Using Surface Electromyography With Electrical Stimulation and a Non-Invasive Wearable Device	4	PUBMED	Digital Biomarker for Muscle Function Assessment Using Surface Electromyography With Electrical Stimulation and a Non-Invasive Wearable Device	1	1	1	

5	PUBMED	14	Effect of 6-Min Mastication Test on Masticatory Function in Young Versus Older Adults: A Comparative SEMG Study	5	PUBMED	Effect of 6-Min Mastication Test on Masticatory Function in Young Versus Older Adults: A Comparative SEMG Study	1	1	1	
6	PUBMED	9	Exploration of a machine learning approach for diagnosing sarcopenia among Chinese community-dwelling older adults using sEMG-based data	6	SCOPUS	Exploration of a machine learning approach for diagnosing sarcopenia among Chinese community-dwelling older adults using sEMG-based data	1	1	1	
7				7	SCOPUS	Fasting-Mimicking-Diet does not reduce skeletal muscle function in healthy young adults: a randomized control trial	0	1	0	It compares diets in young people
8	PUBMED	12	Lower extremity muscle activation patterns in sarcopenic older adults during physical performance tests: implications for rehabilitation approaches	8	PUBMED	Lower extremity muscle activation patterns in sarcopenic older adults during physical performance tests: implications for rehabilitation approaches	1	1	1	
9	PUBMED	5	Multi-channel EMG manifestations of upper-extremity muscle coordination imbalance among community-dwelling sarcopenic seniors	9	PUBMED	Multi-channel EMG manifestations of upper-extremity muscle coordination imbalance among community-dwelling sarcopenic seniors	1	1	1	
10	PUBMED	10	Neural control properties of the external anal sphincter in young and elderly women	10	PUBMED	Neural control properties of the external anal sphincter in young and elderly women	1	1	1	
11	PUBMED	8	Quadriceps muscle electromyography activity during physical activities and resistance exercise modes in younger and older adults	11	SCOPUS	Quadriceps muscle electromyography activity during physical activities and resistance exercise modes in younger and older adults	1	1	1	
12				12	SCOPUS	Sarcopenic obesity as a determinant of cardiovascular disease risk in older people: a systematic review	0	1	0	Another Comorbidity
13	PUBMED	4	sEMG-based Sarcopenia risk classification using empirical mode decomposition and machine learning algorithms	13	PUBMED	sEMG-based Sarcopenia risk classification using empirical mode decomposition and machine learning algorithms	1	1	1	
14				14	PUBMED	The effects of aging on the distribution and strength of correlated neural inputs to postural muscles during unperturbed bipedal stance	0	1	0	No sarcopenia

15				15	PUBMED	The influence of resistance training on neuromuscular function in middle-aged and older adults: A systematic review and meta-analysis of randomized controlled trials	0	1	0	Systematic Review
16				16	SCOPUS	The role of novel motor unit magnetic resonance imaging to investigate motor unit activity in aging skeletal muscle	0	1	0	Systematic Review
17	PUBMED	1	Are There Differences in Postural Control and Muscular Activity in Individuals with COPD and with and Without Sarcopenia?				1	0	1	With and without sarcopenia comparison
	**TOTAL**	**12**		**16**		**TOTAL**	**12**	**16**	**12**	
						**PROPORCION (Selected/Final Selection)**	**1**	**0.75**	**1**	

Kappa statistic calculation:

Data summary extracted form Table S2.1:

1. (1,1) × 11 ítems: items 1–6, 8–11, 13
2. (1,0) × 1 ítem: item 17
3. (0,1) × 5 ítems: items 7, 12, 14, 15, 16
4. (0,0) × 0 ítems

Then the contingency table 2×2 (Rater1 rows, Rater2 columns) elements are defined.

1. a = Both 1 = 11
2. b = R1=1, R2=0 = 1
3. c = R1=0, R2=1 = 5
4. d = Both 0 = 0, N = 17

Proportion according to observed Po

$Po = (a + d)/N = (11 + 0)/17 = 11/17 = 0.64705882(64.71\%)$

Marginal proportions:

$p1_{yes} = (a + b)/N = 12/17$= 0.70588235(70%)

$p1_{no} = (c + d)/N = 5/17$= 0.29411765(29%)

$p2_{yes} = (a + c)/N = 16/17$= 0.94117647(94%)

$p2_{no} = (b + d)/N = (1 + 0)/17$ =0.05882353(5%)

Agreement expected by chance Pe:

$Pe = p1_{yes} * p2_{yes} + p1_{no} * p2_{no} = (12/17)(16/17) + (5/17)(1/17) = (192 + 5)/289 = 197/289 \approx 0.68165939$ (68.17%)

Cohen's $kappa\kappa = (Po - Pe)/(1 - Pe) = (0.64705882 - 0.68165939)/(1 - 0.68165939) = -0.03460057/0.31834061 \approx -0.1086$

κ ≈ −0.11 (negative, it indicates that the agreement is less than expected by chance. According to conventional scales this would be “poor / no agreement” (or less than chance). This is the reason of using global consensus [1].

Approximated standard error, z, p, and C195 (approximations).

We used the approximation:

$$SE \approx sqrt\left(\left(Po * (1 - Po)\right)/(N * (1 - Pe)^2)\right)$$

$$Po * (1 - Po) = (0.64705882 * 0.35294118 \approx 0.228)$$

$$N * (1 - Pe)^2 = 17 * (0.31834061)^2 \approx 17 * 0.10133 \approx 1.7226$$

$$SE \approx sqrt(0.228/1.7226) \approx sqrt(0.1324) \approx 0.3639$$

$z = \kappa/SE = -0.1086/0.3639 \approx -0.299 p \approx 0.765$ non-significant.

CI 95 % (approx.) $= \kappa \pm 1.96 * SE = -0.1086 \pm 0.7131 \rightarrow -0.8217, 0.6045$

We used the approximation: $SE \approx sqrt\left(\left(Po * (1 - Po)\right)/\left(N * (1 - Pe)^2\right)\right)$

$$Po * (1 - Po) = 0.64705882 * 0.35294118 \approx 0.228 N * (1 - Pe)^2 = 17 * (0.31834061)^2 \approx 17 * 0.10133 \approx 1.7226 SE \approx sqrt(0.228/1.7226) \approx sqrt(0.1324) \approx 0.3639$$

$z = \kappa/SE = -0.1086/0.3639 \approx -0.299 p \approx 0.765$ non-significant.

CI 95 % (approx.) $= \kappa \pm 1.96 * SE = -0.1086 \pm 0.7131 \rightarrow -0.8217, 0.6045$

Discussion:

Facts κ ≈ −0.11 (95% CI ≈ −0.82 a 0.60), p ≈ 0.77. There is no evidence of agreement greater than chance.

The CI is wide because N is small and the prevalence bias is large (both reviewers marked almost always a “1”).

The κ value was negative due to the margins; they are unequal: Reviewer 2 labeled 16/17 as 1 (94%), while Reviewer 1 labeled 12/17 (71%). This marginal imbalance, and the asymmetry between the classes, produces a Pe big (≈0.682), greater than Po (≈0.647), which leads to a negative κ. Kappa penalizes heavily when one category has a high prevalence [2].

Evaluation of the two reviewers with Gwet’s AC1:

As shown in the concordance and the authors’ consensus, Reviewer 1's selection was retained. Since one category dominates, it is proposed to use Gwet’s AC1 [1].

It is considered a binary classification (Include = 1, Exclude = 0) for N = 17 papers. According to the 2×2 contingency, the following frequencies:

a = n(R1=1, R2=1) = 11, b = n(R1=1, R2=0) = 1, c = n(R1=0, R2=1) = 5,

d = n(R1=0, R2=0) = 0 and   N = a + b + c + d = 17

Proportion of observed agreement (Po)

$$Po = (a + d)/N = (11 + 0)/17 = 11/17 = 0.64705882$$

Marginal proportions per category:

For the category "Yes / Include" (i=Yes):

$p_{row-yes}$ = $p1_{yes} = (a + b)/N = 12/17 \approx 0.70588235$ (proportions that Rater1 assigned to “Yes”).

$p_{col-yes}$ = $p2_{yes} = (a + c)/N = 16/17 \approx 0.94117647$ (proportions that Rater1 assigned to “Yes”).

For the category "No / Exclude" (i=No):

p_{row-no}= $p1_{no} = (c + d)/N = 5/17 \approx 0.29411765$

p_{col-no} = $p2_{no} = (b + d)/N = 1/17 \approx 0.05882353$i

Calculation of the average marginal probabilities $\bar{p_i}$ (average of rows and columns3)

$\bar{p}_{yes}$ =$\left(p1_{yes} + p2_{yes}\right)/2$

$$= (12/17 + 16/17)/2 = (28/17)/2 = 14/17 \approx 0.82352941$$

$\bar{p}_{no} = (p1_{no} + p2_{no})/2$

$$= (5/17 + 1/17)/2 = (6/17)/2 = 3/17 \approx 0.17647059$$

Calculating the expected probability of agreement according to AC1 (Pe_{AC1})

- Pe_{AC1} = $\sum_i \bar{p_i}^2 = \bar{p_y}^2 + \bar{p}_{no}{}^2 = (14/17)^2 + \ (14/17)^2 = 205\ /\ 289\ \approx\ 0.7090$

Calculate Gwet's AC1

Formula: $AC1 = (Po - Pe_A C1)/(1 - Pe_A C1)$

Converting Po a fraction over 289 to operate consistently:

$$Po = 11/17 = 187/289 \approx 0.6471$$

$Po - Pe_{AC1} = 187/289 - 205/289 = -18/289 \approx -0.0623$

$1 - Pe_{AC1} = 1 - 205/289 = 84/289 \approx 0.2907$

$$AC1 = (-18/289)/(84/289) = -18/84 = -0.214285714$$

Interpretation:

Considering that AC1 can vary between −1 and 1. A negative AC1 indicates that the observed agreement (Po) is less than the probability of expected agreement by chance according to the estimated AC1; that means that the reviewers agree less than the model's predicted agreement by chance (AC1). The magnitude ≈ −0.21indicates "disagreement worse than chance" or, in practical terms a very bad concordance.

Comparison among metrics

When considering the results with Cohen's κ (previously calculated): κ ≈ −0.11. In this dataset, AC1 results are even more negative (based on the averages p̄_i), giving an expected agreement value even greater than that used by κ in this particular case: Pe_AC1 = 205/289 ≈ 0.709, Pe_kappa = 197/289 ≈ 0.682. Then, Po (≈0.647) remains below both, and when comparing it with a Pe greater than AC1, the results are even more negative.

Verification of the previous computation and consideration of the small sample size were done with the script "*ConcordanceMetrics.m*" in MATLAB 2024b, available on GitHub.

Bootstrap CI kappa (95%): **[-0.2830, 0.0000]** and Bootstrap CI AC1 (95%): **[-0.4167, -0.0625]**

Practical conclusion: There is evidence of systematic disagreement between the two reviewers (not mere chance) — especially because one of the reviewers (Rater2) labeled almost everything as "Include" (16/17), generating margin bias and frequent discrepancies (5 cases R1=0 vs R2=1), which is why the consensus stage was used.

Table S2.2. Comparison of reviewer performance in article selection after applying the proposed queries and filters.

Num	REV1	REV2	Source	Title
1	1	1	PUBMED	Age- and sex-specific effects in paravertebral surface electromyographic back extensor muscle fatigue in chronic low back pain
2	1	1	PUBMED	Age-specific differences in the time-frequency representation of surface electromyographic data recorded during a submaximal cyclic back exten1on exercise: a promi1ng biomarker to detect early 1gns of sarcopenia
3	1	1	PUBMED	Characteristics of the Electrophy1ological Properties of Neuromuscular Motor Units and Its Adaptive Strategy Response in Lower Extremity Muscles for Seniors with Pre-Sarcopenia: A Preliminary Study
4	1	1	PUBMED	Digital Biomarker for Muscle Function Assessment Using Surface Electromyography With Electrical Stimulation and a Non-Invasive Wearable Device
5	1	1	PUBMED	Effect of 6-Min Mastication Test on Masticatory Function in Young Versus Older Adults: A Comparative SEMG Study
6	0	1	SCOPUS	Effectiveness of a cardiac rehabilitation program on biomechanical, imaging, and physiological biomarkers in elderly patients with heart failure with preserved ejection fraction (HFpEF): FUNNEL + study protocol
7	0	1	IEEE	Effects of Lower Limb Muscle Activities to Osteoarthritic Knees
8	0	1	SCOPUS	Epidemiology and effect on physical function of osteosarcopenia in community-dwelling elderly people in Japan
9	1	1	SCOPUS	Exploration of a machine learning approach for diagnosing sarcopenia among Chinese community-dwelling older adults using sEMG-based data

10	0	1	SCOPUS	Fasting-Mimicking-Diet does not reduce skeletal muscle function in healthy young adults: a randomized control trial
11	0	1	PUBMED	High-density Surface Electromyography as Biomarker of Muscle Aging
12	1	1	PUBMED	Lower extremity muscle activation patterns in sarcopenic older adults during physical performance tests: implications for rehabilitation approaches
13	1	1	PUBMED	Multi-channel EMG manifestations of upper-extremity muscle coordination imbalance among community-dwelling sarcopenic seniors
14	0	1	SCOPUS	Muscle activation of lower limb during walking in elderly individuals with sarcopenia: A pilot study
15	1	1	PUBMED	Neural control properties of the external anal sphincter in young and elderly women
16	1	1	SCOPUS	Quadriceps muscle electromyography activity during physical activities and resistance exercise modes in younger and older adults
17	0	1	SCOPUS	Sarcopenic obesity as a determinant of cardiovascular disease risk in older people: a systematic review
18	1	1	PUBMED	sEMG-based Sarcopenia risk classification using empirical mode decomposition and machine learning algorithms
19	0	1	PUBMED	The effects of aging on the distribution and strength of correlated neural inputs to postural muscles during unperturbed bipedal stance
20	1	1	PUBMED	The influence of resistance training on neuromuscular function in middle-aged and older adults: A systematic review and meta-analysis of randomised controlled trials
21	1	1	SCOPUS	The role of novel motor unit magnetic resonance imaging to investigate motor unit activity in ageing skeletal muscle
22	1	0	IEEE	Are There Differences in Postural Control and Muscular Activity in Individuals with COPD, with and without Sarcopenia?
**Concordance**	**1**	**0.95**	**Selected by both reviewers**	
**Non selected**	**0.36**	**0.05**	**Not selected originally before consensus**	

As evidenced by the agreement and the consensus among authors, almost all of Reviewer 1's selection was maintained. This was due to its origin in an independent analysis of query application across databases at different times. Differences in the handling and criteria available in the applied filters also contributed to this. Some criteria were manually applied, particularly in Scopus and IEEE Xplore. The main difference lay in the discrimination criteria regarding relevance to the topic of interest. Therefore, we consider this metric inadequate for expressing consensus. A consensus round was conducted among the authors, allowing us to refine our selection and define the 12 articles included in the analysis, as shown in Table S2.2.

Importantly, despite differences in intermediate counts caused by database and manual filtering idiosyncrasies, the final set of included articles, those that meet the criterion of allowing performance comparison between older adults with and without sarcopenia, was validated through independent review and group consensus.

Conclusion

Inter-rater agreement was assessed using Cohen’s kappa (κ) (Cohen, 1960). We report κ with 95% CI and raw agreement and interpret κ following Landis & Koch (1977). Because κ can be unstable with extreme prevalence or rater bias, we also present raw agreement and AC1 and discuss limitations (Viera & Garrett, 2005; McHugh, 2012). A third round of all authors' analysis was performed for final article selection with a total consensus.

References.

[1] Cohen, J. (1960). A coefficient of agreement for nominal scales. *Educational and psychological measurement*, *20*(1), 37-46.

[2] McHugh, M. L. (2012). Interrater reliability: the kappa statistic. *Biochemia medica*, *22*(3), 276-282.

[3] Gwet, K. L. (2008). Computing inter-rater reliability and its variance in the presence of high agreement. *British Journal of Mathematical and Statistical Psychology*, *61*(1), 29-48.

[4] Sim, J., & Wright, C. C. (2005). The kappa statistic in reliability studies: use, interpretation, and sample size requirements. *Physical therapy*, *85*(3), 257-268.
