# Supplementary material for: Electrophysiological Signatures of Sarcopenia: A Systematic Review of sEMG Features, Fatigue Indices and AI-Based Classifiers
Source: Sensors (Basel). 2026 Aug 13;26(16):5121. doi: 10.3390/s26165121 (PMC13517259; doi:10.3390/s26165121)
Supplement: Supplementary file 1 [file sensors-26-05121-s001.zip › File S3.pdf]

S3. Risk Analysis

This document presents the detailed analysis of the papers considered in the systematic review. First, the GRADE summary traffic light plot presented in the main text is supported by Table S3.1. This evaluation was verified using the RoB proposed in Table S3.2, according to the study type. The results of these evaluation techniques are summarized in Table S3.3. Besides, on GitHub, a folder is included with the specific results by paper. Then, a discussion and conclusion that support the changes in figures 2 an 3 of the main text. Finally, the references used for each technique application are included.

Table S3.1. Detailed GRADE assignment of risk levels.

Source / Reference / Group	*Random sequence generation (selection bias)*	*Allocation concealment (selection bias)*	*Blinding of participants and personnel (performance bias)*	*Blinding of outcome assessment (detection bias)*	*Incomplete outcome data (attrition bias)*	*Selective reporting (reporting bias)*	*Other bias*
IEEE 1 / (Song et al. 2024) / C	**Low**<br>The study implements leave-one-subject-out cross-validation (LOSO-CV), which is methodologically robust because it ensures that the training and test sets do not contain samples from the same subject. This reduces intra-subject selection bias, which is critical in signal studies.	**Low**<br>Data partitioning into training and test is automatic and defined by the LOSO scheme, avoiding manual intervention. There is no evidence of data-splitting manipulation to favor model performance.	**Low**<br>Participants do not change their behavior in response to the model, since EMG acquisition is independent of the algorithm's training. There is no behavioral intervention during modeling.	**Low**<br>The outcome (classification) is generated automatically by the model. There is no human intervention in assigning predicted labels. Additionally, metrics (accuracy, F1) are calculated deterministically.	**Low**<br>No exclusions of subjects or selective deletion of data are reported. All records appear included in validation.	**Low**<br>Multiple metrics are reported (accuracy, precision, recall, F1), which reduces the risk of reporting only favorable results.	**Unclear**<br>There is a significant risk of structural overfitting due to High feature dimensionality (EMD, time–frequency), dataset dependence because the model is trained on a single cohort, and a lack of external validation, which limits clinical generalization.
PUBMED1 / (Sepulveda et al., 2025) / A	**Unclear**<br>The study compares predefined groups (e.g., COPD with/without sarcopenia vs	**Unclear**<br>There is no group assignment, but no procedure is described to prevent	**Unclear**<br>Tasks (e.g., balance, TUG, postural conditions) are explicit and	**Unclear**<br>Although the variables (EMG RMS, COP) are objective, the study does not state	**Low**<br>No participant losses or exclusions after	**Low**<br>Variables described in methods (EMG, COP, test	**High**<br>COPD can affect both postural control and muscular/ventilatory function, making it

	controls) without random assignment. There is no random sequence generation process because the design is observational. This introduces potential selection bias, especially because group membership depends on pre-existing clinical conditions.	investigators from influencing participant inclusion. The absence of concealment or blind recruitment may introduce bias in group composition.	require active participation. Subjects know the type of test and how it is administered, and experimenters supervise directly. This may influence effort, motor strategy, and recorded EMG activation.	whether data processing and analysis were performed in a blinded manner to group. Decisions such as window selection, filtering, or artifact exclusion may require intervention.	recruitment that affect analysis are reported. Data appear complete for main variables.	conditions) match those reported in results, with no evidence of selective omission.	difficult to attribute EMG changes exclusively to sarcopenia.
PUBMED 4 /(Kumar et al. 2024) / C	**Low** The study uses k-fold cross-validation, which evaluates the model across multiple partitions of the dataset and reduces bias associated with a single train/test split.	**Low** Partitioning is automated; there is no evidence of manual intervention in fold selection.	**Low** Participant behavior does not affect model training, since the analysis is performed offline on previously acquired signals.	**Low** Model predictions are objective. However, if labels (sarcopenia vs. healthy) are based on predefined clinical criteria, the model inherits any bias in those labels.	**Low** No significant exclusions are reported.	**Low** Multiple performance metrics are presented.	**Unclear** Limited sample size, possible data leakage if windows from the same signal (subject) fall into different folds, absence of external validation.
PUBMED 5 / [He, et al., 2024)/ D	**Unclear** The study compares groups (young, older adults, sarcopenia risk) without random assignment. Group membership depends on age/clinical condition, introducing structural selection bias. Also, it is not described whether participants were recruited consecutively.	**Unclear** There is no experimental assignment or concealment of group. The investigator knows participant condition during acquisition, which can influence protocol conduct (e.g., motivation during MVC or effort control).	**Unclear** The participant is aware of the effort level (e.g., 50% MVC).	**High** EMG decomposition into motor units depends on algorithms (e.g., convolutive blind source separation). There is manual or semi-automatic intervention in spike validation, removal of Low - quality units, and selection of stable segments.	**Low** No losses are reported, but it is important to note that…	**Low** Multiple variables are reported (firing rate, recruitment threshold, slope, etc.), suggesting transparency.	**Unclear** Detection bias of muscles (only detectable ones are analyzed), dependence on the isometric task (50% MVC), intramuscular variability, and a non-linear relation between firing rate and force.

PUBMED 8 / Marsall et al., 2020 / A	**Low**<br>The study uses an intra-subject design where each participant performs all experimental conditions (e.g., gait, sit-to-stand). This removes the need for between-group assignment and reduces selection bias between conditions.	**Low**<br>Since there is no assignment to independent groups but only within-subject comparisons, the risk of assignment manipulation is practically nonexistent.	**High**<br>Participants are aware of the functional tasks they perform (standing up, walking), which can modify the intensity of effort, speed, or motor strategy. Additionally, staff guide execution, introducing experimenter-dependent variability.	**Low**<br>EMG variables (e.g., RMS, activation patterns) are obtained by automated processing. Probability of subjective influence in measurement is **Low**, provided the pipeline is predefined.	**Low**<br>No dropouts or relevant exclusions are reported; data appear complete.	**Low**<br>Consistency exists between objectives, measured variables and reported results.	**Low**<br>Within-subject design reduces inter-individual variability. However, variability due to accumulated fatigue or learning may persist if order is not fully counterbalanced (not clearly reported).
PUBMED 9 / (Li et al., 2024) /C	**Low**<br>The regression model (continuous biomarker) is trained using defined partitions (e.g., cross-validation), allowing consistency evaluation.	**Low**<br>Training/validation is automatic, without manual intervention in data assignment.	**Low**<br>Data acquisition (e.g., signals evoked by electrical stimulation) is independent of the model. There is no participant influence on model outcome.	**Low**<br>The output is a continuous estimate (biomarker) evaluated using Pearson correlation, mean error, and Bland–Altman analysis.	**Low**<br>No data losses are reported.	**Low**<br>No data losses are reported.	**Unclear**<br>Particular risks: dependence on the electrical stimulation protocol, model trained on a healthy population, indirect definition of the outcome, possible target-construction bias (force/mass as proxy).
PUBMED 10 / (Dias et al. 2019) / D	**Low**<br>Groups are defined by clinical criteria (AWGS), with clear classification between healthy and sarcopenic. Although not randomized, group definition is	**Unclear**<br>No concealment in classification or analysis is reported, which could influence decisions during signal processing.	**Unclear**<br>The participant controls effort in handgrip (20% and 50% MVC), introducing variability in muscle load distribution, contraction	**Unclear**<br>Although ICDMC index calculation is mathematical, prior decisions may introduce bias in channel selection, feature normalization, and reference-pattern	**Low**<br>No losses are reported.	**Low**<br>Multiple features are reported (RMS, MAV, WL, SSC), not just the most favorable ones.	**Unclear**<br>The ICDMC index is not a clinically validated biomarker but a geometric construct. Interpretation of "greater symmetry = worse function" depends on

	consistent and standardized.		stability, and coactivation.	definition (hexagon).			physiological assumptions. High dependence on electrode placement and signal normalization.
PUBMED 11 / (Hu et al., 2021) / D	**Low** The study uses controlled experimental conditions (defined torque levels), reducing systematic variability within the protocol.	**Unclear** There is no concealment in condition assignment or participant selection.	**Unclear** Participant controls effort (pushing/holding), which directly affects generated torque and impedance response.	**Low** Variables (resistance, reactance, characteristic frequency) are obtained from direct instrumental measurements. Torque estimation via mathematical models is objective.	**Low** No losses are reported.	**Low** Correlations, errors, and complete analyses are reported.	**High** Small sample, subjects in "pre-sarcopenia" validation.
PUBMED 12 / (Cai et al., 2025) / A	**Low** The study mentions randomization of exercise order, which is relevant because it controls fatigue, learning, or adaptation effects between conditions.	**Unclear** Although the order is random, it is not specified whether the process was concealed (e.g., computational randomization vs. experimenter decision), leaving uncertainty about possible intervention in the order assignment.	**High** Physical exercise tasks are evident to the participant, and staff interacts directly during execution. This can influence effort intensity and recorded EMG activation.	**Low** EMG analysis (amplitude, %MVC, etc.) is quantitative and typically automated, reducing the probability of measurement bias.	**Low** No significant losses or exclusions affecting analysis are reported.	**Low** Reported metrics correspond to those planned in the experimental design.	**Low** Groups are comparable, and protocol is controlled. However, as in other exercise studies, inter-individual variability in voluntary effort may persist, though it does not appear to systematically bias results.
PUBMED 13 / (Habenicht et al, 2020) / B	**Low** The experimental protocol uses structured fatigue conditions (e.g., sustained or repetitive	**Unclear** No formal procedure for concealing the order of conditions or	**Unclear** Participants are aware they are performing fatiguing tasks (e.g., sustained	**Low** The main metrics (MF, MDF slope, RMS) are calculated using automated spectral	**Low** No dropouts are reported during	**Low** Both amplitude and spectral variables are reported,	**Low** The fatigue protocol is relatively controlled (same relative intensity, same duration),

	contractions with defined loads), and execution order is controlled. Standardization reduces systematic variability between participants.	timing of load application is described. This is relevant because prior knowledge of the effort can modify participant anticipation and recruitment strategy.	contractions at %MVC). This directly influences voluntary effort level, recruitment strategy (compensation, activation) and tolerance to pain/fatigue.	processing. The fatigue slope is obtained from linear regression over temporal windows, thereby reducing the need for subjective intervention; risk would increase only if window choices were selective.	fatigue protocols, which is notable because such studies often have dropout risk due to exhaustion. Data completeness suggests good tolerance to the protocol.	including expected and non-significant results. There is no evidence of omission of key fatigue metrics.	reducing variability. However, physiological individual variability in fatigue resistance remains, but it does not seem to introduce systematic bias between groups.
PUBMED 14 / (Chilwan et al., 2025) /B	**Unclear** Fatigue analysis in masticatory muscles without random assignment. Order and exposure are not rigorously controlled, introducing variability in EMG response.	**Unclear** No mechanism is described to prevent investigator influence in participant inclusion or evaluation. This is relevant in small or clinical samples.	**Unclear** In tasks like chewing or voluntary contraction, the participant directly controls applied force, introducing variability in actual vs expected intensity, execution rhythm, and adaptation during the task.	**Low** EMG variables (median frequency, spectral power) are obtained via digital analysis. However, there may be intervention in segment selection, although not explicitly reported.	**Low** No evidence of losses or exclusions after start.	**Low** Expected fatigue metrics (e.g., spectral shift) are reported.	**Unclear** High biomechanical and functional variability in masticatory muscle, difficulty standardizing contraction force, influence of factors such as dental occlusion, pain, or chewing habit.
PUBMED 15 / (Ebenbichler et al., 2020) / B	**Low** The experimental protocol is well structured, with defined fatigue	**Unclear** It is not specified whether condition order was concealed from	**Unclear** The subject knows when they are fatiguing, can modulate effort to	**Low** Fatigue analysis is based on objective spectral metrics (MF, MDF, slopes),	**Low** No dropouts or relevant exclusions	**Low** Both significant and non-significant	**Low** Protocol shows good reproducibility. However, as in all fatigue studies,

conditions (e.g., % MVC, duration). In many cases, execution order is controlled or counterbalanced, reducing learning effects or accumulated fatigue.	the experimenter or participant. This may influence anticipation of effort.	sustain the task, or may stop before physiological limit.	calculated automatically. Human intervention in final quantification is minimal.	are reported, important for fatiguing protocols.	results are reported, including comparisons between conditions.	inter-individual variability in resistance exists, depending on actual activation level (%MVC vs real).

Table S3.2. RoB proposed supporting the GRADE decision based on the study type.

No.	***Key signals from abstract and title: Primary studies***	***Study type***	***Quality tool***	
			1º	2º
1	PubMed (Ebenbichler et al., 2020), Age- and sex-specific effects in paravertebral SEMG back extensor fatigue in cLBP. Cross-sectional comparative + reliability study: Comparison of younger vs older groups; single-measurement sessions with repeated tests (1–2 days, 6 weeks) to assess test–retest reliability; N large	Cross-sectional with measurement properties component.	COSMIN (reliability + measurement error)	AXIS (cross-sectional design quality)
2	PubMed (Habenicht et al, 2020): Age-specific differences in time-frequency SEMG (IMDF) during cyclic back extension. Cross-sectional comparative + reliability study. Healthy volunteers grouped by age; repeated tests at two days and six weeks; linear mixed models and Generalizability Theory for reliability.	Cross-sectional comparative + reliability study for measurement properties emphasis	COSMIN (reliability + measurement properties)	AXIS (cross-sectional design/sampling & confounding)
3	PubMed (Hu et al., 2021): Characteristics of electrophysiological properties of motor units in pre-sarcopenia. Small experimental/comparative (cross-sectional) pilot study. Small groups (risk-sarcopenia, non-sarcopenia, young); single session knee extension with MU decomposition.	Cross-sectional, exploratory, pilot.	AXIS (cross-sectional study quality)	COSMIN (if assessing MU metrics reliability)

4	PubMed (Song et al. 2024): Digital biomarker for muscle function using sEMG+stimulation + wearable Development + validation (regression model) — predictive model study Model development using SMCS features + CNN + MLP regression on 98 participants; correlation with reference (BIA/strength)	Predictive model study	1º PROBAST (prediction model risk of bias)	2º TRIPOD reporting checklist (reporting/transparency)
5	PubMed (Chilwan et al., 2025): Effect of 6-min mastication test: comparative sEMG study Cross-sectional comparative (task study) Two groups (young vs older) measured during 6-min chewing test; within-group changes and between-group comparisons	cross-sectional with fatigue metrics.	1º AXIS	2º COSMIN (if reporting test–retest reliability metrics)
6	Scopus (Li et al., 2024): Exploration of a machine learning approach for diagnosing sarcopenia (sEMG + ML) Prediction/classification (ML development + CV) ML classifiers trained on features, five-fold CV reported, community sample, reporting accuracy/sensitivity	model development/validation	1º PROBAST (model development + validation quality)	2º TRIPOD / check for external validation (if present) and reporting of data leakage
7	PubMed (Cai et al., 2025): Lower extremity activation patterns in sarcopenic older adults during performance tests Cross-sectional comparative (case vs control) Comparison between sarcopenic and age-matched healthy older adults during functional tests (6MWT, 5STS); small groups (n=8) → cross-sectional case–control like.	Cross-sectional case–control like.	AXIS (cross - sectional)	NOS (adapted for small case-control comparatives) for comparability/confounding
8	PubMed ((He, et al., 2024): Multi-channel EMG manifestations of upper-extremity coordination imbalance Cross-sectional comparative (biomarker discovery) 98 healthy vs 55 sarcopenic; proposed index (ICDMC); group comparisons and stratifications	cross-sectional diagnostic/biomarker study.	AXIS (cross - sectional quality)	QUADAS-2 (if index intended as diagnostic test; assess applicability)
9	PubMed (Dias et al. 2019): Neural control properties of the external anal sphincter (HD-sEMG) Cross-sectional physiological study / measurement study Young vs elderly healthy women (n small); HD-sEMG decomposition, MU metrics, statistical comparison	physiological cross-sectional + measurement.	COSMIN (measurement properties, decomposition validity)	AXIS (cross-sectional sampling & external validity)
10	Scopus (Marsall et al.): Quadriceps EMG during activities and RET in younger vs older adults Cross-sectional comparative (within-subject tasks) Comparison young vs old across tasks/exercise modes; repeated sessions on separate days	cross-sectional comparative experimental tasks.	1º AXIS	2º COSMIN (if reporting reliability of EMG measures across days)

11	PubMed (Kumar et al. 2024): sEMG-based sarcopenia risk classification using EMD + ML Prediction/classification (ML study) Model building using feature extraction (EMD) and ML classifiers with LOSO-CV; reports accuracies per task.	ML diagnostic/prediction study.	PROBAST (prediction model risk of bias)	TRIPOD / checklist for ML reporting; check for data leakage and subject-wise split
12	IEEE (Sepulveda et al., 2025): Postural control & muscular activity in COPD with/without sarcopenia Cross-sectional diagnostic/comparative study (clinical groups) Thirty-five patients classified by EWGSOP; balance tests + sEMG; between-group comparisons	cross-sectional diagnostic/comparative clinical study.	1º QUADAS-2 (if treating as diagnostic accuracy / clinical applicability)	2º AXIS (cross-sectional quality) / NOS (if treated as cohort-like clinical comparison)

Table S3.3. Consensus evaluation from each RoB evaluation, based on two revisions (B.T.C. & L.I.G.J.)

Source / paper / Group	***Title***	***Study type***	***1ºQuality Tool / Risk definition***	***1° Rationale***	***2º Quality Tool / Risk definition***	***2° Rationale***
PUBMED 15 / (Ebenbichler et al., 2020) / B	Age- and sex-specific effects in paravertebral surface electromyographic back extensor muscle fatigue in chronic low back pain	Cross-sectional with measurement properties component.	**COSMIN** (Reliability + Measurement error) **Consensus_judgment: Overall:** Low risk, Some concerns	**Strengths:**Robust design and reporting for instrumentation and relative reliability (Low risk) **Limitations:** for absolute measurement error and incomplete reporting of exclusions/reasons (Some concerns).	**AXIS** (cross-sectional design quality)	**Strengths:** it is methodologically strong for its aims: clearly stated objectives (Intro p3), robust instrumentation and processing (Instrumentation & Signal processing pp5–7), appropriate and justified sample size (Monte Carlo simulation p7) and rigorous statistical handling (mixed models, Bonferroni, effect sizes p7). Reliability evaluated with G-Theory and ICC/SEM reported (Table4 p12). Main limitations are clinic-based recruitment (Methods p4), which limits generalizability, and a lack of formal non-responder characterization; recommend inclusion with low risk of bias in

						outcomes but note limited external generalizability.
PUBMED 13 / (Habenicht et al, 2020) / B	Age-specific differences in the time-frequency representation of surface electromyographic data recorded during a submaximal cyclic back extension exercise: a promising biomarker to detect early signs of sarcopenia	Cross-sectional comparative + reliability study for measurement properties emphasis	**COSMIN** (reliability + measurement properties). **Overall: low risk/ some concerns**	**Strength:** Objective and population clearly stated; sample size specified; and Instrumentation and signal processing thoroughly described and replicable. Design and reporting for relative reliability robust; D/ICC values presented. Appropriate statistical methods and corrections used; power considerations documented. **Some concerns**: SEM reported but SDC/MCID not provided; absolute error limits clinical interpretability. Exclusions and reasons are presented graphically, but detailed counts by reason and imputation methodology require explicit tabulation; only partially documented. No intervention implemented; domain not applicable. SEM reported; clinical thresholds (SDC/MCID) not provided — limits interpretability.	**AXIS** (cross-sectional design / sampling & confounding) **Overall, low to moderate risk of bias, with methodologically reliable results for the stated objectives.**	**Strengths: C**larity of objectives, study design, definition and measurement of variables, reproducibility of methods, statistical analysis, transparency, and ethical considerations. **Limitations** are concentrated in external validity, due to the use of convenience sampling and limited representativeness of the target population, as well as the exclusion of a relevant proportion of participants because of technical problems with the electromyographic signals. However, these exclusions were documented and analyzed, which reduces their impact on internal validity.

PUBMED 11 / (Hu et al., 2021) / D	Characteristics of the Electrophysiological Properties of Neuromuscular Motor Units and Its Adaptive Strategy Response in Lower Extremity Muscles for Seniors with Pre-Sarcopenia: A Preliminary Study	Cross-sectional, exploratory, pilot.	**AXIS** (cross-sectional study quality) **Overall:** Moderate risk	Its main strengths are the clear definition of objectives, the use of a standardized high-density surface electromyography (HD-sEMG) methodology, appropriate operationalization of variables, and adequate statistical analysis. Limitations: Reporting of the sampling process: the sampling frame and recruitment method are not described, there is no information about non-respondents or losses, and no prior sample size calculation is presented. These limitations mainly affect external validity and the ability to assess the risk of selection bias, although the study's internal validity remains acceptable due to the quality of the measurements and analysis.	**COSMIN** (if assessing MU metrics reliability) **Overall judgment on the suitability of the measurement properties:** Inadequate.	Several COSMIN domains are not applicable because the study evaluates physiological variables rather than a PROM; nevertheless, for the relevant properties (reliability, measurement error, construct validity), the evidence is insufficient per COSMIN criteria. The authors acknowledge limitations (small sample, cross-sectional design). It is insufficient for establishing measurement properties per COSMIN standards.
IEEE 1 / (Song et al. 2024) / C	Digital Biomarker for Muscle Function Assessment Using Surface Electromyography With Electrical Stimulation and a Non-Invasive Wearable Device	Predictive model study	**PROBAST** (prediction model risk of bias) **Bias Risk** (Methodological quality): Moderate / Some concerns. **Clinical use:** Moderate concerns**.** **Final rating =** "High risk of bias"	Bias risk due to complex model vs. limited sample size, lack of external validation, and ambiguity in feature selection (potential leakage). Applicability concern because the healthy cohort is aged 20–60; the primary clinical objective is to detect/monitor sarcopenia (more prevalent in those over 60 and frail patients). Therefore, direct applicability to the elderly clinical population is limited without evaluation in that specific population.	**TRIPOD** reporting checklist (reporting / transparency) **Overall Judgment (Interpretation): Adequate but with significant shortcomings (66.7%)**	**Strengths:** The article clearly describes the motivation, population, signal acquisition methodology, feature extraction, and model architecture, and presents robust performance metrics (r, ME, Bland–Altman). **Limitations:** It lacks key elements for reproducibility and external evaluation: the period of data collection is not reported, no external validation is performed, code and data are not made public, and the complete serializable version of the model and full training parameters (learning rate, optimizer, batch size, seed) are not provided. Confidence intervals for performance metrics are also missing. Additionally, it does not include a formal analysis of clinical utility

						(decision curve) or a multicenter validation plan. These limitations reduce the possibility of replication and the confidence for immediate clinical adoption.
PUBMED 14 / (Chilwan et al., 2025) /B	Effect of 6-Min Mastication Test on Masticatory Function in Young Versus Older Adults: A Comparative SEMG Study	cross-sectional with fatigue metrics.	**AXIS, Reporting quality:** Moderate.	**Overall:** Results support an age-related decline in masseter SEMG parameters and fatigue effects in the submental muscle, but findings are exploratory; replication with diagnostic-grade acquisition and larger, representative samples is recommended before clinical translation. **Strengths:** Clear objectives, detailed task protocol (6MMT cadence, gum, electrode placement, posture), appropriate statistical tests, effect sizes reported, ethics and funding declared (Methods & Results pp.2–6; Tables). **Main limitations impacting AXIS appraisal:** no a priori sample size calculation (item 3), small convenience sample from a single center (items 5–6), no recruitment/response flow (items 7,13,14), use of a therapeutic SEMG device with undisclosed acquisition specs (sampling rate, etc.) which limits reproducibility and signal-quality assessment (items 9,11, Limitations p.5). Authors acknowledge many of these limitations.	**COSMIN** (if reporting test–retest reliability metrics) **overall risk:** inadequate / non applicability	**Strengths**: The article stands out for the clarity of its objectives, methodological justification, and statistical analysis. **Limitations:** It suffers from limitations in sample planning, instrument validation, and depth of reliability and generalizability analysis.

PUBMED 9 / (Li et al., 2024) /C	Exploration of a machine learning approach for diagnosing sarcopenia among Chinese community-dwelling older adults using sEMG-based data	model development/validation	**PROBAST** (model development + validation quality) **Overall risk of bias:** Moderate / Some concerns leaning toward high risk. **Clinical use:** Some concerns	**Strengths** — clear protocol, objective outcome (AWGS criteria), well-described feature extraction, balanced internal CV, and interpretability via SHAP. **Main concerns** — potential optimistic bias because channel/feature selection appears to have been done prior to cross-validation (unclear nesting), modest final sample size for ML (n=93), exclusion of 64 borderline participants reduces generalizability, and no external validation. These issues raise concern that reported performance (accuracy ~0.73, sensitivity ~0.79; AUC 0.78) may be optimistic when applied prospectively or in other communities. **Applicability:** The study population (community-dwelling elderly in Chengdu) is appropriate for community screening. However, exclusion of borderline participants (n=64) limits applicability for screening where borderline cases are common. No testing in independent external cohorts; ethnic/geographic generalizability not demonstrated.	**TRIPOD** / check for external validation (if present) and reporting of data leakage **Low Risk of bias.** It is partially adherent to TRIPOD	**Strengths:**The study design, population, predictors, outcomes, internal validation, and discrimination metrics clearly described (adhering to many TRIPOD items). **Limitations:** Lack of formal sample size justification, incomplete handling/reporting of missing data, absence of confidence intervals or uncertainty measures for performance metrics and no calibration assessment, no external validation, and limited reproducibility artifacts (no public code, model coefficients, or serialized models). Data are available upon request but not openly shared. These shortcomings limit transparency and reproducibility in accordance with TRIPOD.

PUBMED 12 / (Cai et al., 2025) / A	Lower extremity muscle activation patterns in sarcopenic older adults during physical performance tests: implications for rehabilitation approaches	Cross-sectional case–control like.	**AXIS** (cross-sectional) **Overall risk:** moderate	**Strengths** —The evaluation using the AXIS tool shows that strengths include the use of a highly standardized experimental protocol, the employment of validated instruments (Vicon, AMTI, and Delsys Trigno), a clear definition of variables, and appropriate statistical analysis. The **main limitations** relate to external validity: no sample size calculation is presented, the sample size is small, the recruitment process is insufficiently described, and there is no information on non-respondents. Taken together, these aspects suggest a moderate overall risk of bias, although the study's internal validity is considered strong owing to the quality of the protocol and measurements.	NOS (adapted for small case-control comparatives) for comparability / confounding **Consensus judgment (final):** 6/9 stars — Fair quality (moderate risk of bias).	**Strengths:** Objective, standardized case definition and high-quality, identical exposure measurement (EMG and biomechanical instrumentation). Limitations: Non-representative volunteer recruitment (posters), very small sample size (n=16) with acknowledged gender imbalance and no multivariable confounder control, and absent reporting of non-response/enrollment flow. These limitations increase selection and confounding risk despite rigorous measurement methods.
PUBMED 5 / (He, et al., 2024)/ D	Multi-channel EMG manifestations of upper-extremity muscle coordination imbalance among community-dwelling sarcopenic seniors	cross-sectional diagnostic/ biomarker study.	**AXIS** (cross-sectional quality) **Overall reporting and methodological quality:** Good for a cross-sectional diagnostic/biomarker study.	**Strengths:** clear objectives; a well-described acquisition and signal-processing pipeline (electrode placement, filters, feature formulas); a reasonable sample size for an EMG study (n=153); an appropriate statistical approach, including stratified analyses; explicit limitations; and ethics/funding declarations. **Main weaknesses / potential bias**: lack of reported sampling frame/response rate (items 5,7,13,14) limits assessment of selection bias and generalizability; no a priori power/sample size calculation (item 3); no sharing of raw data or code (availability statement conflicts: “No datasets were generated or analyzed during the current study” — ambiguous) which	**QUADAS-2** (if index intended as diagnostic test; assess applicability) **Overall judgment:** Moderate risk of bias with moderate applicability concerns**.**	The study provides valuable, well-documented technical findings and the largest EMG dataset reported to date for sarcopenia screening. The ICDMC shows promising discriminative signal at low contraction intensity (20% MVC) across several time-domain features. Because of moderate bias risks (index test interpretation not explicitly blinded) and applicability concerns due to group imbalances and age differences, results should be considered promising but preliminary.

				reduces reproducibility (items 11 & Data availability p.17). Confidence intervals are not reported (item 10).		
PUBMED 10 / (Dias et al. 2019) / D	Neural control properties of the external anal sphincter in young and elderly women	physiological cross-sectional + measurement.	**COSMIN** (measurement properties, decomposition validity) **Judgments: Overall -** Inadequate	**Strengths:** Design and procedures well described. **Limitations:** Sample small and representativeness limited; does not meet COSMIN large sample expectations. Theoretical rationale exists, but there is no formal content validity work with patients or experts, nor any documented qualitative methods. Between group testing performed, but no formal measurement invariance/DIF analyses; reliability not assessed; measurement error metrics absent; Responsiveness not assessed in this cross sectional study; longitudinal evaluation required.	**AXIS** (cross-sectional sampling & external validity) **Reporting quality: Good.**	The study is methodologically rigorous for an electrophysiology decomposition study: clear objectives, appropriate HD-sEMG hardware and a high sampling rate (5 kHz), use of a modern decomposition algorithm (KmCKC), STA reconstruction, and appropriate statistical tests. The results are internally consistent, and interpretations are cautious and supported. **Main weaknesses:** small sample size (n=27 analyzed), absence of an a priori power calculation, limited details on recruitment/response rates, and lack of concurrent force measurement (which limits the direct linking of MU properties to functional sphincter force). Confidence intervals for estimates are not presented. **Conclusion:** The paper provides a credible, novel, noninvasive characterization of age-related EAS motor unit changes; the findings are promising but exploratory and require replication in larger cohorts and patient populations

PUBMED 8 /(Marsall et al.), 2020 / A	Quadriceps muscle electromyography activity during physical activities and resistance exercise modes in younger and older adults	cross-sectional comparative experimental tasks.	**AXIS Reporting Quality:** Good	The study is well designed and well reported for experimental questions regarding EMG and age; the results are valid within the stated limits (small N but adequately powered) and relevant to practice (EB as a pragmatic RET). **Main strengths:** a priori power calculation (item 3), detailed methodological protocol (items 11, 2.5–2.6), appropriate statistical plan and effect sizes (2.7), explicit statement of limitations, and clear declarations regarding ethics and funding. **Limitations/areas for improvement:** sampling frame/recruitment process and response rate are not explicitly reported (items 5, 7, 13, 14); no confidence intervals provided (item 10); textile-EMG limitations acknowledged (item 9); and raw data are not linked (data availability is not stated).	**COSMIN Overall risk of bias:** Moderate (some concerns).	**Strengths:** Clear a priori aims and hypotheses, appropriate experimental design for the questions (within-subject trials across modalities) and prespecified statistical plan (mixed ANOVA, corrections, effect sizes), supports construct validity and internal validity (Intro & Methods, pp.2–4). Measurement method is appropriate and justified. Sample size was justified by power analyses, and the authors recruited the planned numbers (power rationale and n per group reported), thereby reducing the risk of type II error for main contrasts. Results are clearly reported with effect sizes and appropriate corrections for multiple comparisons. **Limitations** increasing bias risk: Participants were recruited via convenience sampling (volunteers, community flyers/contacts), introducing selection bias. There is no comparative analysis of excluded versus included participants, limiting assessment of attrition bias. **Criterion validity:** no concurrent gold-standard comparator (e.g., intramuscular EMG or metabolic measures) was included, so external validation against a reference is lacking

PUBMED 4 /(Kumar et al. 2024) / C	sEMG-based Sarcopenia risk classification using empirical mode decomposition and machine learning algorithms	ML diagnostic/ prediction study.	**PROBAST (prediction model risk of bias)** **Riesgo de Sesgo (Calidad Metodológica):** ALTO; **Aplicabilidad:** MODERADAS	El estudio presenta un alto riesgo de sesgo en el Dominio 3 (Outcome). Los autores reconocen que no utilizaron el estándar clínico de oro para diagnosticar sarcopenia (como la fuerza de prensión manual o la masa muscular por DXA), sino un "proxy" basado en el IMC y en cuestionarios autoinformados. **Aplicabiidad**: Aunque la tecnología sEMG es aplicable, la población joven (20-60 años) no es adecuada para un estudio de sarcopenia (que suele afectar a mayores de 65 años). Además, la dependencia de cuestionarios subjetivos para la etiqueta de riesgo limita su utilidad clínica inmediata sin una validación basada en criterios médicos internacionales (EWGSOP2).	**TRIPOD** / checklist for ML reporting; check for data leakage and subject-wise split **Overall consensus:** Partially adherent to TRIPOD.	**Stregths:**Clearly reported study aims, participant selection, sEMG acquisition, feature extraction (including comprehensive feature lists and EMD use), feature selection, model types, and internal LOSO validation with detailed performance metrics and confusion matrices. **Limitations:**(1) outcome labeling/ground-truth definition insufficiently specified (grouping based on BMI + self-reported activity without precise cutpoints in Methods); (2) absence of formal sample-size justification; (3) no reporting of uncertainty (confidence intervals) or calibration assessment for model performance; (4) no external validation; (5) limited reproducibility resources (no public code, model files, or data availability statement); (6) no explicit ethics committee approval statement. These omissions reduce transparency and reproducibility per TRIPOD.

PUBMED1 / (Sepulveda et al., 2025) / A	Are There Differences in Postural Control and Muscular Activity in Individuals with COPD and with and Without Sarcopenia?	cross-sectional diagnostic/comparative clinical study.	**QUADAS-2** (if treating as diagnostic accuracy/clinical applicability) **Overall reporting quality: High risk of bias (with moderate applicability concerns)**	**Strength:** The index tests themselves are well described and use standard equipment and procedures (force platform BIOMEC400; Trigno EMG; acquisition parameters reported, pp.3–4), so applicability to clinical/research settings is acceptable. Reference Standard has a low risk of bias; low applicability concerns. **Limitations:** Convenience sampling and single center recruitment limit representativeness and introduce selection bias, which also creates moderate concerns about the applicability of the results to broader COPD populations. High risk of bias for interpretation; low applicability concerns for conduct.	**NOS** (if treated as cohort-like clinical comparison) **Overall reporting quality: Good for a clinical observational study.**	**Strengths:** Good for a clinical observational study. The investigators used validated measurement instruments (force platform, Delsys EMG), applied appropriate diagnostic criteria for sarcopenia (EWGSOP) with population-specific cutoffs, and presented coherent results linking sarcopenia to balance impairment and altered muscle activation in COPD patients. Statistical methods are appropriate, and results are internally consistent. **Limitations:** small convenience sample (n=35; sarcopenic n=13), lack of recruitment/response flow, limited power for some comparisons, cross-sectional design precluding causal inference, and reliance on BIA for muscle mass (methodologically acceptable but less precise than DXA). Confidence intervals are not presented for primary effect estimates.

Discussion

This comparative analysis examined consistency between the GRADE judgments recorded in Table S3.1 (with detailed justifications in the Appendix) and the risk-of-bias (RoB) assessments summarized in Table S3.3 for twelve studies (IEEE 1; PUBMED 1, 4, 5, 8–15). The exercise focused on whether domain-level judgments in Table 1 (random sequence generation, allocation concealment, blinding, outcome assessment, incomplete data, selective reporting, other bias) aligned with the overall RoB results and tool-specific judgments reported in Table S3.3 (PROBAST, QUADAS-2, AXIS, NOS, COSMIN, TRIPOD adherence, etc.).

Key themes emerged from the article-by-article comparison:

1. Sources of agreement and why they occurred

Studies with clear, well-documented methods, objective outcomes, and controlled within-subject designs tended to show concordant judgments across tables (e.g., PUBMED 1, PUBMED 8, PUBMED 10, PUBMED 13, PUBMED 14, PUBMED 15). In these cases, Table S3.1's domain ratings matched Table S3.3's tool-based conclusions because both relied on the same observable strengths: pre-specified protocols, objective measurement, transparent exclusions, and, where present, clear reporting of validation methods.

When a single clear methodological limitation drove risk (e.g., lack of external validation or small sample size), both tables tended to consistently recognize that limitation — although the label assigned (Unclear vs High) sometimes differed in nuance.

2. Sources of discrepancy and their patterns

- Under-recognition of issues specific to predictive modeling: Discrepancies most frequently occurred in studies using machine learning or predictive modeling (IEEE 1, PUBMED 4, PUBMED 9). Table S3.1 often marked standard cross-validation/partitioning practices as Low risk without fully accounting for ML-specific pitfalls that PROBAST penalizes, such as unclear outcome/ground-truth definitions, feature selection or preprocessing performed outside nested CV, or lack of external validation.

- The result is that Table S3.1 appears optimistic in the selection/allocation/randomization domains, while Table S3.3 (PROBAST) flags a higher overall risk. Ground-truth and labeling issues: Studies relying on manual annotation, subjective labeling, or complex signal decomposition (PUBMED 5, PUBMED 11) sometimes had Table 1 mark several domains as Low while Table S3.3 emphasized the impact of manual processing on detection bias and measurement properties. When detection depended on substantial manual intervention, Table S3.3's tools raised concerns that Table 1 did not always fully translate into a higher or clearly stated global risk.

- Other bias under-specified: In several cases, Table S3.1 noted: "Other bias = Unclear" while Table S3.3 reached a High or Moderate overall rating because limitations noted under "Other bias" (e.g., single-center cohorts, overfitting risk, high

dimensionality relative to sample size) materially affected applicability and model validity. The vagueness of the "Other bias" cell made Table 1's contribution to a global judgment less transparent.

- Inconsistent granularity: Table S3.3 tool outputs frequently consider construct validity, applicability, and model development procedures that require domain-specific items (e.g., cross-validation nesting, gold-standard definitions) not explicitly included in Table S3.1's standard RoB domains. This mismatch in granularity can produce apparent incongruence even where the underlying evidence is similar.

3. Consequences of mismatches

- Underestimation of risk in Table S3.1 may lead readers to overconfident interpretation of evidence and inappropriate recommendations for clinical application or further research prioritization.
- Overly generic entries in the "Other bias" column reduce transparency and make it difficult to reconcile domain-specific concerns with the more structured tool outputs in Table S3.3.
- Failure to explicitly weigh the impact of detection/labeling concerns on global judgments can obscure the need for replication or external validation before adoption.

4. Principles followed for guide harmonization

- Make tool-specific, study-type critical items explicit in Table 1C. For example, for ML studies include: (a) whether feature selection was nested within cross-validation, (b) definition and validation of the ground-truth/outcome, (c) presence/absence of external validation, and (d) sample-size-to-parameter ratio or dimensionality concerns.
- In Table S3.1 "Other bias" with vague concept that could be robusted with entries with short, specific descriptions of the concrete problem and an explicit statement about how that problem affects the overall RoB judgment (e.g., "High risk — single-center, n=40, no external validation → likely overfitting; downgrades applicability").
- Where a domain is driven by a single critical methodological flaw (e.g., manual labeling that affects outcome measurement), elevate that domain's rating and explicitly link that elevation to the consensus RoB conclusion in Table S3.3.

5. Concrete actions to perform after consensus

Priority (A): Immediate corrections to Table S3.1 for discordant studies

1) PUBMED 4 (Kumar): Reclassify key domains to reflect PROBAST's High rating.
- Actions: Change Random sequence/Allocation/Selective reporting from Low to Unclear/High where labeling, ground-truth definition, or potential leakage was identified. Expand "Other bias" to state specifics: "High — inadequate description of gold-standard labeling; potential feature-selection leakage; no external validation." Add a short justification line linking these changes to Table 3's PROBAST high-risk conclusion.

2) PUBMED 11 (Hu) and PUBMED 5 (He): Make detection bias explicit and adjust impact on global grading.
- Actions: For both, keep detection marked High where manual decomposition/manual annotation was required, and explicitly state how this High increases the global RoB (e.g., mark global as Some concerns/High rather than remaining Low). Add details on the extent of manual processing (% of data affected) if available.

3) IEEE 1, PUBMED 9, PUBMED 12: Raise "Other bias" or Allocation/Applicability concerns to Unclear/Some concerns.
- Actions: Update "Other bias" description to explicitly mention lack of external validation, small sample or cohort-specific applicability, and (for ML studies) high dimensionality / overfitting risk. Note how that affects the overall RoB.

Priority (B): Structural improvements to Table 1 and reporting templates

- Addition of Information in Table 4 used in the GRADE analysis. This will make reconciliation explicit and auditable.
- Mention that the generic "Other bias" entries in GRADE format with structured prompts: "Describe the bias (1–2 phrases); Rate impact (Low/Unclear/High); State linkage to overall RoB."

Priority (C): Transparency and documentation actions

- Table 4 is an annotated Table 1 with changes: For every corrected study, include a brief footnote recording the specific evidence from the Appendix or methods that justified the change (e.g., "Ground-truth labeling performed by single rater without reliability study — justifies elevating detection bias").
-It is provided a folder in the GITHUB for each Table 3C tool's rationale (e.g., PROBAST domain-level drivers).

Table S3.4. Changes proposed for improving the GRADE report.

File	*Original Table 1 entry (selected domains)*	*Table 3C RoB result (tool & key drivers)*	*Changes applied to Table 1 (domain → new rating)*	*New/expanded "Other bias" text (1–2 phrases)*	*Justification / Evidence (short)*	*Impact on overall RoB*
PUBMED 4 (Kumar)	Random sequence: Low; Allocation: Low; Selective reporting: Low; Other bias: Unclear	PROBAST — High risk (drivers: unclear gold-standard labeling; possible feature-selection leakage; no external validation)	Random sequence: Low → Unclear/High; Allocation: Low → Unclear/High; Selective reporting: Low → Unclear/High	High Inadequate description of gold-standard labeling; potential feature-selection leakage; no external validation	Methods: labeling procedure not described; feature-selection steps not nested in CV; no external cohort reported in Appendix Table 3	Raises overall RoB to High (aligned with PROBAST)
PUBMED 11 (Hu)	Detection: Low; Other bias: High (small sample)	AXIS/COSMIN — Moderate/Inadequate (drivers: manual MU decomposition; small sample limits measurement properties)	Detection: Low → High;<br><br>Global RoB: Low/Unclear → Some concerns/High	High Manual decomposition/annotation of motor units; small sample size limits measurement properties	Appendix: methods describe manual decomposition without inter-rater reliability; n small; COSMIN flagged inadequate measurement properties	Increases global RoB to Some concerns / High depending on detection impact
PUBMED 5 (He)	Detection: High;<br><br>Other bias: Unclear	AXIS/QUADAS-2 — Moderate (drivers: manual MU decomposition; applicability concerns)	Detection: High (confirmed); Global RoB: Low/Unclear → Some concerns/High (explicitly linked to detection)	High Manual outcome processing for signal decomposition; potential assessor-dependence	Appendix: manual validation noted; no reliability metrics; AXIS flagged applicability concerns	Explicitly mark global as Some concerns (or High if detection affects primary outcome validity)

IEEE 1 (Song)	Many domains Low; Other bias: Unclear (overfitting, no external validation)	PROBAST → Moderate/High (drivers: overfitting risk, lack of external validation, small sample/high dimensionality)	Other bias: Unclear → Some concerns/High;<br><br>Allocation/Applicability: Unclear → Some concerns	Some concerns overfitting risk, high dimensionality relative to sample; no external validation reported	Appendix: single-center cohort; model complexity high; no external test set in Table 3	Raises global RoB to Moderate/High (align with PROBAST conclusion)
PUBMED 9 (Li)	Domains mostly Low; Other bias: Unclear	PROBAST/TRIPOD — Moderate / Some concerns (drivers: preselection/exclusions; no nested CV; limited external validation)	Other bias: Unclear → Some concerns;<br><br>Allocation/Selective reporting: Low → Unclear	Some concerns Exclusions and ground-truth construction; limited external validation; potential leakage	Appendix: exclusions reported; feature-selection not nested; TRIPOD adherence partial	Increase overall RoB to Some concerns / Moderate
PUBMED 12 (Cai)	Random sequence: Low; Allocation: Unclear; Other bias: Low	AXIS/NOS — Moderate (drivers: small n, limited external generalizability)	Other bias: Low → Unclear/Some concerns; Allocation: Unclear → Unclear/Some concerns	Some concerns — small sample size; single-center recruitment limits external validity	Appendix: NOS score fair (6/9); AXIS notes limited generalizability	Adjust global RoB to Moderate / Some concerns

References and Resources

1) RoB 2: Sterne et al., 2019. https://www.bmj.com/content/366/bmj.l4898 ; herramientas y guía disponibles en el sitio web de Cochrane (RoB 2: Excel/interactive tool).
2) ROBINS-I: Sterne et al., 2016. https://www.bmj.com/content/355/bmj.i4919 ; Cochrane guidance.
3) Newcastle–Ottawa Scale (NOS): Wells et al. (Ottawa) — https://www.ohri.ca/programs/clinical_epidemiology/oxford.asp Newcastle-Ottawa Scale (NOS) | Ottawa Hospital Research Institute
4) Downes, M. J., Brennan, M. L., Williams, H. C., & Dean, R. S. (2016). Development of a critical appraisal tool to assess the quality of cross-sectional studies (AXIS). *BMJ open*, *6*(12), e011458.
5) Whiting, P. F., Rutjes, A. W., Westwood, M. E., Mallett, S., Deeks, J. J., Reitsma, J. B., ... & QUADAS-2 Group*. (2011). QUADAS-2: a revised tool for the quality assessment of diagnostic accuracy studies. *Annals of internal medicine*, *155*(8),

529-536.(original paper) https://www.acpjournals.org/doi/full/10.7326/0003-4819-155-8-201110180-00009 y herramienta en

6) Wolff, R. F., Moons, K. G., Riley, R. D., Whiting, P. F., Westwood, M., Collins, G. S., ... & PROBAST Group†. (2019). PROBAST: a tool to assess the risk of bias and applicability of prediction model studies. *Annals of internal medicine*, *170*(1), 51-58.
7) https://www.acpjournals.org/doi/full/10.7326/M18-1377?download=true&journalCode=aim. Ultima versión: https://www.probast.org/
8) Moons KGM, Damen JAA, Kaul T, et al. PROBAST+AI: an updated quality, risk of bias, and applicability assessment tool for prediction models using regression or artificial intelligence methods. BMJ 2025;388:e082505 doi:10.1136/bmj-2024-082505
9) COSMIN: https://cosmin.nl/ (manual y checklist), Tool Checklists for Assessing Study Qualities • COSMIN and Instructions: EE-document_version-1.1.pdf
10) Collins, G. S., Reitsma, J. B., Altman, D. G., & Moons, K. G. (2015). Transparent reporting of a multivariable prediction model for individual prognosis or diagnosis (TRIPOD): the TRIPOD statement. *Journal of British Surgery*, *102*(3), 148-158.
11) Guyatt, G., Oxman, A. D., Akl, E. A., Kunz, R., Vist, G., Brozek, J., ... & Schünemann, H. J. (2011). GRADE guidelines: 1. Introduction—GRADE evidence profiles and summary of findings tables. *Journal of clinical epidemiology*, *64*(4), 383-394.
12) Shao, S. C., Kuo, L. T., Huang, Y. T., Lai, P. C., & Chi, C. C. (2023). Using Grading of Recommendations Assessment, Development, and Evaluation (GRADE) to rate the certainty of evidence of study outcomes from systematic reviews: A quick tutorial. *Dermatologica Sinica*, *41*(1), 3-7.
