# Supplementary material for: Electrophysiological Signatures of Sarcopenia: A Systematic Review of sEMG Features, Fatigue Indices and AI-Based Classifiers
Source: Sensors (Basel). 2026 Aug 13;26(16):5121. doi: 10.3390/s26165121 (PMC13517259; doi:10.3390/s26165121)
Supplement: Supplementary file 1 [file sensors-26-05121-s001.zip › File S4.pdf]

S4. Meta-analysis of handgrip-related measures in older adults with and without sarcopenia

The purpose of this supplementary meta-analysis was to quantitatively contextualize the handgrip protocol used as a functional reference for assessing sarcopenia and to provide an evidence-based framework for interpreting sEMG-derived neuromuscular measurements. Handgrip-based assessment is particularly relevant because it provides a clinically interpretable measure of muscle performance, whereas sEMG provides complementary information regarding muscle activation and neuromuscular function.

Data sources

Upon analysis, only 6 of 12 (50%) articles use handgrip MVC as a clinical reference to support differences in health conditions for sEMG assessment.

Table S4.1. Articles considered with enough information for the meta-analysis of handgrip force

[1]	*He(2024), "Multi-channel EMG manifestations of upper-extremity muscle coordination imbalance among community-dwelling sarcopenic seniors"*
[2]	Li (2024), "Exploration of a machine learning approach for diagnosing sarcopenia among Chinese community-dwelling older adults using sEMG-based data"
[3]	Hu(2021), "Characteristics of the Electrophysiological Properties of Neuromuscular Motor Units and Its Adaptive Strategy Response in Lower Extremity Muscles for Seniors with Pre-Sarcopenia: A Preliminary Study"
[4]	Ebenbichler(2020), "Age-specific differences in the time-frequency representation of surface electromyographic data recorded during a submaximal cyclic back extension exercise: a promising biomarker to detect early signs of sarcopenia"
[5]	Sepulveda-Loyola W. (2025), "Are There Differences in Postural Control and Muscular Activity in Individuals with COPD and with and Without Sarcopenia?"
[6]	Cai (2025), "Lower extremity muscle activation patterns in sarcopenic older adults during physical performance tests: implications for rehabilitation approaches"

Focusing on elderly with and without Sarcopenia, 2 papers were discarded, and [1-3] and [5] were considered. Then three prespecified subgroup analyses were performed: elderly females, elderly males, and the overall elderly population. The female subgroup included two independent study estimates; the male subgroup included two; and the overall elderly subgroup included four. The study estimates contributed to these subgroup analyses were not pooled across subgroups. In particular, the He (2024) and Li (2024) studies contributed sex-specific estimates and overall elderly estimates; therefore, combining all subgroup estimates into a single overall meta-analysis would

have resulted in non-independent reuse of study-level evidence. Consequently, no additional overall pooled estimate across the three subgroups was calculated.

Table S4.2. Hand Graping (Kg) analysis of random effects comparing the control and sarcopenia condition. MD = mean difference, SE = standard error, CI = [CI low, CI high], and weighted percentage in the metrics.

Study	*Subgroup*	*N Control*	*Mean Control*	*SD Control*	*N Case*	*Mean Case*	*SD Case*
He et al. (2024)	*Elderly Female Sarcopenia and Elderly Female control*	68	20.4	3.6	36	14.8	2.6
Li et al. (2024)		29	22.4	3	31	14.4	2.8
He(2024)	*Elderly Male Sarcopenia and Elderly Male control*	30	32.1	5.3	19	24.1	2.9
Li (2024)		16	34.1	5.4	17	23.8	2.2
Sepulveda (2025)	*Elderly Sarcopenia and Elderly Control*	22	24.5	7.4	13	19.8	5.9
He (2024)		98	23.9	7	55	18	5.2
Li (2024)		45	26.6	6.9	48	17.7	5.2
Hu(2021)		5	29.6	8.88	5	20	3.74

Methods

1. Effect measure

The effect measure was the mean difference (MD), calculated as the difference between the control and sarcopenia groups:

$$MD_i = \bar{X}_{Control,i} - \bar{X}_{Sarcopenia,i}.$$

Because the outcomes were expressed on a common measurement scale across the included studies, the raw mean difference was retained rather than a standardized mean difference.

The within-study sampling variance was calculated as:

$$V_i = \frac{SD^2_{Control,i}}{n_{Control,i}} + \frac{SD^2_{Sarcopenia,i}}{n_{Sarcopenia,i}}.$$

The corresponding standard error was:

$$SE_i = \sqrt{V_i}.$$

2. Random-effects model

A random-effects meta-analysis was used because the included studies were expected to differ in sample characteristics, measurement protocols, and clinical contexts. Under the random-effects framework, the pooled estimate represents the average effect across the distribution of underlying study-specific effects rather than assuming a single common true effect.

Between-study variance (τ^2) was estimated using both the DerSimonian–Laird (DL) and restricted maximum likelihood (REML) approaches. The DL estimator was selected as the primary implementation because it provides the classical moment-based random-effects estimator, while REML was included as a sensitivity estimator of between-study variance. The DL method follows the classical formulation of DerSimonian and Laird (DerSimonian, 1986).

The pooled mean difference was calculated using inverse-variance random-effects weights:

$$w_i = \frac{1}{V_i + \tau^2}.$$

3. Heterogeneity

Statistical heterogeneity was evaluated using Cochran's Q statistic and quantified using $I^2$and τ^2. I^2represents the proportion of observed variability attributable to between-study heterogeneity rather than within-study sampling error. The interpretation of I^2 was considered together with $Q$and τ^2, rather than as an isolated criterion.

4. Prediction intervals

A 95% prediction interval was calculated for the overall elderly subgroup because four independent studies were available. The prediction interval was interpreted as an estimate of the range in which the underlying effect of a future comparable study might plausibly lie, rather than as a confidence interval for the pooled mean. Prediction intervals were not calculated for the sex-specific analyses because each contained only two studies. Given the small number of studies in the overall analysis of the elderly (k = 4), the prediction interval was considered exploratory and interpreted with caution. This caution is consistent with recommendations that prediction intervals become less reliable when the number of studies is small.

Primary results

1. Overall elderly

Four independent studies contributed to the overall elderly subgroup. The DL model yielded a pooled MD of 6.949 (95% CI: 4.866–9.033), with $\tau^2 = 1.632$ and $I^2 = 37.54\%$. The REML model yielded a highly similar pooled MD of 6.953 (95% CI: 4.774–9.131), with $\tau^2 = 1.950$ and I^2 = 4.80%. Cochran's test did not provide evidence of statistically significant heterogeneity ($Q = 4.803, df = 3, p = 0.187$).

The close agreement between DL and REML estimates supports the stability of the pooled mean difference with respect to the estimator of between-study variance.

2. Prediction intervals

The corresponding prediction intervals were approximately −0.20 to 14.10 under DL and −0.73 to 14.63 under REML. Because only four studies were available, these intervals were considered exploratory. Notably, although the 95% confidence interval for the pooled mean difference excluded zero, the prediction intervals included zero, indicating greater uncertainty regarding the magnitude and direction of the effect that might be observed in a future comparable study.

3. Sex-specific analyses

The elderly female subgroup included two studies and yielded a pooled MD of 6.761 (95% CI: 4.411 – 9.112), with $\tau^2 = 2.409$ and $I^2 = 83.65\%$. The elderly male subgroup also included two studies and yielded a pooled MD of 8.992 (95% CI: 6.759–11.224), with $\tau^2 = 0.902$and $I^2 = 34.10\%$. Because each sex-specific analysis was based on only two studies, these estimates were considered supportive rather than definitive, and no formal inference regarding differences between male and female pooled effects was performed.

Note: MD = mean difference (Control − Sarcopenia); CI = confidence interval; DL = DerSimonian–Laird; REML = restricted maximum likelihood; τ^2 = between-study variance; I^2 = proportion of total variability attributable to between-study heterogeneity; PI = Prediction interval; Q = Cochran's heterogeneity statistic.

4. Sensitivity analysis

A leave-one-study-out (LOSO) sensitivity analysis was performed by excluding Hu (2021), which contributed only five participants per group and had substantially larger sampling variance than the other studies. The pooled effect changed from 6.949 to

6.772 under DL and from 6.953 to 6.772 under REML, corresponding to changes of approximately 2.5% and 2.6%, respectively.

In contrast, τ^2 increased from 1.632 to 2.321 under DL and from 1.950 to 2.318 under REML. Thus, exclusion of the small study had little influence on the magnitude of the pooled effect but altered the estimated between-study heterogeneity. The sensitivity analysis therefore supports the stability of the estimated average effect while indicating uncertainty in the estimation of heterogeneity with the small number of available studies.

Table S4.3. Sensitivity analysis result of Hand Griping (Kg) analysis with and without Sarcopenia elderly group excluding Hu et al. study because of the sample size.

Scenario	θ	***CI low***	***CI high***	***PI low***	***PI hi***	τ^2	**Q**	I^2
Original_with_Hu_DL	6.949	4.866	9.0326	-0.201	14.100	1.632	4.803	37.542
Original_with_Hu_REML	6.952	4.773	9.131	-0.727	14.632	1.950		
Exclude_Hu_DL	6.771	4.410	9.132	-17.905	31.449	2.320	4.390	54.443
Exclude_Hu_REML	6.771	4.411	9.131	-17.892	31.435	2.317		

Note: MD = mean difference (Control − Sarcopenia); CI = confidence interval; DL = DerSimonian–Laird; REML = restricted maximum likelihood; τ^2 = between-study variance; I^2 = proportion of total variability attributable to between-study heterogeneity; PI = Prediction interval; Q = Cochran's heterogeneity statistic.

5. Independent computational validation.

The primary meta-analysis was implemented in MATLAB R2024b using custom code developed for the present study (MetaAnalysis_groups_final.m). To independently verify the statistical implementation, all study-level mean differences and sampling variances were re-analyzed in R version 4.6.1 using the *metafor* package (version 5.0-1). The rma() function was used with method="DL" and method="REML" under an inverse-variance random-effects model. “validation_metafor.R” is included in the SOFTWARE section in GITHUB link https://github.com/c4macho/LIPS_ARTICULOS

The independent implementation reproduced the MATLAB results for the pooled effect, confidence intervals, τ^2, Cochran's Q, and I^2, confirming the numerical consistency of the primary implementation.

Table S4.4. Hand Griping (Kg) analysis of random effects with the control and sarcopenia condition with software in MATLAB and verified with metafor.

Group	*k*	*Method*	*MD (MATLAB)*	*MD (R)*	τ^2 *(MATLAB)*	τ^2 *(R)*
Elderly Female	2	DL	6.761	6.761	2.409	2.409
Elderly Female	2	REML	6.761	6.761	2.409	2.409
Elderly Male	2	DL	8.992	8.992	0.902	0.902
Elderly Male	2	REML	8.992	8.992	0.902	0.902
Elderly	4	DL	6.949	6.949	1.632	1.632
Elderly	4	REML	6.953	6.953	1.950	1.950
Elderly no Hu	3	DL	6.772	6.772	2.321	2.321
Elderly no Hu	3	REML	6.772	6.772	2.318	2.318

The pooled MD for Elderly with and without Sarcopenia meta-analysis is summarized in Fig S4.1 and S4.2 using Dl and REML estimation methods of MD, respectively.

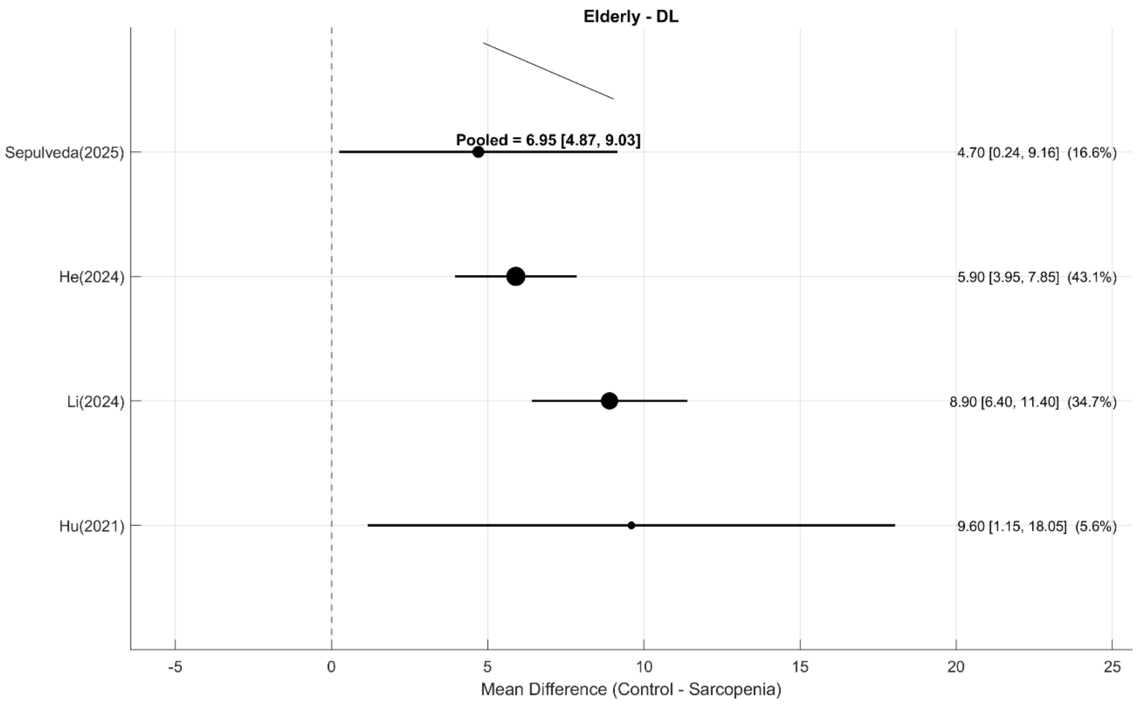

Fig. S4.1 Forest plot of elderly with and without sarcopenia response in a handgrip test with DL estimation.

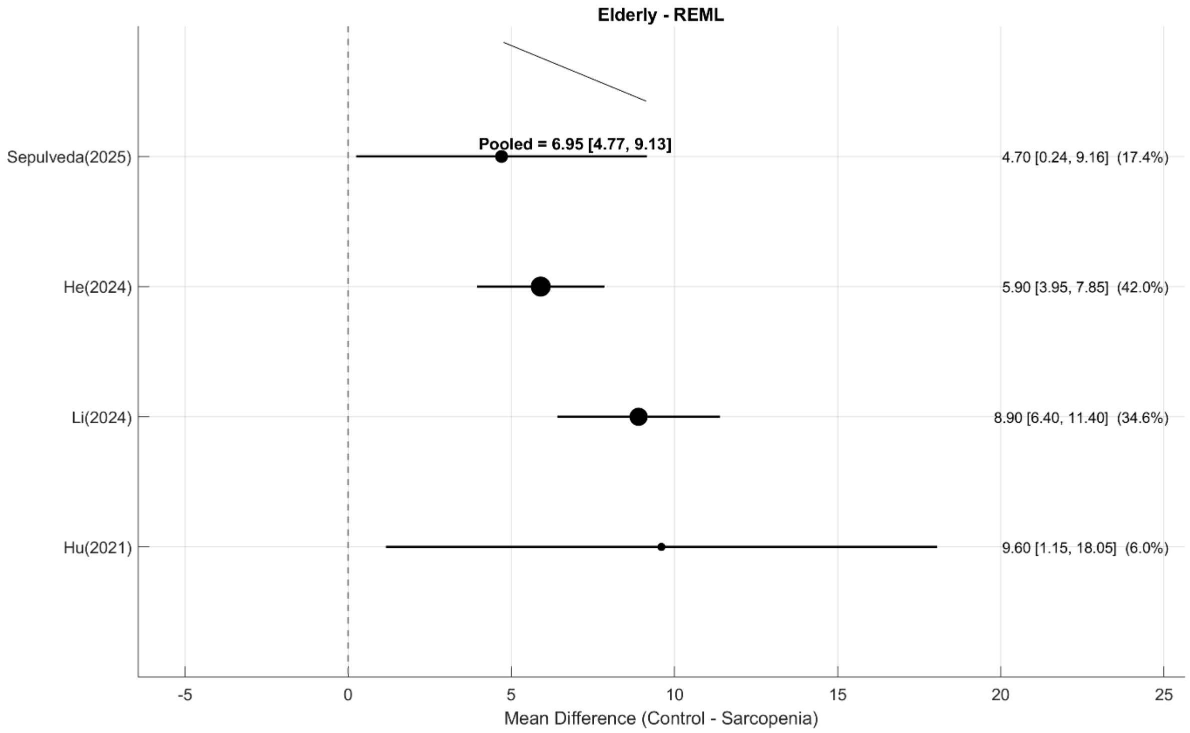

Fig. S4.2 Forest plot of elderly with and without sarcopenia response in a handgrip test with REML estimation.

Discussion

The meta-analysis provides quantitative support for a systematic difference in handgrip-related performance between older adults with and without sarcopenia. Across the four independent studies contributing to the overall elderly subgroup, the pooled mean difference was approximately 6.95 units, and the estimate was essentially unchanged when either DL or REML was used to estimate between-study variance. This consistency is relevant because it indicates that the observed group difference was not substantially dependent on the particular estimator of heterogeneity.

The moderate I^2 estimates (37.5% under DL and 41.8% under REML) indicate that a proportion of the variability among study estimates cannot be attributed solely to sampling error. However, the non-significant Cochran's Qtest should not be interpreted as evidence of complete homogeneity, particularly given the small number of studies. The female subgroup showed higher estimated heterogeneity than the male subgroup, but both analyses were based on only two studies and therefore should not be used to infer robust sex-specific differences.

The sensitivity analysis further indicated that the small study by Hu et al. had limited influence on the pooled effect size. Excluding this study changed the pooled estimate by only approximately 2.5%–2.6%. Nevertheless, the estimated between-study variance increased after exclusion, highlighting that heterogeneity estimates are substantially less stable than the pooled effect when only a small number of studies are available.

From a multimodal perspective, these findings provide a clinically interpretable reference for the sEMG measurements investigated in the present work. Handgrip performance reflects an integrated functional manifestation of neuromuscular capacity, whereas sEMG can characterize complementary electrophysiological features associated with muscle activation and fatigue. Therefore, the meta-analytic evidence does not establish handgrip as a substitute for electrophysiological assessment; rather, it supports its use as a clinically meaningful reference for interpreting multimodal neuromuscular measurements.

The prediction intervals illustrate an additional limitation. Although the confidence interval around the pooled effect excluded zero, the prediction intervals included zero, indicating that uncertainty regarding the effect in a future comparable study remains substantial. This distinction is particularly important for interpreting the present analysis because only four studies contributed to the overall elderly subgroup.

Conclusions. The meta-analysis provides quantitative evidence of a positive mean difference in handgrip-related performance between older adults without and with sarcopenia. The pooled estimate was approximately 6.95 units and was highly consistent between the DL and REML random-effects models. The sensitivity analysis showed that exclusion of the small Hu et al. study produced only a minor change in the pooled effect, supporting the stability of the estimated average difference.

Nevertheless, the limited number of available studies restricts the precision with which between-study heterogeneity and prediction of future effects can be characterized. Accordingly, the present meta-analysis should be interpreted as supportive clinical context rather than as definitive evidence of a universal effect size.

Within the multimodal framework, handgrip assessment provides a clinically interpretable functional reference, while sEMG contributes complementary information regarding neuromuscular activation and fatigue. Their integration may therefore strengthen the characterization and interpretation of sarcopenia-related functional impairment.

References

DerSimonian R, Laird N. Meta-analysis in clinical trials. Control Clin Trials. 1986 Sep;7(3):177-88. doi: 10.1016/0197-2456(86)90046-2. PMID: 3802833.

Cochrane Org., **Chapter 10: Analysing data and undertaking meta-analyses, consulted on 30th April 2025 at** https://www.cochrane.org/authors/handbooks-and-manuals/handbook/current/chapter-10
